# 3D Vessels-on-Chip using isogenic hiPSC-derived VSMCs reveal NOTCH3-driven alterations in brain small vessel disease

**DOI:** 10.1016/j.stemcr.2026.102863

**Published:** 2026-03-26

**Authors:** Marc Vila Cuenca, Theano Tsikari, Minne N. Cerfontaine, James L. Gallant, Francijna E. van den Hil, Marga J. Bouma, Kyra L. Dijkstra, Gido Gravesteijn, Antoine A.F. de Vries, Christine L. Mummery, Julie W. Rutten, Saskia A.J. Lesnik Oberstein, Valeria V. Orlova

**Affiliations:** 1 Department of Clinical Genetics, Leiden University Medical Center, Leiden, the Netherlands; 2 Department of Anatomy & Embryology, Leiden University Medical Center, Leiden, the Netherlands; 3 Leiden University Medical Center hiPSC Center, Leiden, the Netherlands; 4 Department of Pathology, Leiden University Medical Center, Leiden, the Netherlands; 5 Department of Cardiology, Leiden University Medical Center, Leiden, the Netherlands

**Keywords:** hiPSC-derived vascular smooth muscle cells, hiPSC-VSMCs, hereditary brain small vessel disease, SVD, CADASIL, NOTCH3, microfluidics, vessels on chip, disease modeling

## Abstract

cerebral autosomal dominant arteriopathy with subcortical infarcts and leukoencephalopathy (CADASIL) is a hereditary brain small vessel disease caused by pathogenic variants in the *NOTCH3* gene, leading to NOTCH3 protein accumulation and degeneration of vascular smooth muscle cells (VSMCs). Here, we developed a CADASIL 3D Vessel-on-Chip model using either primary brain VSMCs or human induced pluripotent stem cell (hiPSC)-derived VSMCs from CADASIL patients and isogenic controls. In 3D co-culture with hiPSC-derived endothelial cells, both primary and hiPSC-derived CADASIL VSMCs exhibited disease-relevant morphological abnormalities, increased NOTCH3 and contractile protein levels, and altered intracellular Ca^2+^ dynamics that were not observed under conventional 2D culture. PDGFRβ, a downstream NOTCH3 target, was upregulated and correlated with NOTCH3 protein levels in both 3D models and CADASIL patient brain tissue. Pharmacological inhibition of NOTCH3 cleavage reduced NOTCH3 protein levels and rescued CADASIL VSMC phenotypic abnormalities. In conclusion, this 3D Vessel-on-Chip model robustly shows CADASIL pathology-relevant readouts and provides a platform for mechanistic studies and therapeutic testing.

## Highlights

• CADASIL 3D Vessel-on-Chip using patient primary brain and hiPSC-derived VSMCs
• CADASIL VSMCs show disease phenotypes in 3D not observed in 2D culture
• CADASIL VSMCs display increased NOTCH3 and altered phenotypes in 3D Vessel-on-Chip
• NOTCH3 cleavage inhibition reduces NOTCH3 levels and rescues VSMC phenotypes

## Graphical abstract

**Figure:**
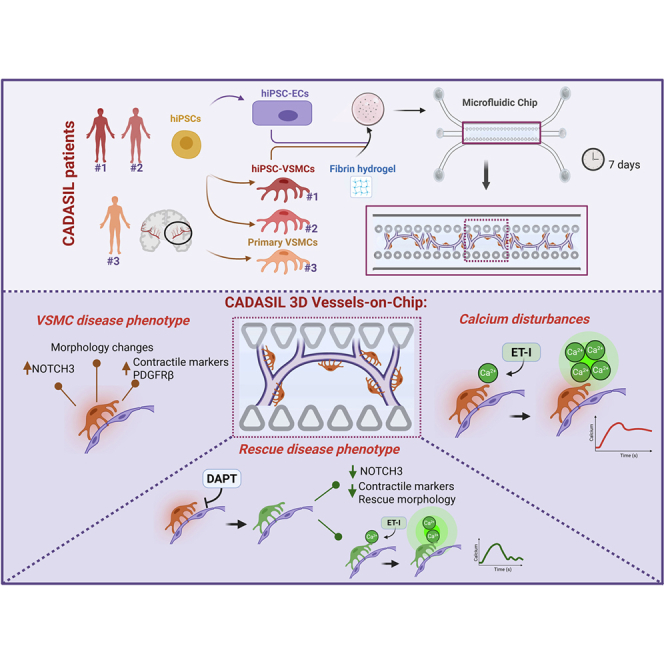

## Teaser

Orlova and colleagues present a 3D Vessel-on-Chip model for CADASIL using primary brain and hiPSC-derived VSMCs from patients and isogenic controls. The model reveals increased NOTCH3 and contractile protein levels, altered functional dynamics, and phenotypic rescue upon pharmacological inhibition of NOTCH3 cleavage.

## Introduction

The most prevalent genetic brain small vessel disease (SVD) is cerebral autosomal dominant arteriopathy with subcortical infarcts and leukoencephalopathy (CADASIL), a condition driven by pathogenic *NOTCH3* variants causing toxic gain of function through NOTCH3 protein aggregation in the blood vessel wall (Joutel et al. 1996, 2000). These *NOTCH3* variants occur in approximately 1 in 300 individuals worldwide and are associated with a broad spectrum of SVD severity (Rutten et al. 2014, 2016, 2020; Hack et al., 2022). CADASIL represents the severe end of the NOTCH3-associated SVD spectrum, with middle-age onset of stroke and vascular dementia (Wardlaw et al. 2019; Dupre, Drieu, and Joutel 2024). Disease-modifying therapies are currently only in pre-clinical development (Oliveira et al., 2023; Ghezali et al., 2018; Machuca-Parra et al., 2017).

The cell types primarily affected in CADASIL are vascular smooth muscle cells (VSMCs) and pericytes. NOTCH3 is a transmembrane receptor, which is predominantly expressed by VSMCs (Chabriat et al., 2009), and is essential for maintaining their differentiated state (Romay et al., 2024; Domenga et al., 2004). Endothelial cells (ECs) modulate NOTCH3 signaling through interactions with its ligands, such as Jagged and Delta-like proteins (Domenga et al., 2004; Villa et al., 2001). In CADASIL, NOTCH3 signaling seems to be largely preserved (Dupre, Drieu, and Joutel 2024; Dupre et al., 2024; Hack et al., 2023), while the ectodomain of the mutant NOTCH3 protein (NOTCH3^ECD^) accumulates in the extracellular space (Joutel et al., 2000), with sequestration of extracellular matrix proteins (Zellner et al., 2018; Capone et al., 2016; Dupre, Drieu, and Joutel 2024), leading to vessel wall pathology characterized by degeneration of VSMCs (Joutel et al., 2000; Dupre, Drieu, and Joutel 2024; Chabriat et al., 2009).

Multiple *in vitro* models for CADASIL have been developed to investigate the disease mechanisms, but each has faced challenges in recapitulating VSMC and vascular pathology. Moreover, many models use primary cells derived from patients, which have limited availability (Dupre, Drieu, and Joutel 2024; Tikka et al., 2012; Neves et al., 2019; Ihalainen et al., 2007). Efforts to model CADASIL using patient-derived human induced pluripotent stem cells (hiPSCs) have shown promise (Ahn et al., 2024; Kelleher et al., 2019; Ling et al., 2019), but thus far, diseased hiPSC derivatives have only been compared with non-isogenic healthy controls: different genetic background may mask or erroneously suggest the presence of disease-related features, which actually only reflect line-to-line differences. In addition, these models are frequently 2D cell cultures, which fail to emulate the integrated, complex, and multicell-type composition of the human vasculature. Vessel-on-Chip technology is emerging as an innovative approach offering standardized 3D engineered microscalable systems designed to replicate the vascular cellular microenvironment. Previously, we developed a 3D Vessel-on-Chip model that integrates ECs and VSMCs derived from hiPSC, effectively recapitulating the multicellular architecture of human vasculature (Vila Cuenca et al., 2021). Using isogenic, patient-specific hiPSC-derived vascular cells, we demonstrated that this platform can effectively capture hereditary vascular diseases (Orlova et al., 2022).

Here, we investigated whether this system would support modeling of other brain SVDs, such as CADASIL. We developed a CADASIL 3D Vessel-on-Chip model using either primary brain VSMCs or hiPSC-derived VSMCs from CADASIL patients, along with their isogenic controls. We found that CADASIL VSMCs exhibit increased NOTCH3 protein levels when in contact with ECs in the 3D Vessel-on-Chip environment, which was associated with changes in VSMC morphology, altered expression of contractile proteins, and actin cytoskeleton and focal adhesion disorganization. PDGFRβ levels increased in parallel with NOTCH3, which reflected findings in CADASIL patient brain tissue. Additionally, CADASIL VSMCs showed altered Ca^2+^ dynamics in the 3D Vessel-on-Chip. Finally, inhibition of NOTCH3 cleavage reduced NOTCH3 protein levels and restored VSMC morphological and functional differences. Thus, we show that pathogenic *NOTCH3* variants lead to phenotypic changes in VSMCs upon interaction with ECs, which can be reversed by targeting NOTCH3 cleavage. Our hiPSC-derived CADASIL 3D Vessel-on-Chip model provides a robust platform for *in vitro* pathomechanistic studies and testing potential disease-modifying therapeutic compounds.

## Results

### Primary brain VSMCs from CADASIL patients show phenotypic changes and increased NOTCH3^ECD^ protein in a 3D Vessel-on-Chip model

**Figure 1:**
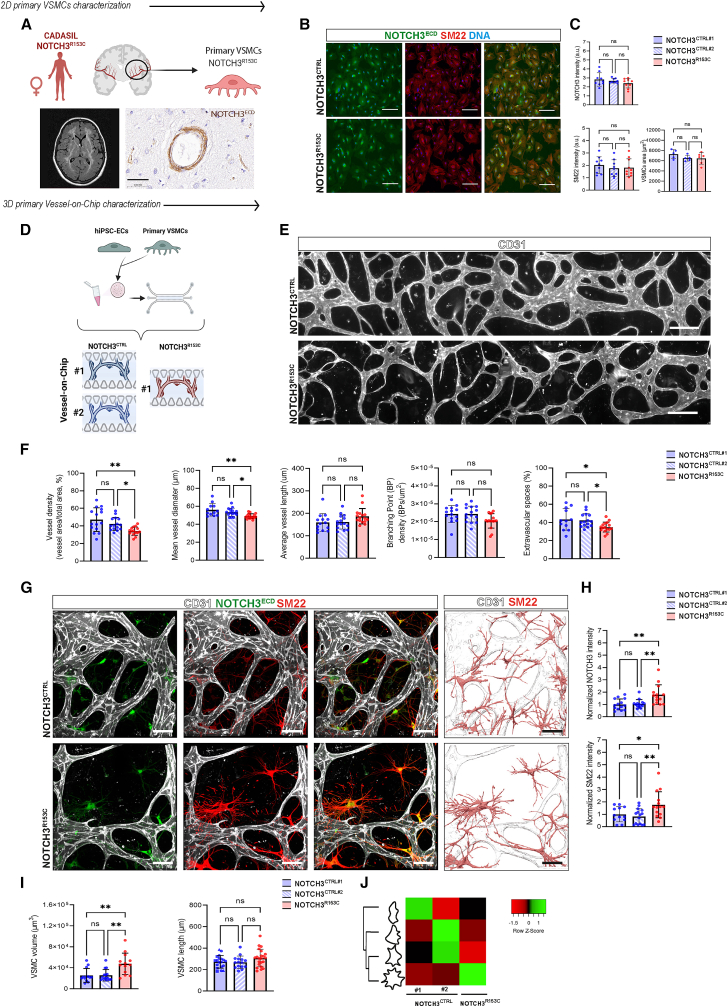
Primary CADASIL VSMCs reveal increased NOTCH3 levels and VSMC phenotypical changes in 3D Vessel-on-Chip (A) Schematic representation showing NOTCH3^R153C^ primary VSMC isolation from brain blood vessels. Brain magnetic resonance imaging (MRI) of the patient showed the typical CADASIL neuroimaging hallmarks, including confluent white matter hyperintensities and lacunes. NOTCH3 immunohistochemistry of skin vessels showed pathognomonic NOTCH3 protein accumulation. Scale bar, 50 μm. (B) Representative immunofluorescence images showing expression of NOTCH3 (green), SM22 (red), and nuclei (blue) of primary VMSCs. Magnification: 10×, scale bars, 250 μm. (C) Quantification of NOTCH3 and SM22 intensity (a.u.) and area (μm^2^) of primary VSMCs. (D) Schematic representation of 3D Vessel-on-Chip experiments with primary VMSCs. Primary VSMCs from two control patients (NOTCH3^CTRL^) and one CADASIL patient (NOTCH3^R153C^) were cultured with hiPSC-ECs in microfluidic devices. (E) Representative images of whole vascular networks formed by hiPSC-ECs (CD31, gray). Magnification: 10×, scale bars, 200 μm. (F) Quantification of vessel density (%), mean vessel diameter (μm), average vessel length (μm), branching point (BP) density (BPs/μm^2^), and extravascular spaces (%). (G) Representative confocal images of microvascular network showing hiPSC-ECs (gray; CD31) and primary VSMCs (green; NOTCH3, red; SM22) and surface-rendered images. Magnification: 40×, scale bars, 100 μm. (H) Quantification of the normalized intensities of NOTCH3 and SM22 in primary VSMCs. (I and J) Quantification of primary VSMC volume (μm^3^, I) and length (μm, I) and heatmap comparing the abundance of primary VSMCs falling into four different cell shapes (J). Data are from *N* = 3 three independent experiments and shown as ± SD. One-way ANOVA test. ^∗∗^*p* < 0.005, ^∗^*p* < 0.05, ns, not significant.

To develop an informative *in vitro* model for CADASIL, we first examined how primary VSMCs cultured from vessels isolated from postmortem brain tissue of a CADASIL patient with the *NOTCH3* c.457C>T; p.(Arg153Cys) variant (NOTCH3^R153C^) behaved in 2D versus 3D Vessel-on-Chip systems compared to primary VSMCs from two control donors (NOTCH3^CTRL1^ and NOTCH3^CTRL2^, Figure 1A). In 2D immunofluorescence analysis, NOTCH3^R153C^ VSMCs showed no significant differences in intensity of NOTCH3^ECD^ and the contractile marker SM22 (TAGLN gene product), or in surface area compared to primary control VSMCs (NOTCH3^CTRL^) (Figures 1B and 1C). Next, we integrated the primary brain VSMCs together with control hiPSC-ECs in a fibrin hydrogel to engineer 3D Vessel-on-Chip models as previously described (Figure 1D) (Vila Cuenca et al., 2021; Orlova et al., 2022). After 7 days of co-culture, 3D Vessel-on-Chip containing NOTCH3^R153C^ VSMCs showed changes in vessel morphology including significantly reduced vessel density, vessel diameter, and extravascular spaces compared to NOTCH3^CTRL^, while no differences were observed in branching point density or average vessel length (Figures 1E and 1F). In contrast to the 2D cultures, NOTCH3^R153C^ VSMCs in the 3D Vessel-on-Chip showed higher levels of NOTCH3^ECD^ and SM22 (Figures 1G and 1H). These changes were accompanied by morphological changes in NOTCH3^R153C^ VSMCs, including increased volume and stellate morphology with prevalence of protrusions, while NOTCH3^CTRL^ VSMCs largely retained a spindle-like morphology (Figures 1I and 1J). This indicated that CADASIL VSMCs exhibit increased NOTCH3^ECD^ protein levels and undergo phenotypic changes, which are linked to alterations in vessel morphology but only in the 3D Vessel-on-Chip model.

### CADASIL hiPSC-VSMCs show reduced maturation in 2D with no changes in NOTCH3^ECD^ levels

**Figure 2:**
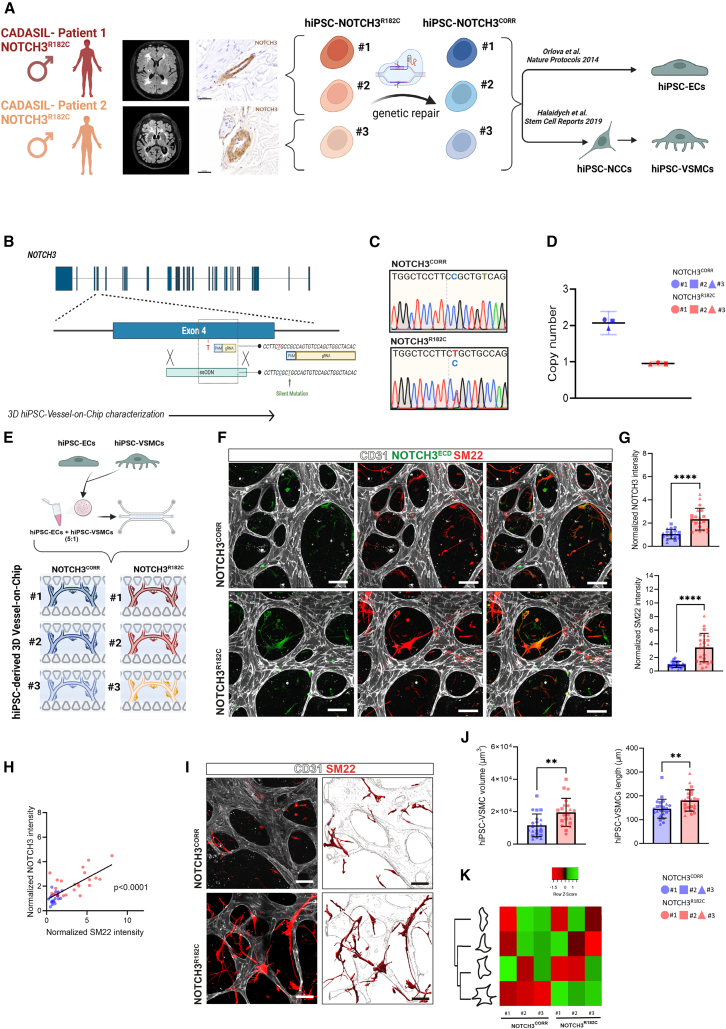
CADASIL hiPSC-VSMCs recapitulate increased NOTCH3 levels and VSMC alterations in 3D Vessel-on-Chip (A) Schematic of study design. Three clones of hiPSC-NOTCH3^R182C^ were generated from two CADASIL patient lines, and gene-corrected lines (hiPSC-NOTCH3^CORR^) were generated using CRISPR-Cas9. Brain MRI of both patients showed extensive confluent white matter hyperintensities and multiple lacunes. NOTCH3 immunohistochemistry of skin vessels showed pathognomonic NOTCH3 protein accumulation in both patients. Scale bars, 50 μm. hiPSC lines were differentiated toward ECs and VSMCs via NC intermediates using established protocols. (B) Schematic overview of the targeting strategy to correct the heterozygous c.544C>T;p.Arg182Cys pathogenic variant in the patient-derived hiPSC lines (hiPSC-NOTCH3^R182C^). Double-stranded breaks were introduced in the genome guided by guide RNAs in NOTCH3 exon 4. A repair template was used for homologous recombination (ssODN) with the corrected allele at position 544 and with a silent variant into the PAM. (C) Sanger sequencing reads of *NOTCH3* showing the heterozygous c.544C>T; p.Arg182Cys variant in the patient-derived hiPSC lines (NOTCH3^R182C^) and the correction of the variant and presence of the silent variant in the PAM in isogenic control lines (NOTCH3^CORR^). (D) Graph showing copy number variation of the corrected allele. (E) Schematic representation of 3D Vessel-on-chip experiments with hiPSC-derived vascular cells. hiPSC-VSMCs NOTCH3^R182C^ and hiPSC-VSMCs NOTCH3^CORR^ were cultured with hiPSC-ECs in microfluidic devices. (F) Representative confocal images of the microvascular network showing hiPSC-ECs (gray; CD31) and hiPSC-VSMCs (green; NOTCH3, red; SM22). Magnification: 40×, scale bars, 100 μm. (G and H) Quantification of the normalized intensities of NOTCH3 and SM22 in hiPSC-VSMCs (G) and respective Spearman correlation (H). (I) Representative confocal images of microvascular network showing in hiPSC-ECs (gray; CD31) and hiPSC-VSMCs (red; SM22). Magnification: 40×, scale bars, 100 μm. (J and K) Quantification of hiPSC-VSMC volume (μm^3^, J) and length (μm, J) and heatmap comparing the abundance of hiPSC-VSMCs falling into four different cell shapes (K). Data are from *N* = 4 four independent experiments and shown as ± SD. Unpaired *t* test. ^∗∗∗∗^*p* < 0.0001, ^∗∗^*p* < 0.005, ns, not significant.

Since primary VSMCs are difficult to obtain from postmortem patients and it is essential to whether findings are reproducible across patients, we investigated whether the same phenotypic changes were evident in hiPSC-derived VSMCs from CADASIL patients. We first generated three hiPSC clones from two CADASIL patients with a *NOTCH3* c.544C>T; p.Arg182Cys variant (NOTCH3^R182C^) (Figure 2A). We used CRISPR-Cas9 to correct the *NOTCH3* c.544C>T variant to develop line-specific isogenic controls (NOTCH3^CORR^, Figure 2B). We subcloned two hiPSC lines for Patient 1 and one subclone for Patient 2, each paired with its respective genetically repaired isogenic control. Results presented are averages of data from these three subclones. We confirmed the correction of the NOTCH3^R182C^ variant by Sanger sequencing and that both alleles were gene edited by digital droplet PCR-based allelic drop-off assay (Figures 2C and 2D). Both the CADASIL and isogenic corrected hiPSC lines showed a normal karyotype by G-banding and exhibited typical pluripotency features, such as the expression of OCT4, TRA 1–60, and NANOG, and multi-lineage differentiation potential (Figures S1A–S1C). We generated hiPSC-derived VSMCs via neural crest (NC) intermediates as previously described (Halaidych et al., 2019), as these are the primary source of VSMCs in the cerebral vasculature (Majesky 2007). We also generated hiPSC-ECs using established protocols (Orlova et al., 2014; Orlova, van den Hil et al., 2014). Unexpectedly, NOTCH3^R182C^ and NOTCH3^CORR^ hiPSCs showed different NC differentiation dynamics with NOTCH3^R182C^ hiPSC-NC cells showing reduced CD271^+^ and increased SOX2^+^ populations at initial stages (p0) of NC differentiation (Figures S2A and S2B). However, NC cell (NCC) derivation efficiencies were comparable by p3 (Figures S2A and S2B). Bulk RNA sequencing confirmed that both NOTCH3^CORR^ and NOTCH3^R182C^ hiPSC clones differentiated into hiPSC-derived ECs, NCCs, and VSMCs, with minimal variance as shown by principal-component analysis (PCA, Figure S2C). We observed that 23.1% of the total variation within the data was due to differences between both patients and continued with cells derived from Patient 1 for downstream analysis (Figure S2C). We next identified 401 differentially expressed genes between NOTCH3^CORR^ and NOTCH3^R182C^ hiPSC-VSMCs (Figure S2D; Table S1). Pathway analysis indicated enrichment of pathways related to extracellular matrix organization and degradation in hiPSC-VSMC NOTCH3^R182C^ (Figure S2E). Immunofluorescence 2D analysis showed no difference in NOTCH3^ECD^ levels between NOTCH3^R182C^ and NOTCH3^CORR^ hiPSC-VSMCs (Figures S2F and S2G). Further, 2D characterization of hiPSC-VSMC revealed a significant reduction in the contractile protein SM22 in NOTCH3^R182C^ hiPSC-VSMCs and changes in cell morphology, evidenced by a reduction in VSMC surface area compared to isogenic controls (Figures S2F and S2G).

### hiPSC-derived CADASIL VSMCs recapitulate primary CADASIL VSMC features in 3D Vessel-on-Chip

**Figure 3:**
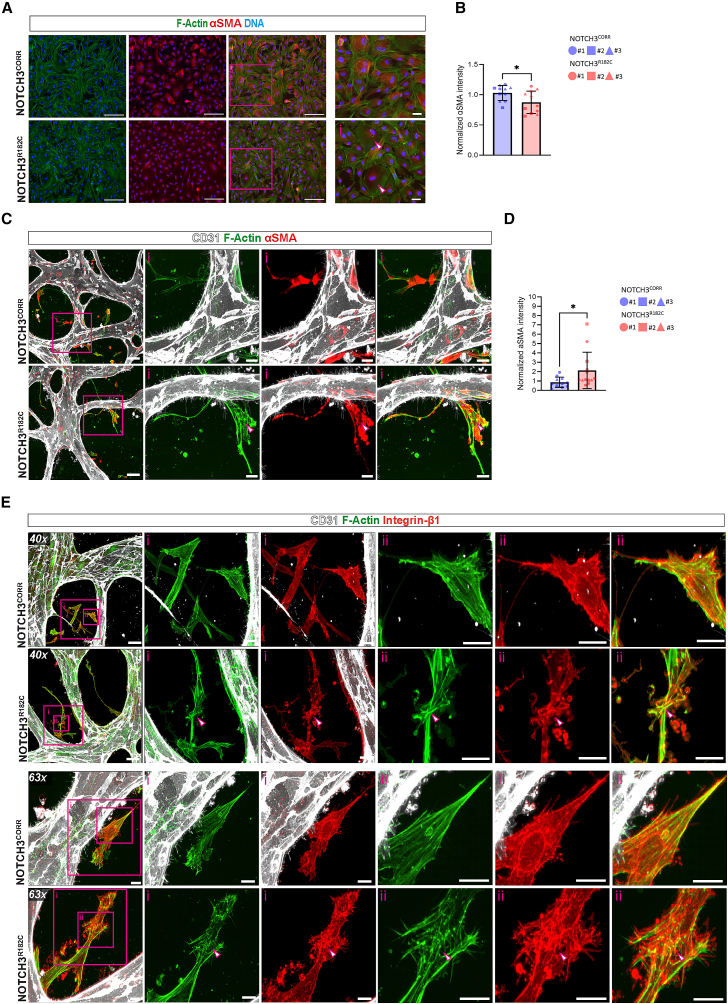
CADASIL hiPSC-VSMCs show disrupted actin cytoskeleton organization and integrin β1 focal adhesions in 3D Vessel-on-Chip (A) Representative immunofluorescence images showing expression of F-actin (green), αSMA (red), and nuclei (blue) of hiPSC-VSMCs. Magnification: 10×, scale bars, 250 μm. Enlargement (i): arrows indicate less stress F-actin fiber distribution in NOTCH3^R182C^ hiPSC-VSMCs compared to NOTCH3^CORR^ hiPSC-VSMCs, scale bars, 50 μm. (B) Quantification of intensity of normalized αSMA intensity hiPSC-VSMCs. (C) Representative confocal images of microvascular network showing hiPSC-ECs (gray; CD31) and F-actin (green) and αSMA (red). Magnification: 40×, scale bars, 100 μm. Enlargement (i): arrows indicate F-actin node organization in NOTCH3^R182C^ hiPSC-VSMCs, scale bars, 20 μm. (D) Quantification of intensity of normalized αSMA intensity in hiPSC-VSMCs. (E) Representative confocal images of microvascular network showing hiPSC-ECs (gray; CD31), F-actin (green), and integrin β1 focal adhesions (red). Magnification: 40×, scale bars, 50 μm. Enlargement (i and ii): arrows indicate disrupted patterns of F-actin and integrin β1 in NOTCH3^R182C^ hiPSC-VSMCs, scale bars, 20 μm. Magnification: 63×, scale bars, 10 μm. Enlargement (i and ii): arrows indicate disrupted patterns of F-actin and integrin β1 in NOTCH3^R182C^ hiPSC-VSMCs, scale bars, 10 μm. Data are from *N* = 3 three independent experiments and shown as ±SD. Unpaired *t* test. ^∗^*p* < 0.05, ns.

We next incorporated either NOTCH3^R182C^ or NOTCH3^CORR^ hiPSC-VSMCs along with NOTCH3^CORR^ hiPSC-ECs into the 3D Vessel-on-Chip (Figure 2E). Both types of hiPSC-VSMCs supported the formation of microvascular networks and luminized vessels in microfluidic chips after 7 days of co-culture (Figures S3A and S3B). In contrast to primary CADASIL brain VSMCs, no significant differences in microvascular network parameters were observed between the groups (Figure S3C). Immunofluorescence analysis revealed that NOTCH3^R182C^ hiPSC-VSMCs exhibited increased levels of NOTCH3 protein in the 3D Vessel-on-Chip, like CADASIL primary VSMCs (Figures 2F and 2G). Additionally, an increase in SM22 contractile protein was observed in NOTCH3^R182C^ hiPSC-VSMCs, which was correlated with the increased NOTCH3 protein levels (Figures 2F–2H). Quantification of the morphological parameters of hiPSC-VSMCs revealed striking differences in NOTCH3^R182C^ hiPSC-VSMCs compared to isogenic counterparts, like those observed with CADASIL primary VSMCs (Figure 2I; Video S1). Specifically, NOTCH3^R182C^ hiPSC-VSMCs exhibited a larger volume and a greater number of protrusions, as indicated by more stellate-shaped cells (Figures 2I–2K), while NOTCH3^CORR^ hiPSC-VSMCs displayed a more spindle-like morphology (Figure 2K). In 2D cultures, NOTCH3^R182C^ hiPSC-VSMCs exhibited fewer and less-prominent actin stress fibers, together with significantly lower intensity of other contractile proteins such as αSMA (ACTA2 gene product; Figures 3A and 3B). In contrast, when incorporated in the 3D Vessel-on-Chip, NOTCH3^R182C^ hiPSC-VSMCs displayed an abnormal cytoskeletal architecture characterized by highly dense actin bundles within the VSMC protrusions and disrupted patterns forming node-like structures (Figure 3C). Consistent with changes in SM22, αSMA protein levels were also significantly increased in the 3D Vessel-on-Chip (Figures 3C and 3D). In addition, we observed altered patterns in integrin β1-associated focal adhesion in NOTCH3^R182C^ hiPSC-VSMCs, which appeared abnormally clustered within the disrupted node-like actin structures, whereas NOTCH3^CORR^ hiPSC-VSMCs showed focal adhesions predominantly localized at cellular extensions (Figure 3E). This suggested that the increase of NOTCH3 in CADASIL is related to phenotypic changes in hiPSC-VSMCs, mirroring those observed in primary brain VSMCs from CADASIL patients cultured in the 3D Vessel-on-Chip.

Video S1. 3D surface rendering of 3D Vessel-on-Chip

### PDGFRβ protein levels correlate with NOTCH3^ECD^ levels in CADASIL 3D Vessel-on-Chip and patient tissue

**Figure 4:**
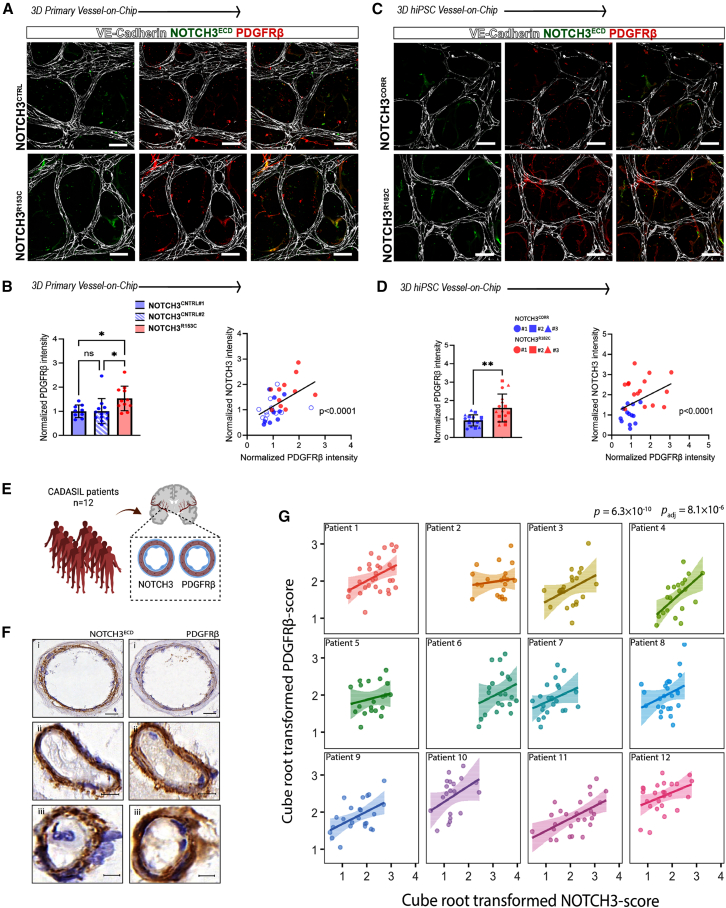
CADASIL VSMCs show increased PDGFRβ protein levels correlating with NOTCH3 protein levels in 3D Vessel-on-Chip and in patient brain tissue (A) Representative confocal images of microvascular network showing hiPSC-ECs (gray; CD31) and primary VSMCs (green; NOTCH3, red; PDGFRβ). Magnification: 40×, scale bars, 100 μm. (B) Quantification of normalized PDGFRβ intensity and Spearman correlation between normalized NOTCH3 and PDGFRβ intensities in primary VSMCs. (C) Representative confocal images of microvascular network showing hiPSC-ECs (gray; CD31) and hiPSC-VSMCs (green; NOTCH3, red; PDGFRβ). Magnification: 40×, scale bars 100 μm. (D) Quantification of normalized PDGFRβ intensity and Spearman correlation between normalized NOTCH3 and PDGFRβ intensities in hiPSC-VSMCs. (E) Schematic representation of CADASIL patient immunohistochemistry. (F) Representative brain blood vessels from CADASIL patients with different sizes showing NOTCH3 aggregates and PDGFRβ protein in the blood vessel wall. Scale bars, 20 (i), 10 (ii), and 5 (iii) μm. (G) Correlation analysis between cube root-transformed PDGFRβ and NOTCH3 score from CADASIL patients (*n* = 12). Each plot represents one individual, and each dot a blood vessel measurement. Data are from *N* = 3 three independent experiments and shown as ± SD. One-way ANOVA test (B) and *t* test (D). ^∗∗^*p* < 0.005, ^∗^*p* < 0.05, ns, not significant.

PDGFRβ is a direct downstream target of NOTCH3 (Jin et al., 2008), and the interaction between NOTCH3 and PDGFRβ plays a critical role in maintaining VSMC function and vascular homeostasis by regulating pathways involved in cell proliferation, migration, survival, and differentiation (Domenga et al., 2004). Immunofluorescence analysis in the 3D Vessel-on-Chip showed an increase in PDGFRβ intensity in both NOTCH3^R153C^ primary VSMCs and NOTCH3^R182C^ hiPSC-VSMCs compared to their respective controls (Figures 4A–4C). Additionally, PDGFRβ levels were positively correlated with NOTCH3^ECD^ protein levels (Figure 4D). We next investigated whether the observed changes in protein levels were attributable to increased mRNA levels of NOTCH3 and its downstream target genes. Using a Jagged1-bead activation assay in 2D hiPSC-VSMCs (Figure S4A) (Zohorsky et al. 2021), we showed that the NOTCH3^R182C^ variant did not significantly alter the expression of *NOTCH3* or its downstream target genes (Figure S4B). Similarly, in the 3D Vessel-on-Chip model, we found no significant differences in mRNA levels of *NOTCH3* or *PDGFRβ* or other *NOTCH3* downstream target genes (Figure S4C), or in the EC-specific gene *PECAM1* or the VSMC-specific gene TAGLN (Figure S4C). We next investigated the relationship between NOTCH3 and PDGFRβ protein levels in brain blood vessels from CADASIL patients (*n* = 12; Table S2), by immunohistochemical staining of brain blood vessels from CADASIL patients and controls, as previously described (Figure 4E) (Gravesteijn et al., 2022; Rutten et al., 2015). We found a significant positive correlation in each patient between NOTCH3 and PDGFRβ protein levels in CADASIL patient vessels (*n* = 12) (Figures 4F and 4G). This indicated correlation between PDGFRβ and NOTCH3^ECD^ levels in the 3D Vessel-on-Chip model, which reflect that in patient brain tissue.

### CADASIL hiPSC-derived VSMCs are functionally deficient in 3D Vessel-on-Chip

**Figure 5:**
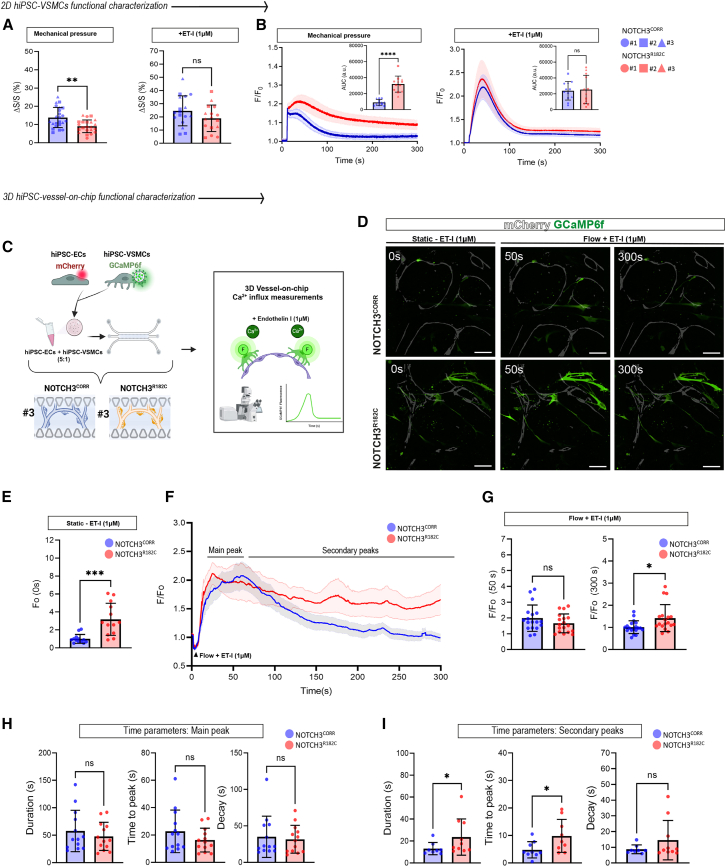
CADASIL hiPSC-VSMCs show altered Ca^2+^ dynamics in 3D Vessel-on-Chip (A and B) 2D functional characterization of hiPSC-VSMCs. Relative cell surface area decrease upon mechanical stimulation (medium refreshment) and ET-I stimulation (A). Normalized average fluorescence intensity F/F_0_ in hiPSC-VSMCs upon mechanical stimulation (medium refreshment) and ET-I stimulation (B). Inserts show quantifications of the AUC. (C) Schematic of the functional 3D Vessel-on-Chip study using hiPSC-VSMCs engineered to express an ultra-sensitive Ca^2+^ sensor (GCaMP6f) cultured with fluorescently tagged (mCherry) healthy control hiPSC-ECs. (D) Representative confocal images of intracellular Ca^2+^ fluorescence showing hiPSC-ECs (gray; mCherry) and hiPSC-VSMCs (green; GCaMP6f) at different time points (0, 50, and 300 s) after ET-I stimulation. Magnification: 20×, scale bars, 100 μm. (E) Fluorescence intensity at time (F_0_, 0 s) of hiPSC-VSMCs cultured in 3D Vessel-on-chip under static conditions. (F) Normalized average fluorescence intensity F/F_0_ in hiPSC-VSMCs expressing GCaMP6f. Stimulation time point is set at 5 s. (G–I) Ca^2+^ transient parameters: average fluorescence intensity F/F_0_ 50 s and 300 s (G). Duration (s), time to peak (s), and decay (s) of the MP (H) and SP (I). Data are from *N* = 3 three independent experiments and shown as ±SD. Unpaired *t* test. ^∗∗∗∗^*p* < 0.0001,^∗∗∗^*p* < 0.001, ^∗∗^*p* < 0.01, ^∗^*p* < 0.05, ns, not significant.

We next explored whether NOTCH3^R182C^ affects the functional properties of VSMCs. In 2D functional assays, NOTCH3^R182C^ hiPSC-VSMCs exhibited reduced contractility and prolonged Ca^2+^ waves in response to mechanical pressure, while stimulation with endothelin-1 (ET-I) induced similar responses in NOTCH3^R182C^ and NOTCH3^CORR^ hiPSC-VSMCs (Figures 5A and 5B). To assess functional changes in the 3D Vessel-on-Chip model, we engineered NOTCH3^R182C^ and NOTCH3^CORR^ hiPSC-VSMCs from Patient 2 such that they expressed an ultra-sensitive Ca^2+^ sensor (GCaMP6f) (Chen et al., 2013) as previously described (Vila Cuenca et al., 2021). Next, we determined the cytosolic Ca^2+^ release in the 3D Vessel-on-Chip on day 7 (Figure 5C; Video S2). Before ET-I perfusion (time 0 s), NOTCH3^R182C^ hiPSC-VSMCs showed significantly higher basal fluorescence intensity compared to NOTCH3^CORR^ hiPSC-VSMCs (Figures 5D and 5E). At 50 s after ET-I stimulation, fluorescence intensity was comparable between both groups, indicating that NOTCH3^R182C^ and NOTCH3^CORR^ hiPSC-VSMCs showed a similar response to ET-I stimulation during the main peak ([MP] Figures 5F and 5G). At 300 s, the fluorescence intensity of NOTCH3^R182C^ hiPSC-VSMCs remained elevated, while that of NOTCH3^CORR^ hiPSC-VSMCs had returned to baseline, reflecting differences in the secondary peak (SP) response (Figures 5F and 5G). Comparison of kinetics of the Ca^2+^ responses showed no differences between the groups in the time parameters during the MP (Figure 5H), whereas NOTCH3^R182C^ hiPSC-VSMCs displayed an increased duration and time to peak but no changes in the decay during the SP response (Figure 5I). Together, these data demonstrate that NOTCH3^R182C^ hiPSC-VSMCs exhibit abnormal Ca^2+^ responses in the 3D Vessel-on-Chip model, validating its utility to uncover functional differences between normal and CADASIL hiPSC-VSMCs.

Video S2. Ca^+2^ dynamics of hiPSC-VSMCs in 3D Vessel-on-Chip

### Inhibition of NOTCH3 cleavage rescues CADASIL hiPSC-VSMC phenotypic and functional changes in 3D Vessel-on-Chip model

**Figure 6:**
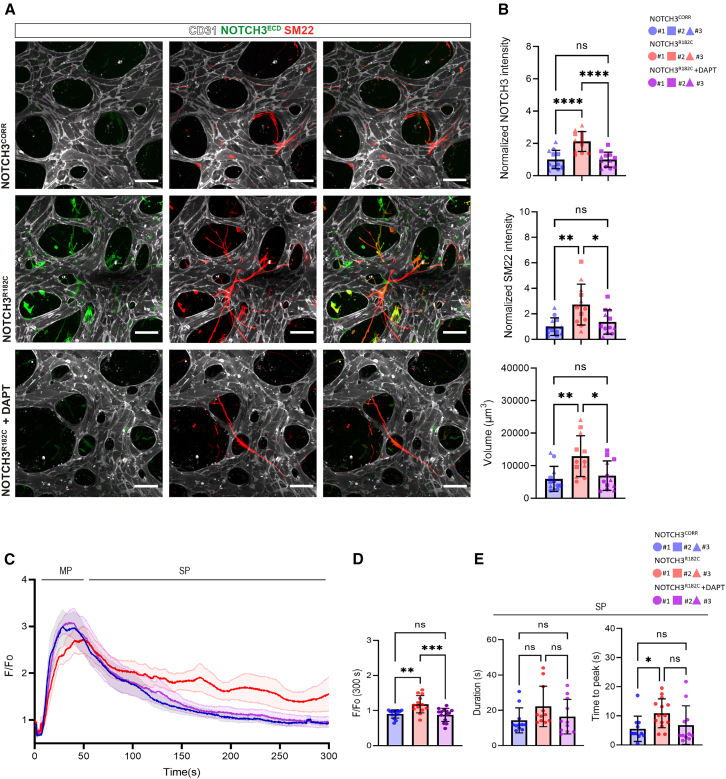
Inhibition of NOTCH3 cleavage rescues VSMC phenotypic and functional changes in hiPSC-derived 3D Vessel-on-Chip (A) Representative confocal images of microvascular network showing in hiPSC-ECs (gray; CD31) with hiPSC-VSMCs (NOTCH3, green; SM22, red). Magnification: 40×, scale bars, 100 μm. (B) Quantification of normalized intensity of NOTCH3, SM22, and volume (μm^3^) in hiPSC-VSMCs. (C) Normalized average fluorescence intensity F/F_0_ in hiPSC-VSMCs expressing GCaMP6f. Stimulation time point is set as t = 5 (s). (D and E) Ca^2+^ transient parameters: Average fluorescence intensity F/F_0_ at time 300 s (D). Duration (s) and time to peak (s) and decay (s) of the SP (E). Data are from *N* = 3 three independent experiments and shown as ± SD. One-way ANOVA test. ^∗∗∗∗^*p* < 0.0001, ^∗∗∗^*p* < 0.001, ^∗∗^*p* < 0.005, ^∗^*p* < 0.05, ns, not significant.

The γ-secretase inhibitor DAPT has been shown to block the *in vitro* and *in vivo* cleavage and signaling of NOTCH proteins (Li et al., 2009; Hellstrom et al., 2007). We, therefore, hypothesized that inhibiting γ-secretase could mitigate the effects of pathogenic *NOTCH3* variants in VSMCs within the 3D Vessel-on-Chip model. After 48 h of DAPT treatment, NOTCH3^R182C^ hiPSC-VSMCs showed reduced NOTCH3^ECD^ and SM22 protein levels, as well as a reduction in cell volume, resembling their untreated NOTCH3^CORR^ hiPSC-VSMCs counterparts (Figures 6A and 6B). Additionally, DAPT treatment normalized Ca^2+^ responses in NOTCH3^R182C^ hiPSC-VSMCs (Figure 6C), particularly at 300 s after ET-I stimulation, as well as the response kinetics of the SP response in NOTCH3^R182C^ hiPSC-VSMCs (Figures 6D and 6E). These results indicate that lowering NOTCH3^ECD^ levels by γ-secretase inhibition restores the morphological and functional changes in CADASIL hiPSC-VSMCs.

## Discussion

In this study, we developed a multi-cell type CADASIL 3D Vessel-on-Chip model that has disease-relevant morphological and functional readouts in VSMCs. The results from the model were similar to those obtained using patient-derived primary VSMCs, hiPSC-derived VSMCs, and their isogenic corrected controls, providing a robust *in vitro* platform for studying early pathological changes in VSMCs and testing of therapeutic compounds.

CADASIL-causing *NOTCH3* variants lead to aggregation and accumulation of NOTCH3^ECD^ around VSMCs with sequestration of extracellular matrix proteins, resulting in VSMC degeneration and vessel wall dysfunction (Joutel et al., 2000). We found increased NOTCH3^ECD^ protein in both CADASIL primary and hiPSC-derived VSMCs after 7 days of co-culture in the 3D Vessel-on-Chip model. Notably, this was not observed in conventional 2D assays, highlighting the importance of the 3D environment for recapitulating the interaction between ECs and VSMC. A similar conclusion was drawn in a previous study on the microvascular disease hereditary hemorrhagic telangiectasia (Orlova et al., 2022).

NOTCH3 regulates the expression of contractile VSMC markers and is critical for the differentiation and maintenance of the contractile VSMC phenotype (Henshall et al., 2015). We found that both primary and hiPSC-derived CADASIL VSMCs exhibited contractile-like morphologic changes in a 3D context, including elongated morphology, larger size, and more protrusions (Liu et al. 2009; Henshall et al., 2015). These alterations were accompanied by increased levels of the contractile proteins SM22 and αSMA, as well as disrupted actin cytoskeleton organization and altered integrin β1-associated focal adhesions. Such abnormalities in actin cytoskeleton organization and focal adhesion structure have also been reported in previous studies using primary and hiPSC-derived CADASIL VSMCs (Tikka et al., 2012; Ling et al., 2019), supporting the notion that cytoskeletal and adhesion defects are consistent and characteristic features of CADASIL VSMCs. Here, we observed a strong correlation between NOTCH3^ECD^ and SM22 levels in CADASIL VSMCs, suggesting that pathogenic *NOTCH3* variants drive the acquisition of a contractile phenotype in these cells (van Splunder et al., 2024; Gatti et al., 2018). This aligns with previous findings that NOTCH3 is essential for maintaining vascular contractility, as *Notch3*-deficient mice exhibit cerebrovascular dysfunction due to the loss of VSMC contractility (Romay et al., 2024; Domenga et al., 2004). In the present study, we also observed a strong correlation between PDGFRβ and NOTCH3^ECD^ protein levels in the 3D Vessel-on-Chip model, a relationship further confirmed in brain blood vessels from CADASIL patients. The role of PDGFRβ in CADASIL has been inconclusive, with contradictory results from studies using 2D models (Tikka et al., 2012; Kelleher et al., 2019; Jin et al., 2008). An increase in PDGFRβ levels in CADASIL patient brain arteries has been previously found (Craggs et al., 2015; Littau et al., 2022), although a direct relationship with NOTCH3^ECD^ aggregates has not been described. Moreover, studies in mice have shown that constitutive activation of PDGFRβ increases mural cell coverage, promotes a pro-inflammatory profile, and enhances the synthesis of extracellular matrix proteins, providing an additional link between PDGFRβ and vessel wall pathology (Olson and Soriano 2011; He et al., 2015).

Recent studies with hiPSC-derived CADASIL vascular cells showed that VSMCs fail to stabilize tubular structures in a Matrigel cord-forming assay (Kelleher et al., 2019). In this study, vascular defects, including a reduction of vessel diameter were only induced by primary CADASIL VSMCs in the 3D Vessel-on-Chip model. This discrepancy between primary and hiPSC-derived VSMCs may be attributable to differences in cellular maturity and/or overall behavior when VSMCs are cultured in the 3D Vessel-on-Chip, as we previously showed (Vila Cuenca et al., 2021; Halaidych et al., 2019).

NOTCH3 autoregulates its own expression through a positive feedback loop and modulates downstream target genes upon contact with ECs (Liu et al. 2009). Although a long-standing debate has been whether increased or reduced NOTCH3 signaling contributes to CADASIL vascular pathology, recent work in Tg*Notch3*^*R169C*^ mice arteries showed no changes in mRNA expression of Notch3 downstream target genes (Dupre et al., 2024). Consistent with this, we recently demonstrated that although different NOTCH3 risk categories are strongly associated with distinct levels of vascular NOTCH3^ECD^ aggregation load in patient brain and skin vessels, NOTCH3 signaling activity itself does not differ across those categories (Hack et al., 2023). In the present study, we similarly observed no substantial changes in mRNA levels of *NOTCH3*, contractile proteins, or NOTCH3 downstream targets such as *HEY1*, *HES1*, and *PDGFRB* in either the 2D Jagged1-bead activation assay or the 3D Vessel-on-Chip model. Together, these findings further support the now widely accepted hypothesis that CADASIL pathology is primarily driven by the accumulation of NOTCH3 protein.

NOTCH3 is a key regulator of vascular tone in small arteries and of vascular reactivity in response to mechanical factors such as pressure and flow (Belin de Chantemele et al., 2008; Hussain et al., 2004). Notably, *Notch3* deficiency in mice has been associated with the disruption of proteins involved in Ca^2+^ dynamics (Romay et al., 2024), a key factor in vascular smooth muscle contraction, which is triggered by an increase in cytosolic free Ca^2+^ concentration (Touyz et al., 2018). In the present study, CADASIL hiPSC-VSMCs exhibited abnormal contractile behavior and disrupted Ca^2+^ dynamics in 2D cultures when subjected to mechanical pressure, but not in response to the vasoactive compound ET-I. In contrast, functional assays using the 3D Vessel-on-Chip model revealed prolonged Ca^2+^ responses in CADASIL hiPSC-VSMCs upon flow perfusion with ET-I-supplemented medium. The observed phenotypic discrepancies, together with differences in contractile protein expression, may reflect distinct maturation states of hiPSC-VSMCs in 2D and 3D culture conditions. The 3D Vessel-on-Chip system allows direct contact with hiPSC-ECs, which may promote a more mature and contractile VSMC phenotype and facilitate the emergence of CADASIL-relevant phenotypes (Cerneckis et al. 2024). Together, these findings indicate that CADASIL VSMCs exhibit a hyperactivated contractile state.

Proteolytic cleavage of NOTCH receptors is mediated by γ-secretase protein complexes, and its inhibition been shown to effectively prevent NOTCH3 S3 cleavage (Li et al., 2009; Wimmer et al., 2019). In CADASIL, ligand binding followed by NOTCH3 receptor cleavage has been proposed as a critical event driving NOTCH3^ECD^ accumulation at the VSMC surface. In our study, γ-secretase inhibition alleviated CADASIL VSMC phenotypic and functional alterations, suggesting that NOTCH3 cleavage has a restorative effect on CADASIL VSMC phenotype. We acknowledge, however, that we cannot fully exclude potential effects on NOTCH3 intracellular domain release or broader signaling alterations, even though our data show that NOTCH3^R182C^ variant does not significantly affect downstream target gene expression. Additionally, γ-secretase mediates proteolysis of other transmembrane proteins beyond NOTCH receptors (Ni et al., 2001). Therefore, although we observe a reduction of NOTCH3^ECD^ protein levels in CADASIL VSMCs upon γ-secretase inhibition, we cannot fully attribute the observed phenotypic changes solely to NOTCH3 cleavage inhibition.

Several questions remain unanswered and limit the extent of our study’s conclusions. Although we observed an increase of NOTCH3^ECD^ protein abundance in VSMCs in 3D Vessel-on-Chip, we did not observe NOTCH3^ECD^ aggregation, a hallmark of CADASIL vessel pathology. The 3D Vessel-on-Chip model employed here does not fully recapitulate the complex cellular composition of the cerebral vasculature, which might be critical for the formation of NOTCH3^ECD^ aggregates. A more complex model incorporating neuronal cell types, extending cell culture conditions, and/or introducing a constant flow could potentially address this issue, but lies beyond the scope of the current study. Finally, while blocking NOTCH3 proteolytic cleavage using the γ-secretase inhibitor DAPT rescued the phenotype in CADASIL VSMC, the broad substrate specificity of γ-secretase limits its potential clinical application.

In summary, we generated a robust CADASIL 3D Vessel-on-Chip model that shows reproducible results for both primary and hiPSC-derived CADASIL patient VSMCs including (1) increased abundance of NOTCH3^ECD^ protein; (2) morphological alterations accompanied by disrupted actin organization, abnormal focal adhesions, and increased expression of contractile and PDGFRβ proteins; as well as (3) functional alterations in calcium dynamics. Finally, we demonstrate the utility of the model for drug testing with a proof-of-concept rescue using the γ-secretase inhibitor DAPT.

## Methods

### hiPSC lines

Research on hiPSC was approved by the Medical Ethical Committee (P13.080) at Leiden University Medical Center, the Netherlands, and written informed consent was obtained from all patients. Erythroblasts isolated from peripheral blood were used for reprogramming as described previously (Bouma et al. 2017, 2020). The following hiPSC lines were generated from Patient 1: LUMC0169iNOTCH and Patient 2: LUMC0194iNOTCH. The heterozygous *NOTCH3* c.544C>T variant located in exon 4 was corrected by insertion of a single base (C) and simultaneous introduction of two silent variants using CRISPR-Cas9-induced homology-directed repair. The targeting strategy is depicted in Figure 2A. Detailed description of CRISPR gene correction strategy and hiPSC line maintenance and characterization can be found in the supplemental information.

### Setting up 3D Vessel-on-Chip culture

hiPSC differentiation toward ECs and VSMCs and 2D characterization is described in the supplemental information. Cell preparation and chip setting up was performed as described previously (Vila Cuenca et al., 2021). Commercially available microfluidic chips with one gel channel and two media channels (AIM Biotech, IdenTx 9) were used. Cells were resuspended and combined to obtain 10 × 10^6^ hiPSC-ECs/mL and 2 × 10^6^ VSMCs/mL (5:1 ratio). Cells were resuspended in EGM-2 supplemented with Thrombin (4 U/mL) and then gently mixed with fibrinogen (final concentration 3 mg/mL, Sigma) at 1:1 vol ratio. Cell/hydrogel mixture was quickly loaded into the middle gel-loading channel of the microfluidic chip. Chips were incubated at room temperature for 15 min before the addition of EGM-2 supplemented with VEGF (50 ng/mL) to both flanking media channels. The γ-secretase inhibitor DAPT (10 μM) was also added to the medium on day 1 for 24 h. Gravity-driven flow was induced by the addition of 100 μL medium to the right media ports and 50 μL media to left media ports. Medium was refreshed daily.

### CADASIL patient brain tissue staining and quantifications

In all CADASIL patients (*n* = 12, Table S2), tissue samples from frontal cortex including subcortical white matter were collected and paraffin embedded. All donors gave written informed consent, and the procedures were carried out in accordance with the Declaration of Helsinki (P18.164). Five-μm sections were pretreated with 0.1% trypsin for 30 min at 37°C for the NOTCH3 staining, and with 0.1M citrate buffer for 10 min for the PDGFRβ staining, and washed with PBS three times. The slides were incubated at room temperature for 2 h with a primary mouse anti-NOTCH3 antibody (clone 1E4, Millipore) or for 1 h with a primary goat anti-PDGFRβ (AF385, R&D Systems). A two-step detection system (BrightVision, ImmunoLogic, VWRKC-DPVB55HRP) was used per the manufacturer’s protocol. In short, slides were post-blocked for 15 min, washed with PBS, incubated for 30 min with an anti-mouse HRP antibody or anti-goat probe/HRP (GHP516), and washed in PBS and subsequently stained with 3,3′-diaminobenzidine (DAB) + Substrate Chromogen System (Dako, K3468, diluted 1:50). Counterstaining was then performed with 1:10 diluted Harris hematoxylin for 10 s. To assess the correlation of NOTCH3 and PDGFRβ stainings, for each brain, 20 blood vessels were identified that were present on both NOTCH3- and PDGFRβ-stained slides. The vessel walls were then manually segmented to create a region of interest (ROI) using QuPath. Within these ROIs, NOTCH3 aggregation load and PDGFRβ staining was quantified by using Color Threshold in ImageJ (settings: Hue, 40–210 [stop]; saturation, 0–255; brightness, 0–150). The NOTCH3 and PDGFRb scores per blood vessel were equal to the fraction of surface wall area positive for these respective stainings.

### Statistical analysis

Statistical analyses were performed using GraphPad Prism 9 software. Normality of the data was evaluated by the D’Agostino-Pearson test. One- and two-way ANOVA with Tukey’s multiple comparison test was used for the analysis of three groups. For paired or unpaired analysis of two groups, either Student’s *t* test or Wilcoxon-Mann-Whitney test was used. Analyses are indicated in the figure legends. The data are reported as mean ± SD. Statistical significance was defined as *p* < 0.05. For analyzing the correlation between NOTCH3 and PDGFRβ staining, a linear mixed model with a random intercept per patient ID was created with the PDGFRb score as a dependent variable and the NOTCH3 score as an independent variable. To obtain normal distribution and homoscedasticity of residuals, both the NOTCH3 score and the PDGFRβ score were cube root transformed. Two-sided *p* values <0.05 were considered significant. Statistical analyses were performed in R v.4.4.0.

## Resource availability

### Lead contact

Further information and requests for resources and reagents should be directed to and will be fulfilled by the lead contact, Dr. Valeria V. Orlova (v.orlova@lumc.nl).

### Materials availability

hiPSC lines are available upon MTA.

### Data and code availability

The bulk RNA sequencing datasets have been deposited in the European Genome-Phenome Archive with accession number EGAD50000002181.

This paper does not report original code.

Software used to analyze the data is either freely or commercially available. Any additional information associated with the data presented in this paper is available from the lead contact upon request.

## Acknowledgments

We thank the LUMC human iPSC Hotel for the generation and characterization of hiPSC lines and the LUMC confocal imaging facility (Lennard Voortman and Annelies Boonzaier-van der Laan) for help with imaging. Illustrations were created using BioRender.com. We thank Tessa de Korte and Ncardia for the use of the FDSS/μcell for the Ca^2+^ experiments. We would like to thank Veronica Ramovs for her helpful discussions on focal adhesions. This work was supported by the Netherlands Organization for Health Research and Development (ZonMw; VIDI 91717325); the cureCADASIL association and 10.13039/100018232Orphan Disease Center (MDBR-22-126-CADASIL); 10.13039/501100010969Alzheimer Nederland (WE.03-2024-10); the Netherlands Organ-on-Chip Initiative, which is an 10.13039/501100003246NWO Gravitation project (024.003.001) funded by the Ministry of Education, Culture and Science of the government of the Netherlands; and the 10.13039/501100009708Novo Nordisk Foundation Center for Stem Cell Medicine, which is supported by a Novo Nordisk Foundation grant (NNF21CC0073729).

## Author contributions

Conceptualization, M.V.C., J.W.R., S.A.J.L.O., and V.V.O.; methodology, M.V.C., T.T., M.N.C., J.L.G., J.W.R., S.A.J.L.O., and V.V.O.; formal analysis, M.V.C., T.T., M.N.C., and J.L.G.; software, M.V.C., M.N.C., and J.L.G.; investigation, M.V.C., T.T., M.N.C., J.L.G., F.E.v.d.H., K.L.D., G.G., and C.F. ; resources, M.V.C., A.A.F.d.V., C.L.M., J.W.R., S.A.J.L.O., and V.V.O.; writing – original draft, M.V.C., J.W.R., S.A.J.L.O., and V.V.O.; writing – review and editing, M.V.C., C.L.M., J.W.R., S.A.J.L.O., and V.V.O.; supervision, M.V.C, J.W.R., S.A.J.L.O., and V.V.O.; project administration, M.V.C, J.W.R., S.A.J.L.O., and V.V.O.; funding acquisition, M.V.C, C.L.M., J.W.R., S.A.J.L.O., and V.V.O.

## Declaration of interests

The authors declare no competing interests.

## Supplemental information

Document S1. Figures S1–S4 and supplemental methods

Table S1. DEG genes of hiPSC-VSMCs and gene ontology enrichment

Table S2. CADASIL patient information

Document S2. Article plus supplemental information
