# Supplementary material for: 3D Vessels-on-Chip using isogenic hiPSC-derived VSMCs reveal NOTCH3-driven alterations in brain small vessel disease
Source: Stem Cell Reports. 2026 Mar 26;21(4):102863. doi: 10.1016/j.stemcr.2026.102863 (PMC13083807; doi:10.1016/j.stemcr.2026.102863)
Supplement: Document S1. Figures S1–S4 and supplemental methods [file mmc1.pdf]

Stem Cell Reports, Volume *21*

Supplemental Information

3D Vessels-on-Chip using isogenic hiPSC-derived VSMCs reveal NOTCH3-driven alterations in brain small vessel disease

Marc Vila Cuenca, Theano Tsikari, Minne N. Cerfontaine, James L. Gallant, Francijna E. van den Hil, Marga J. Bouma, Kyra L. Dijkstra, Gido Gravesteijn, Antoine A.F. de Vries, Christine L. Mummery, Julie W. Rutten, Saskia A.J. Lesnik Oberstein, and Valeria V. Orlova

INVENTORY OF SUPPLEMENTARY MATERIAL

Supplemental Figures and Legends:
Figure S1. Characterization of hiPSC lines
Figure S2. Characterization of hiPSC-derived cells
Figure S3. Vessel characterization of hiPSC-derived 3D Vessel-on-Chip
Figure S4. Expression profile of hiPSC-derived 3D Vessel-on-Chip

Supplemental Tables
Table S1. DEG genes of hiPSC-VSMCs and gene ontology enrichment
Table S2. CADASIL patient information

Supplemental Videos
Video S1. 3D surface rendering of 3D Vessel-on-Chip
Video S2. Ca+2 dynamics of hiPSC-VSMCs in 3D Vessel-on-Chip

SUPPLEMENTARY FIGURES AND LEGENDS

FIGURE S1

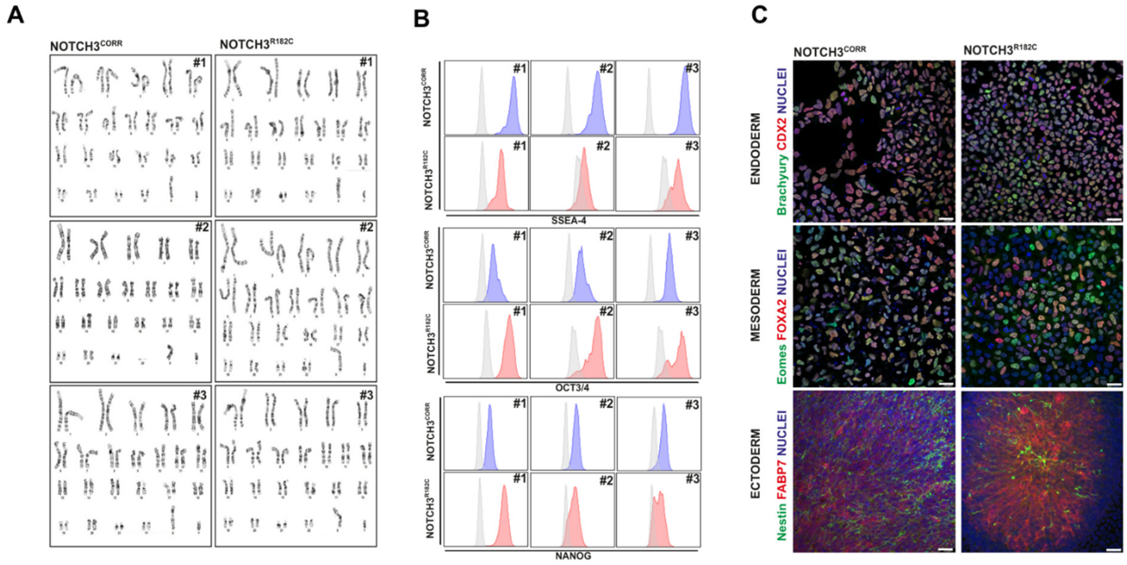

Figure S1. Characterization of hiPSC lines

(A) Karyotype of hiPSC confirmed by G-banding. (B) Expression of undifferentiated state markers SSEA4, OCT3/4 and NANOG using flow cytometry in hiPSC lines (C) Representative immunofluorescence images for markers of the three germ layers; Brachyury and CDX2 (mesoderm), FOXA2 and Eomes (endoderm) and NESTIN and FABP7 (ectoderm). Scale bar 25 µm.

FIGURE S2

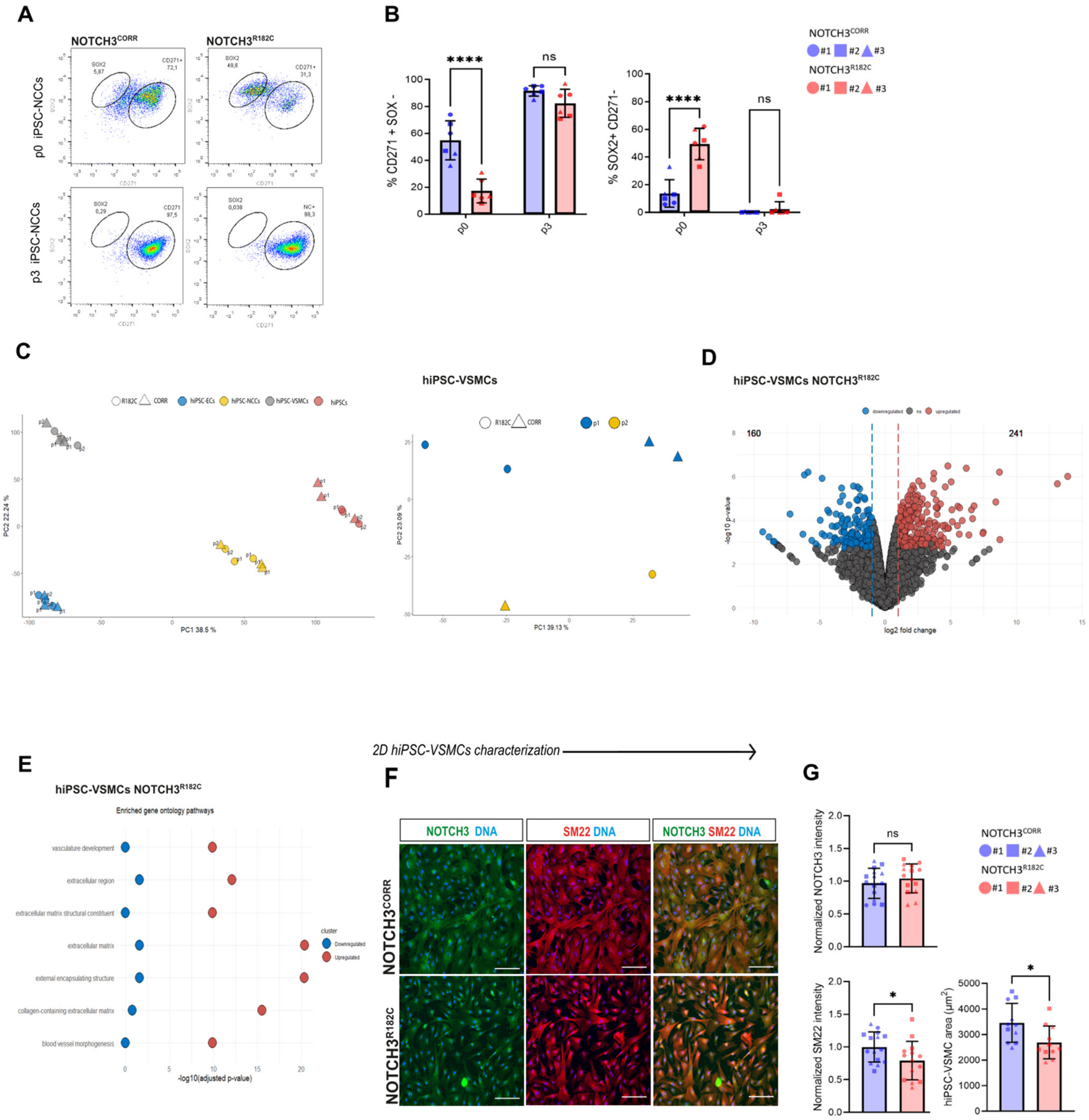
A
NOTCH3CORR
NOTCH3R182C
p0 iPSC-NCCs
p3 iPSC-NCCs
B
% CD271 + SOX -
% SOX2+ CD271-
****
ns
p0
p3
NOTCH3CORR
#1 #2 #3
NOTCH3R182C
#1 #2 #3
C
hiPSC-VSMCs
PC1 38.5 %
PC1 39.13 %
D
hiPSC-VSMCs NOTCH3R182C
160
241
log2 fold change
E
hiPSC-VSMCs NOTCH3R182C
Enriched gene ontology pathways
vasculature development
extracellular region
extracellular matrix structural constituent
extracellular matrix
external encapsulating structure
collagen-containing extracellular matrix
blood vessel morphogenesis
cluster
Downregulated
Upregulated
-log10(adjusted p-value)
2D hiPSC-VSMCs characterization
F
NOTCH3 DNA
SM22 DNA
NOTCH3 SM22 DNA
NOTCH3CORR
NOTCH3R182C
G
Normalized NOTCH3 intensity
Normalized SM22 intensity
hiPSC-VSMC area (µm2)
ns
*

Figure S2. Characterization of hiPSC-derived cells

(A) Representative FACS plots showing CD271 and SOX2 expression in hiPSC-NCCs at passage 0 and 3. (B) Quantification of percentage of positive population of hiPSCs (SOX2+ CD271-) and hiPSC-NCCs (SOX2- and CD271+) at passage 0 and 3 determined by FACS. Data are from N=3 three independent experiments and shown as ± SD. Two-way ANOVA test. ****$p < 0.0001$; ns, not significant. (C) Principal component (PC) analysis depicting the variation in the total gene expression by bulk RNA-seq of hiPSCs, hiPSC-ECs, hiPSC-NCCs and hiPSC-VSMCs. Color represents different cell types. The PCA is derived from log counts per million with batch effect correction applied to the replicates using limma. PC analysis of hiPSC-VSMCs comparing the two patients variance revealed a separation on the second component where 23.1 % of the total variation within the data is due to the difference in the two patients. For further processing of the data, we opted to continue with the cells obtained from patient 1 for downstream analysis. (D) Volcano plot displaying sorted log2 fold-change (FC) showing expression of genes in NOTCH3^{R182C} hiPSC-VSMCs (patient 1) based on the RNA-seq profiles. Log2FC < -1 indicates downregulated genes whereas log2FC > 1 indicates upregulated genes, genes were further considered significant when the q-value < 0.05 and log counts per million > 0. (E) Select gene ontology (GO) terms enriched from significantly regulated genes using gprofiler. Enrichments were performed with DEGs from NOTCH3^{R182C} hiPSC-VSMCs (Patient 1). (F) Representative immunofluorescence images showing expression of NOTCH3 (green), SM22 (red) and nuclei (DNA) of hiPSC-VSMCs. 10x, scale bars 250 µm. (G) Quantification of hiPSC-VSMCs normalized intensity of NOTCH3, SM22 and area (um^2). Data are from N=3 three independent experiments and shown as ± SD. Unpaired t test. *$p < 0.05$, ns, not significant.

FIGURE S3

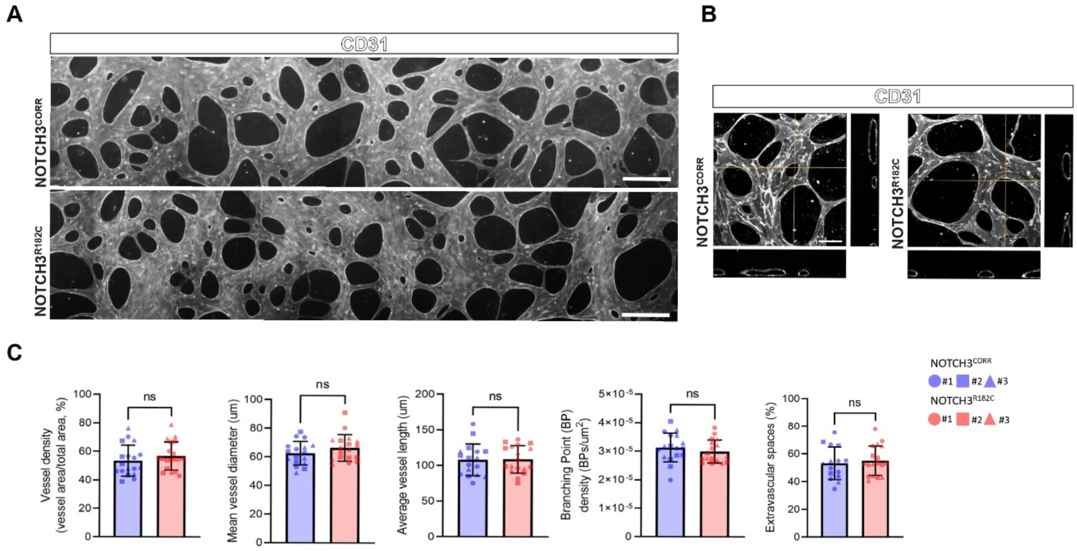

Figure S3. Vessel characterization of hiPSC-derived 3D Vessel-on-Chip.
(A) Representative images of vascular networks formed by hiPSC-ECs (CD31, grey). 10x, scale bars 200 µm. (B) Representative confocal images showing hiPSC-ECs (grey; CD31). Images displaying xyz, xy and yz cross-sectional perspectives. 40x, scale bars 100 µm. (C) Quantification of vessel density (%), mean diameter (µm), average vessel length (µm), branching point (BP) density (BPs/µm^2) and extravascular spaces (%).

FIGURE S4

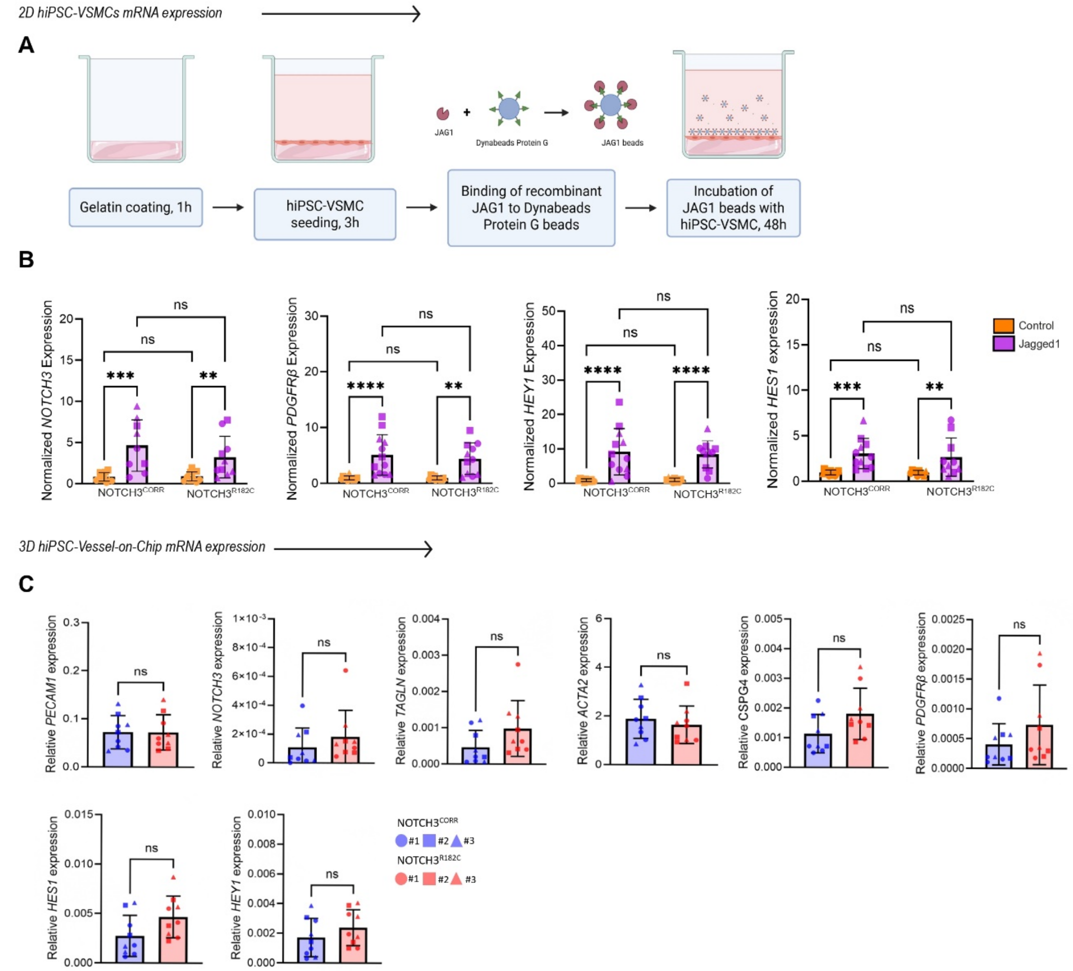

Figure S4. Expression profile of 2D hiPSC-VSMCs and hiPSC-derived 3D Vessel-on-Chip.

(A) Schematic representation of 2D Jagged1-bead assay in hiPSC-VSMCs. (B) Normalized mRNA expression of *NOTCH3, PDGFR, HES1 and HEY1 genes in 2D hiPSC-VSMCs in Control or Jagged1-activated conditions. (C)* Relative mRNA expression of *PECAM1, NOTCH3, TAGLN, CSPG4, PDGFRb, ACTA2, HES1* and *HEY* genes in 3D Vessel-on-Chip. Data are from N=3 three independent experiments and shown as ± SD. Unpaired t test. ****$p < 0.0001$, ***$p < 0.001$, **$p < 0.01$ *$p < 0.05$, ns, not significant.

METHODS

hiPSC lines

Research on hiPSC was approved by the medical ethical committee (P13.080) at Leiden University Medical Center, the Netherlands and written informed consent was obtained from all patients. PBMCs isolated from peripheral blood were used for reprogramming as described previously.(Bouma et al. 2020; Bouma et al. 2017) The following hiPSC lines were generated from Patient 1: LUMC0169iNOTCH and Patient 2: LUMC0194iNOTCH. hiPSCs were routinely cultured on Vitronectin according to the manufacturer's protocol. Pluripotency of the hiPSC clones was confirmed by expression of undifferentiated state markers OCT3/4, SSEA-4, NANOG using flow cytometry. The differentiation potential of the lines was confirmed by short-term differentiation assay in vitro, with subsequent immunofluorescence staining for markers of the three germ layers. G-banding analysis was conducted at the Laboratory of Clinical Genetics Leiden (LDGA). NIH Center for Regenerative Medicine hiPSC line (NCRM-1, generated from CD34+ cord blood cells, https://hpscreg.eu/cellline/CRMi003-A), obtained from RUDCR Infinite Biologics at Rutgers University, was modified in-house with a mCherry expression cassette under the human cytomegalovirus (hCMV) early enhancer/chicken β actin (CAG) promoter using a previously established protocol.(Rostovskaya et al. 2012)

CRISPR gene correction strategy

The heterozygous *NOTCH3* c.544C>T variant located in exon 4 was corrected by insertion of a single base (C) and simultaneous introduction of two silent variants using CRISPR/Cas9-induced homology direct repair (HDR). The targeting strategy is depicted in Figure 2A. The Streptococcus pyogenes Cas9 nuclease protein high fidelity (IDT) was used in combination with a single stranded oligo DNA (ssODN) with the corrected *NOTCH3* sequence. The silent mutations were introduced into the ssODN (5′-GTGGATGAGTGCCGGGTGGGTGAGCCCTGCCGCCATGGTGGCACCTGCCTCAACACA CCTGGCTCCTTCCGCTGTCAGTGTCCAGCTGGCTACACAGGGCCACTATGTGAGAACC CCGCGGTGCCCTGTGCGCCCTCACC -3′, IDT Ultramer) to block re-cutting of the Cas9 nuclease upon HDR and to avoid indel formation and to block restriction enzyme XcmI activity for screening. A CRISPR/Cas9 target site specific close to the variant (target site: CCA CTGTGTAGCCAGCTGGACAC) was identified using the design web tool crispor.tefor.net. CRISPR/Cas9 reagents were delivered as ribonucleoprotein (RNP) complex, composed of CRISPR/Cas9 guide RNA (crRNA:tracrRNA duplex) and the Cas9 nuclease protein (IDT). For genetic repair $1*10^5$ hiPSCs were electroporated using 2 electroporations (1300v 30ms 1 pulse) of the Neon Transfection System (Invitrogen) and subsequently cultured in 2 Synthemax II-SC (Corning)-coated wells of a 12-well plate in TESR-E8 with CloneR2 (Stem Cell Technologies). For single cell cloning, 1000 cells were plated onto a Synthemax II-SC-coated 10 cm dish in TESR-E8 with CloneR2. After 8–12 days hiPSC colonies were picked into VN-coated 2x wells of a 96-well plate in TESR-E8. The region of interest was amplified by PCR using the Terra PCR Direct Polymerase Mix (TaKaRa) from DNA isolated from one well (QuickExtract solution, Lucigen). Successfully edited clones were identified by enzyme XcmI activity and confirmed by Sanger sequencing performed by the Leiden Genome Technology Centre (LGTC).

Differentiation of hiPSCs towards ECs

hiPSCs were maintained in mTeSR-E8 and differentiated towards ECs using previously published protocols.(Orlova, van den Hil, et al. 2014; Orlova, Drabsch, et al. 2014) For mesoderm induction (day 0-3), mTeSR-E8 medium was replaced with B(P)EL medium supplemented with 8 μM CHIR99021 (Tocris Bioscience, 4423). Cells were refreshed with vascular specification medium comprised of VEGF (50 ng/ml) and 10 μM SB431542 (Tocris Bioscience, 1614) in B(P)EL at day 3, day 6, and day 9. hiPSC-ECs were isolated on day 10 using CD31-Dynabeads™ (Thermo Fisher Scientific), as previously described (Orlova et al., 2014b; 2014a). hiPSC-ECs were expanded in complete EC growth medium comprised of

Human Endothelial-serum free medium (EC-SFM) with 1% Human platelet poor serum (P2918, Sigma), VEGF (30 ng/ml) and bFGF (20 ng/ml), as described previously with minor modifications.(Orlova, van den Hil, et al. 2014; Orlova, Drabsch, et al. 2014) hiPSC-ECs were expanded for additional 3-4 days post-isolation and cryopreserved using serum-free cryopreservation medium at passage number 1 (P1) (CryoStor™CS10) (StemCell Technologies, 07930).

Differentiation of hiPSCs towards NCCs
hiPSC colonies were passaged and kept in hiPSC mTeSR-E8 and differentiated towards NCCs using previously published protocols.(Halaidych et al. 2019) After 2 days, the medium was changed to NC differentiation medium consisting of B(P)EL medium supplemented with 10 µM SB431542 (Tocris Bioscience, 1614), 1 µM CHIR99021 (Tocris Bioscience, 4423) and 10 ng/mL bFGF (Miltenyi Biotec, 130-093-842). Cells were refreshed every 2 days and kept in NC differentiation medium for 10-12 days. After 10-12 days NC cells (NCCs) were passaged with 1xTrypLE Select (Gibco, 12563029) and plated in 1:4 ratio on Matrigel-coated plates. hiPSC-NCCs were cryopreserved at P3 using serum-free cryopreservation medium (CryoStor™CS10) (StemCell Technologies, 07930).

Differentiation of hiPSC-NCCs towards VSMCs
hiPSC-NCCs were differentiated into VSMCs following a previously described protocol with minor modifications.(Halaidych et al. 2019) NCCs were plated at $3x10^4$ cells/cm^2 seeding density on 0.1% Gelatin (Sigma-Aldrich, G1890) coated plates in VSMC differentiation medium consisting of B(P)EL medium supplemented with 2 ng/mL TGF-β3 (PeproTech, 100-36E) and 10 ng/mL PDGF-BB (PeproTech, 100-14B). Cells were refreshed every 2 days and kept in VSMC differentiation medium for 8 days. Cells were passaged in a 1:4 splitting ratio at day 4. hiPSC-VSMCs were cryopreserved at P1 using serum-free cryopreservation medium (CryoStor™CS10) (StemCell Technologies, 07930).

Primary VSMCs culture
Primary brain VSMCs were isolated from post-mortem brain tissue. Briefly, connective tissue and arachnoid mater surrounding the arteries was removed. Arteries were washed in PBS and cut into 0.5 cm pieces and dissected longitudinally. The endothelial layer was removed mechanically by scraping across the bottom of a Petri dish and the arterial sections were fixated to the bottom of a sterile T25 flask containing 4 ml complete culture medium (DMEM-F12, glutaMAX, supplemented with 10% FCS, 2 µM MEM sodium pyruvate, 0.5 U/ml penicillin and 0.5 µg/ml streptomycin (all from Life Technologies, Bleiswijk, The Netherlands). Flasks were first incubated in an upright position at 5% CO_2, 37°C, to ensure tissue binding. After 4 hours the flasks were placed in a horizontal position to cover the arterial sections with culture medium. First cellular outgrowth was seen 2 weeks after isolation. Cells were cryopreserved at P3-4 using culture medium supplemented with 20% fetal calf serum and 10% DMSO.

RNA sequencing analysis
Total RNA was extracted using the NucleoSpin RNA XS kit (Macherey-Nagel, cat no. 740902.50) according to the manufacturer's instructions. Whole transcriptome data were generated at Novogene (Cambridge, UK) using the Illumina Sequencing PE150 (PE150, Q30≥85 %). RNAseq reads were processed using the opensource BIOWDL RNAseq pipeline v5.0.0 (biowdl/RNA-seq: Release 5.0.0 (zenodo.org) developed at the LUMC.[7] This pipeline performs FASTQ pre-processing (including quality control, quality trimming, and adapter clipping), alignment, read quantification, and optionally transcript assembly. FastQC (v0.11.9) was used for checking raw read QC. Adapter clipping was performed using Cutadapt (v2.10) with the default settings. RNAseq reads' alignment was performed using STAR (v2.7.5a) on human reference genome GRCh38. The gene read quantification was performed using HTSeq-count (v0.12.4) with the Ensembl gene annotation version 110.(Anders, Pyl, and Huber 2015) The resulting count matrix was transformed into log counts per million (cpm) and

the sources of variation was inspected with principal component analysis. It was observed that 23.1 % of the total variation within the data is due to the difference in the two patients. It is thus clear that the difference in the genetic make up between the two patients is responsible for a large source of variation thereby introducing confounding factors. For further processing of the data, we opted to continue with the cells obtained from patient 1 for downstream analysis. The RNAseq data was further processed in R (version 4.3.3) using the Bioconductor package edgeR.[8] Briefly, the genes with low counts were filtered by their expression, requiring expression in at least one of the conditions followed by normalization of the library using trimmed mean of the M-values (TMM) and the dispersion was estimated using the quantile-adjusted conditional maximum likelihood (qCML) method. Finally, differential gene expression was determined using likelihood ratio tests and multiple hypothesis testing was corrected for using Benjamini-Hochberg False discovery rate. The differentially expressed genes were defined based on a log fold change > 1 or < -1, a q-value < 0.05 and a logCPM > 0. Plots depicting differential gene expression was also created in the same R version using ggplot2. Gene ontology enrichment analysis was used to assess biological functions based on gene expression by using the differentially expressed genes as defined above. Enrichments were calculated using gprofiler using both the up and down regulated genes using the default parameters.(Kolberg et al. 2023) The full list of differentially expressed genes and gprofiler enrichment is available in table S1.

FACS NCCs

Cells were dissociated with 1xTrypLE Select and washed once with FACs buffer containing 10% FBS, and once with FACs buffer. The following surface antibody NGFRBV421 (BD Biosciences, 562562, 1:100) was used. For intracellular labelling with SOX2-A488 (eBiosciences, 53-9811-80, 1:50) the cells were fixed and permeabilized using BD Cytofix/Cytoperm kit (BD Biosciences, 554714). Analysis of samples was performed on the MACSQuant VYB (Miltenyi Biotec, 130-096-116) equipped with the following lasers/filters: Violet/405 nm BV421: 450/50, Blue/488 nm FITC, A488: 525/50, Yellow/561 nm PE: 586/15, Yellow/561 nm PEVio-770: 750 nm LP.

Immunofluorescence 2D staining and microscopy

VSMCs were grown in Fibronectin-coated 96 well plates (Corning, 353219) to a confluent monolayer. Cells were fixed with 4% PFA, permeabilized with 0.05% TX-100 (Sigma). the following antibodies were used: Primary antibodies NOTCH3 (Sigma, 1E4, 1:200); SM22 (Abcam, ab14106, 1:200). VSMCs were imaged using EVOS M7000 using 10x magnification objective. Images were quantified using pipelines developed on the free open source CellProfiler software (https://cellprofiler.org/).(Carpenter et al. 2006)

Assessment of 2D contractile hiPSC-VSMCs properties

hiPSC-VSMCs were passaged as single cells and plated in a Gelatine-coated 96 well plate at density ~$2*10^4$ cells/cm2 in B(P)EL medium and kept in a CO2 incubator overnight before functional analysis as previously described.(Halaidych et al. 2019) Cells were loaded with 2 µM Calcein AM (Ex/Em=494/517 nm, Invitrogen L3224) for 30 min in a live imaging chamber (37°C, 5% CO2, humidified). After the staining cells were gently washed with B(P)EL medium before assessment of the contraction. Series of images of Calcein fluorescence were captured using a Leica AF6000 microscope with a 10x objective and 4x4 automated stitching. First, the basal state of cells was acquired. Then a negative control was obtained by adding B(P)EL medium and fluorescence was acquired after 30 min. Finally, cells were stimulated with ET-I at a final concentration of 0.1 µM and fluorescence was acquired after 30 minutes. Images were processed using a customized pipeline that included automated cell identification and tracking using CellProfiler as previously described.(Halaidych et al. 2019) Output data were analyzed using a customized R-based script.

Assessment of 2D hiPSC-VSMCs Intracellular Ca2+ release

hiPSC-VSMCs were passaged as single cells and plated in a bottomed Fibronectin-coated 96-well plate at density ~5*10^4 cells/cm2 in B(P)EL medium and kept in a CO2 incubator overnight before functional analysis Intracellular Ca2+ release was assessed in hiPSC-VSMCs at day one post-seeding in a black, flat-. The calcium-6 dye (Molecular Devices) was dissolved in 10 mL HBSS buffer B and subsequently diluted 1:4 in Buffer B (Molecular Devices). The diluted dye solution was added 1:1 to the wells containing B(P)EL medium. hiPSC-VSMCs were incubated for 2 hours at 37 °C with 5% CO2 before being measured on the FDSS/µcell (Hamamatsu Photonics) at 37 °C with an exposure time of 0.1s. Response to ET-I stimulus was performed by first preparing a "compound plate" including a medium control of B(P)EL medium and 1 µM ET-I (Sigma, A9187) in B(P)EL medium. Control and ET-I stimulus were automatically mixed and injected (20ul)into the assay plate (180 ul per well), reaching final ET-I concentrations of 0.1 µM. Analysis was performed in R (4.0.3) and the induced change in Ca2+ release was calculated by quantification of the area under the curve of the average fluorescence intensity normalized to time 0 (s).

Activation of NOTCH3 signalling in 2D hiPSC-VSMCs

Activation of the NOTCH3 signalling pathway in 2D using Jagged1 beads was performed according as previously described with minor modifications.(Zohorsky, Lin, and Mequanint 2021) hiPSC-VSMC were seeded on gelatin-coated plates in EGM-2 medium at a density of 20.000 cells/cm^2 and were cultured for 3 hours to ensure cell attachment. For the preparation of the Jagged1 beads, 600 ug of Dynabeads Protein G (30 mg/ml, Invitrogen) were washed with PBS-T 0.02% and then incubated with 2.5 ug of recombinant Jagged1-FC chimera (diluted in PBS-T 0.02%, R&D systems) for 10 minutes under rotation in room temperature. Afterwards, Jagged1 beads were washed with PBS and resuspended in EGM-2 medium. Finally, the cells were incubated with Jagged1 beads for 48 hours at a concentration of 18 nM; plain Dynabeads Protein G beads were used as a negative control. After 48 h, the cells were harvested for RNA isolation and downstream qPCR analysis.

Cell preparation prior 3D Vessel-on-Chip culture

hiPSC-ECs (P1) were thawed and cultured on gelatin-coated plates in complete EC growth medium composed of Human Endothelial-SFM (EC-SFM) with 1% platelet poor serum (PPS), VEGF (30 ng/ml) and bFGF (20 ng/ml),4 days prior to 3D Vessel-on-Chip seeding. hiPSC-VSMCs (P1) were thawed and cultured on gelatin-coated plates in B(P)EL medium supplemented with 2 ng/mL TGF-β3 (PeproTech, 100-36E) and 10 ng/mL PDGF-bb (PeproTech, 100-14B) 4 days prior to 3D Vessel-on-Chip seeding using previously described protocol with minor modifications.(Halaidych et al. 2019) Primary VSMCs (P4-6) were thawed and cultured on gelatin-coated plates in complete culture medium composed of DMEM-F12, glutaMAX, supplemented with 10% FCS, 2 µM MEM sodium pyruvate, 0.5 U/ml penicillin and 0.5 µg/ml streptomycin (all from Life Technologies, Bleiswijk, The Netherlands).

Immunofluorescence 3D staining, Microscopy and analysis

After 7 days of culture in, cells in 3D Vessel-on-Chip were fixed *in situ* in 4% paraformaldehyde (PFA) for 30 min at RT. Cell plasma membranes were permeabilized with 0.5% Triton X-100 for 15 min at RT and washed 3 times for 10 mins between each step with PBS, then blocking buffer (2% BSA) was added for 3 hours at RT. Primary antibodies (1:200 volume ratio in 1% BSA), against CD31 (PECAM1, Mouse, M0823, DAKO or Sheep, AF806, R&D systems), VE-Cadherin (Rabbit, 2158S, Cell Signaling), NOTCH3 (Mouse, 1E4, Sigma-Aldrich) SM22 (TAGLIN; Rabbit, ab14106, Abcam), PDGFRb (Goat, AF385, R&D systems), aSMA (ACTA2; Mouse, 1A4, Sigma-Aldrich) and Integrin β1 (CD29; TS2/16, Thermo-Fisher) were incubated overnight at 4 ºC. Secondary antibodies (1:300 volume ratio in 1% BSA) and F-Actin (Phalloidin labelling probe GFP; 1:100, Thermo-Fisher), were incubated for 2 hours at RT after 3 times 15 min PBS washes. Vessel-on-Chip were imaged using EVOS M7000 using 10x magnification objective. A customised plate layout that allowed for automated imaging and stitching to produce images of complete microfluidic channel for all fluorescent channels was used. Images from the whole microfluidic channel (acquired using EVOS) were quantified

using pipelines developed on the free open source CellProfiler software (https://cellprofiler.org/) as previously described.(Vila Cuenca et al. 2021; Orlova et al. 2022) Two filter steps were applied to images of vascular network to reduce non-specific segmentation from cell junctions and a minimum cross-entropy thresholding method was used to produce a binarized image. The binarized images from the CellProfiler output were then analyzed using the freely available ImageJ software with the plugin (https://imagej.nih.gov/ij/ ,https://imagej.net/DiameterJ).(Hotaling et al. 2015) For 3D stacks, images were taken using a DragonFly spinning disk (Andor) microscope with 40x and 63x magnification objective and post-processing performed and processed using Imaris 9.5 software (Bitplane, Oxford Instruments). For 3D quantitative analysis, surface-rendering was performed and processed using Imaris 9.5 software (Bitplane, Oxford Instruments) as previously described.(Vila Cuenca et al. 2021; Orlova et al. 2022) VSMCs morphological analysis were performed with the visually aided morphophenotyping image recognition VAMPIRE software.(Phillip et al. 2021)

RNA isolation and quantitative RT-PCR

Total RNA was isolated from the microfluidic devices at end-point day 7 as previously described. Cells were extracted by dissolving the extracellular matrix / fibrin mix with Collagenase B (1 mg/ml, Roche, 11088815001) for half an hour at 37 degrees °C, while rocking. RNA was extracted using the NucleoSpin RNA XS kit (Macherey-Nagel) and cDNA was synthesized using an iScript-cDNA Synthesis kit (Bio-Rad). iTaq Universal SYBR Green Supermixes (Bio-Rad) and Bio-Rad CFX384 real-time system were used for the PCR reaction and detection. Relative gene expression was calculated using the delta Ct calculation and normalized to the housekeeping gene hARP.

LV production

LV particles were produced essentially as described previously(Liu et al. 2018) except that PEI MAX 40K (Polysciences Europe, Hirschberg an der Bergstraße, Germany) instead of PEI 25K was used as transfection agent and the polyethyleneimine-DNA complexes were left on the cells for only 4 hours.

Plasmid constructs

The lentiviral vector (LV) shuttle plasmid pLV.hCMV-IE.GCaMP6f.IRES.PurR.hHBVPRE was generated in a multistep procedure using pGP-CMV-GCaMP6f (Addgene, Watertown, MA; plasmid number 40755) and pLV.hCMV-IE.IRES.PurR.hHBVPRE as starting constructs.(Neshati et al. 2014) pLV.hCMV-IE.GCaMP6f.IRES.PurR.hHBVPRE contains a human cytomegalovirus immediate-early gene (hCMV-IE) promoter driving expression of a bicistronic mRNA encoding the ultra-sensitive $[Ca^{2+}]_{cyt}$ sensor GCaMP6f ref and *Streptomyces alboniger* puromycin-N-acetyltransferase. The LV shuttle plasmid pLV.hCMV-IE.eGFP.PurR.hHBVPRE was generated by insertion of the *Aequorea victoria* enhanced green fluorescent protein (eGFP)-encoding 754-bp SmaI×EcoRI fragment of pEGFP (Clontech - Takara Bio Europe, Saint-Germain-en-Laye, France) behind the hCMV-IE promoter of pLV.hCMV-IE.IRES.PurR.hHBVPRE. To this end, the insert was combined with the 8122-bp SmaI×EcoRI fragment of pLV.hCMV-IE.IRES.PurR.hHBVPRE.(Neshati et al. 2014) Recombinant plasmid construction was done with enzymes from New England Biolabs (Bioké, Leiden, the Netherlands) or Fermentas (ThermoFisher Scientific) using standard procedures or following the instructions provided with specific reagents. The plasmids were amplified in *Escherichia coli* GeneHogs (ThermoFisher Scientific) cells and purified using LabNed Plasmid Maxiprep Kits (ITK diagnostics, Uithoorn, the Netherlands).

LV transduction of hiPSC-VSMCs

The LV shuttle plasmid pLV.hCMV.-IE.GCaMP6f(+).IRES.PurR.hHBVPRE was used to express GCaMP6f in LUMC0054iCTRL hiPSC-NCCs P3 at previously described.(Vila Cuenca et al. 2021) The LV shuttle plasmid pLV.hCMV.-IE.eGFP.IRES.PurR.hHBVPRE) was used to express pEGFP in LUMC0054iCTRL hiPSC-NCCs P3. Briefly, one day after seeding 40.000 cells/12-well on Matrigel-coated plates, hiPSC-NCCs were transduced with 2.5 µl viral

particles in B(P)EL medium overnight. 96h post-transduction with complete NC differentiation medium infected cells were selected with 1 µg/mL puromycin (Sigma, P7255). After 4 days, remaining cells were expanded (1:3 ratio) Matrigel-coated plates then dissociated with 1xTrypLE Select and cryopreserved at passage number 3 (P3) using serum-free cryopreservation medium (CryoStor™CS10) (StemCell Technologies, 07930). Next, hiPSC-NC cells (hiPSC-NCCs) were differentiated into hiPSC-VSMCs as described previously with minor modifications.(Vila Cuenca et al. 2021)

Assessment of intracellular Ca^{2+} release in the 3D Vessel-on-Chip

Intracellular Ca^{2+} release upon medium refreshment and upon stimulation with the vasoconstrictor (ET-I) was analyzed on day 7 of Vessel-on-Chip culture as preformed previously.(Vila Cuenca et al. 2021) The generation and transduction in hiPSC-VSMCs of the lentiviral vector shuttle plasmid pLV.hCMV-IE.GCaMP6f.IRES.PurR.hHBVPRE is described in Supplemental Methods. Sequences of images prior to- (basal state) and after medium refreshment were captured using EVOS M7000 with a 10x objective. For the medium refreshment, medium from all ports of the microfluidic channel was first removed and gravity-driven flow was induced by the addition of 100 µl medium to the right media ports and 50 µl medium to left connecting media ports. After 30s, fluorescence activity of the whole microfluidic channel was captured. For real-time intracellular Ca^{2+} release upon stimulation with the vasoconstrictor, the microfluidic chip was placed into a humidified live cell imaging chamber (+37°C, 5% CO_2) and mounted on a DragonFly spinning disk microscope (Andor) with a 20x magnification objective on day 7 of culture. First, medium from all ports was removed and refreshed with 30 µl of EGM-2. After 30 min, basal fluorescence activity was captured for 5 seconds. Next, gravity-driven flow was induced by the addition of 60 µl EGM-2 or EGM-2 supplemented with 1.5 µM ET-I (Sigma) with a final concentration of 1 µM after the addition to the right medium ports containing 30 µl of EGM-2. Then, simultaneous image capturing was continued for 160 seconds. Image sequences of fluorescence were captured at 4 frames per second. After simultaneous image capturing, confocal images were acquired to create a 3D stack and processed using Imaris 9.5 software (Bitplane, Oxford Instruments). Images sequences were processed using a freely available plugin ‘‘LC Pro’’ for ImageJ (https://imagej.nih.gov/ij/plugins/lc-pro/index.html).(Yip and Sham 2012) Free open-source CellProfiler software (https://cellprofiler.org/) was used to determine the total number of cells in a field of view. Output data were analysed as previously described.(Vila Cuenca et al. 2021; Halaidych et al. 2019)

REFERENCES

Anders, S., P. T. Pyl, and W. Huber. 2015. 'HTSeq--a Python framework to work with high-throughput sequencing data', *Bioinformatics*, 31: 166–9.

Bouma, M. J., V. Orlova, F. E. van den Hil, H. J. Mager, F. Baas, P. de Knijff, C. L. Mummery, H. Mikkers, and C. Freund. 2020. 'Generation and genetic repair of 2 iPSC clones from a patient bearing a heterozygous c.1120del18 mutation in the ACVRL1 gene leading to Hereditary Hemorrhagic Telangiectasia (HHT) type 2', *Stem Cell Res*, 46: 101786.

Bouma, M. J., M. van Iterson, B. Janssen, C. L. Mummery, D. C. F. Salvatori, and C. Freund. 2017. 'Differentiation-Defective Human Induced Pluripotent Stem Cells Reveal Strengths and Limitations of the Teratoma Assay and In Vitro Pluripotency Assays', *Stem Cell Reports*, 8: 1340–53.

Carpenter, A. E., T. R. Jones, M. R. Lamprecht, C. Clarke, I. H. Kang, O. Friman, D. A. Guertin, J. H. Chang, R. A. Lindquist, J. Moffat, P. Golland, and D. M. Sabatini. 2006. 'CellProfiler: image analysis software for identifying and quantifying cell phenotypes', *Genome Biol*, 7: R100.

Halaidych, O. V., A. Cochrane, F. E. van den Hil, C. L. Mummery, and V. V. Orlova. 2019. 'Quantitative Analysis of Intracellular Ca(2+) Release and Contraction in hiPSC-Derived Vascular Smooth Muscle Cells', *Stem Cell Reports*, 12: 647–56.

Hotaling, N. A., K. Bharti, H. Kriel, and C. G. Simon, Jr. 2015. 'DiameterJ: A validated open source nanofiber diameter measurement tool', *Biomaterials*, 61: 327–38.

Kolberg, L., U. Raudvere, I. Kuzmin, P. Adler, J. Vilo, and H. Peterson. 2023. 'g:Profiler-interoperable web service for functional enrichment analysis and gene identifier mapping (2023 update)', *Nucleic Acids Res*, 51: W207–W12.

Liu, J., L. Volkers, W. Jangsangthong, C. I. Bart, M. C. Engels, G. Zhou, M. J. Schalij, D. L. Ypey, D. A. Pijnappels, and A. A. F. de Vries. 2018. 'Generation and primary characterization of iAM-1, a versatile new line of conditionally immortalized atrial myocytes with preserved cardiomyogenic differentiation capacity', *Cardiovasc Res*, 114: 1848–59.

Neshati, Z., J. Liu, G. Zhou, M. J. Schalij, and A. A. de Vries. 2014. 'Development of a lentivirus vector-based assay for non-destructive monitoring of cell fusion activity', *PLoS One*, 9: e102433.

Orlova, V. V., Y. Drabsch, C. Freund, S. Petrus-Reurer, F. E. van den Hil, S. Muenthaisong, P. T. Dijke, and C. L. Mummery. 2014. 'Functionality of endothelial cells and pericytes from human pluripotent stem cells demonstrated in cultured vascular plexus and zebrafish xenografts', *Arterioscler Thromb Vasc Biol*, 34: 177–86.

Orlova, V. V., D. M. Nahon, A. Cochrane, X. Cao, C. Freund, F. van den Hil, C. J. J. Westermann, R. J. Snijder, J. K. Ploos van Amstel, P. Ten Dijke, F. Lebrin, H. J. Mager, and C. L. Mummery. 2022. 'Vascular defects associated with hereditary hemorrhagic telangiectasia revealed in patient-derived isogenic iPSCs in 3D vessels on chip', *Stem Cell Reports*, 17: 1536–45.

Orlova, V. V., F. E. van den Hil, S. Petrus-Reurer, Y. Drabsch, P. Ten Dijke, and C. L. Mummery. 2014. 'Generation, expansion and functional analysis of endothelial cells and pericytes derived from human pluripotent stem cells', *Nat Protoc*, 9: 1514–31.

Phillip, J. M., K. S. Han, W. C. Chen, D. Wirtz, and P. H. Wu. 2021. 'A robust unsupervised machine-learning method to quantify the morphological heterogeneity of cells and nuclei', *Nat Protoc*, 16: 754–74.

Rostovskaya, M., J. Fu, M. Obst, I. Baer, S. Weidlich, H. Wang, A. J. Smith, K. Anastassiadis, and A. F. Stewart. 2012. 'Transposon-mediated BAC transgenesis in human ES cells', *Nucleic Acids Res*, 40: e150.

Vila Cuenca, M., A. Cochrane, F. E. van den Hil, A. A. F. de Vries, S. A. J. Lesnik Oberstein, C. L. Mummery, and V. V. Orlova. 2021. 'Engineered 3D vessel-on-chip using hiPSC-derived endothelial- and vascular smooth muscle cells', *Stem Cell Reports*, 16: 2159–68.

Yip, K. P., and J. S. Sham. 2012. 'Tracking stars: automated two-dimensional analysis of Ca(2)(+) events. Focus on "Automated region of interest analysis of dynamic Ca(2)(+) signals in image sequences"', *Am J Physiol Cell Physiol*, 303: C233–5.

Zohorsky, K., S. Lin, and K. Mequanint. 2021. 'Immobilization of Jagged1 Enhances Vascular Smooth Muscle Cells Maturation by Activating the Notch Pathway', *Cells*, 10.
