# Supplementary material for: 3D Vessels-on-Chip using isogenic hiPSC-derived VSMCs reveal NOTCH3-driven alterations in brain small vessel disease
Source: Stem Cell Reports. 2026 Mar 26;21(4):102863. doi: 10.1016/j.stemcr.2026.102863 (PMC13083807; doi:10.1016/j.stemcr.2026.102863)
Supplement: Document S2. Article plus supplemental information [file mmc6.pdf]

STEM CELL REPORTS
Article
OPEN ACCESS

3D Vessels-on-Chip using isogenic hiPSC-derived VSMCs reveal NOTCH3-driven alterations in brain small vessel disease

Marc Vila Cuenca,[1,2] Theano Tsikari,[2] Minne N. Cerfontaine,[1] James L. Gallant,[2] Francijna E. van den Hil,[2] Marga J. Bouma,[3] Kyra L. Dijkstra,[4] Gido Gravesteijn,[1] Antoine A.F. de Vries,[5] Christine L. Mummery,[2] Julie W. Rutten,[1,6,*] Saskia A.J. Lesnik Oberstein,[1,6,*] and Valeria V. Orlova[2,6,7,*]
[1]Department of Clinical Genetics, Leiden University Medical Center, Leiden, the Netherlands
[2]Department of Anatomy & Embryology, Leiden University Medical Center, Leiden, the Netherlands
[3]Leiden University Medical Center hiPSC Center, Leiden, the Netherlands
[4]Department of Pathology, Leiden University Medical Center, Leiden, the Netherlands
[5]Department of Cardiology, Leiden University Medical Center, Leiden, the Netherlands
[6]Senior author
[7]Lead contact
*Correspondence: j.w.rutten@lumc.nl (J.W.R.), s.a.m.j.lesnik@lumc.nl (S.A.J.L.O.), v.orlova@lumc.nl (V.V.O.)
https://doi.org/10.1016/j.stemcr.2026.102863

SUMMARY

cerebral autosomal dominant arteriopathy with subcortical infarcts and leukoencephalopathy (CADASIL) is a hereditary brain small vessel disease caused by pathogenic variants in the *NOTCH3* gene, leading to NOTCH3 protein accumulation and degeneration of vascular smooth muscle cells (VSMCs). Here, we developed a CADASIL 3D Vessel-on-Chip model using either primary brain VSMCs or human induced pluripotent stem cell (hiPSC)-derived VSMCs from CADASIL patients and isogenic controls. In 3D co-culture with hiPSC-derived endothelial cells, both primary and hiPSC-derived CADASIL VSMCs exhibited disease-relevant morphological abnormalities, increased NOTCH3 and contractile protein levels, and altered intracellular Ca^{2+} dynamics that were not observed under conventional 2D culture. PDGFRβ, a downstream NOTCH3 target, was upregulated and correlated with NOTCH3 protein levels in both 3D models and CADASIL patient brain tissue. Pharmacological inhibition of NOTCH3 cleavage reduced NOTCH3 protein levels and rescued CADASIL VSMC phenotypic abnormalities. In conclusion, this 3D Vessel-on-Chip model robustly shows CADASIL pathology-relevant readouts and provides a platform for mechanistic studies and therapeutic testing.

INTRODUCTION

The most prevalent genetic brain small vessel disease (SVD) is cerebral autosomal dominant arteriopathy with subcortical infarcts and leukoencephalopathy (CADASIL), a condition driven by pathogenic *NOTCH3* variants causing toxic gain of function through NOTCH3 protein aggregation in the blood vessel wall (Joutel et al. 1996, 2000). These *NOTCH3* variants occur in approximately 1 in 300 individuals worldwide and are associated with a broad spectrum of SVD severity (Rutten et al. 2014, 2016, 2020; Hack et al., 2022). CADASIL represents the severe end of the NOTCH3-associated SVD spectrum, with middle-age onset of stroke and vascular dementia (Wardlaw et al. 2019; Dupre, Drieu, and Joutel 2024). Disease-modifying therapies are currently only in pre-clinical development (Oliveira et al., 2023; Ghezali et al., 2018; Machuca-Parra et al., 2017).

The cell types primarily affected in CADASIL are vascular smooth muscle cells (VSMCs) and pericytes. NOTCH3 is a transmembrane receptor, which is predominantly expressed by VSMCs (Chabriat et al., 2009), and is essential for maintaining their differentiated state (Romay et al., 2024; Domenga et al., 2004). Endothelial cells (ECs) modulate NOTCH3 signaling through interactions with its ligands, such as Jagged and Delta-like proteins (Domenga et al., 2004; Villa et al., 2001). In CADASIL, NOTCH3 signaling seems to be largely preserved (Dupre, Drieu, and Joutel 2024; Dupre et al., 2024; Hack et al., 2023), while the ectodomain of the mutant NOTCH3 protein ($NOTCH3^{ECD}$) accumulates in the extracellular space (Joutel et al., 2000), with sequestration of extracellular matrix proteins (Zellner et al., 2018; Capone et al., 2016; Dupre, Drieu, and Joutel 2024), leading to vessel wall pathology characterized by degeneration of VSMCs (Joutel et al., 2000; Dupre, Drieu, and Joutel 2024; Chabriat et al., 2009).

Multiple *in vitro* models for CADASIL have been developed to investigate the disease mechanisms, but each has faced challenges in recapitulating VSMC and vascular pathology. Moreover, many models use primary cells derived from patients, which have limited availability (Dupre, Drieu, and Joutel 2024; Tikka et al., 2012; Neves et al., 2019; Ihalainen et al., 2007). Efforts to model CADASIL using patient-derived human induced pluripotent stem cells (hiPSCs) have shown promise (Ahn et al., 2024; Kelleher et al., 2019; Ling et al., 2019), but thus far, diseased hiPSC derivatives have only been compared with non-isogenic healthy controls: different genetic background may mask or erroneously

Published by Elsevier Inc. on behalf of International Society for Stem Cell Research.
This is an open access article under the CC BY license (http://creativecommons.org/licenses/by/4.0/).

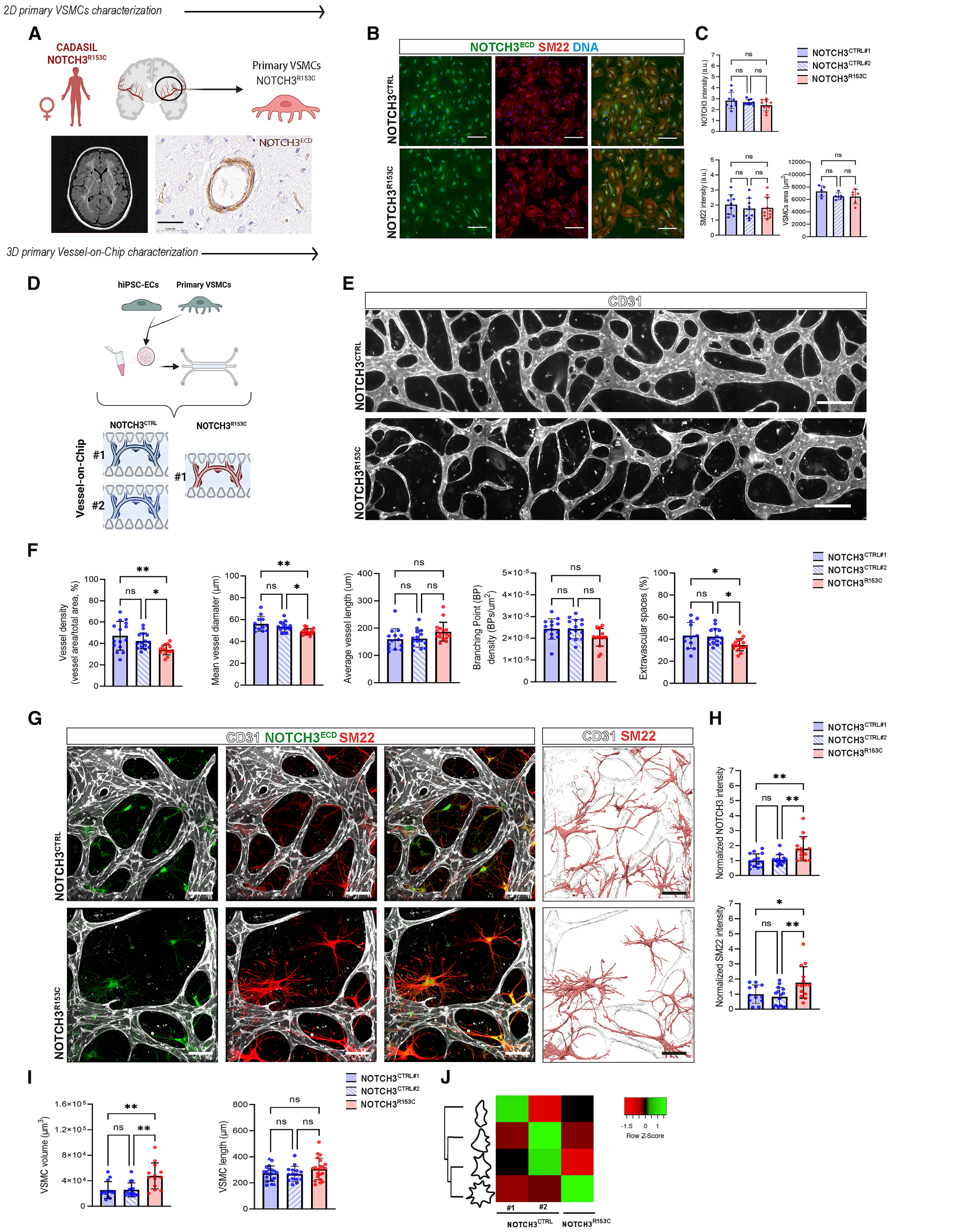

Figure 1. Primary CADASIL VSMCs reveal increased NOTCH3 levels and VSMC phenotypical changes in 3D Vessel-on-Chip

(A) Schematic representation showing NOTCH3^{R153C} primary VSMC isolation from brain blood vessels. Brain magnetic resonance imaging (MRI) of the patient showed the typical CADASIL neuroimaging hallmarks, including confluent white matter hyperintensities and lacunes. NOTCH3 immunohistochemistry of skin vessels showed pathognomonic NOTCH3 protein accumulation. Scale bar, 50 μm.

(legend continued on next page)

suggest the presence of disease-related features, which actually only reflect line-to-line differences. In addition, these models are frequently 2D cell cultures, which fail to emulate the integrated, complex, and multicell-type composition of the human vasculature. Vessel-on-Chip technology is emerging as an innovative approach offering standardized 3D engineered microscalable systems designed to replicate the vascular cellular microenvironment. Previously, we developed a 3D Vessel-on-Chip model that integrates ECs and VSMCs derived from hiPSC, effectively recapitulating the multicellular architecture of human vasculature (Vila Cuenca et al., 2021). Using isogenic, patient-specific hiPSC-derived vascular cells, we demonstrated that this platform can effectively capture hereditary vascular diseases (Orlova et al., 2022).

Here, we investigated whether this system would support modeling of other brain SVDs, such as CADASIL. We developed a CADASIL 3D Vessel-on-Chip model using either primary brain VSMCs or hiPSC-derived VSMCs from CADASIL patients, along with their isogenic controls. We found that CADASIL VSMCs exhibit increased NOTCH3 protein levels when in contact with ECs in the 3D Vessel-on-Chip environment, which was associated with changes in VSMC morphology, altered expression of contractile proteins, and actin cytoskeleton and focal adhesion disorganization. PDGFRβ levels increased in parallel with NOTCH3, which reflected findings in CADASIL patient brain tissue. Additionally, CADASIL VSMCs showed altered Ca^{2+} dynamics in the 3D Vessel-on-Chip. Finally, inhibition of NOTCH3 cleavage reduced NOTCH3 protein levels and restored VSMC morphological and functional differences. Thus, we show that pathogenic *NOTCH3* variants lead to phenotypic changes in VSMCs upon interaction with ECs, which can be reversed by targeting NOTCH3 cleavage. Our hiPSC-derived CADASIL 3D Vessel-on-Chip model provides a robust platform for *in vitro* pathomechanistic studies and testing potential disease-modifying therapeutic compounds.

RESULTS

Primary brain VSMCs from CADASIL patients show phenotypic changes and increased NOTCH3^{ECD} protein in a 3D Vessel-on-Chip model

To develop an informative *in vitro* model for CADASIL, we first examined how primary VSMCs cultured from vessels isolated from postmortem brain tissue of a CADASIL patient with the *NOTCH3* c.457C>T; p.(Arg153Cys) variant (NOTCH3^{R153C}) behaved in 2D versus 3D Vessel-on-Chip systems compared to primary VSMCs from two control donors (NOTCH3^{CTRL1} and NOTCH3^{CTRL2}, Figure 1A). In 2D immunofluorescence analysis, NOTCH3^{R153C} VSMCs showed no significant differences in intensity of NOTCH3^{ECD} and the contractile marker SM22 (TAGLN gene product), or in surface area compared to primary control VSMCs (NOTCH3^{CTRL}) (Figures 1B and 1C). Next, we integrated the primary brain VSMCs together with control hiPSC-ECs in a fibrin hydrogel to engineer 3D Vessel-on-Chip models as previously described (Figure 1D) (Vila Cuenca et al., 2021; Orlova et al., 2022). After 7 days of co-culture, 3D Vessel-on-Chip containing NOTCH3^{R153C} VSMCs showed changes in vessel morphology including significantly reduced vessel density, vessel diameter, and extravascular spaces compared to NOTCH3^{CTRL}, while no differences were observed in branching point density or average vessel length (Figures 1E and 1F). In contrast to the 2D cultures, NOTCH3^{R153C} VSMCs in the 3D Vessel-on-Chip showed higher levels of NOTCH3^{ECD} and SM22 (Figures 1G and 1H). These changes were accompanied by morphological changes in NOTCH3^{R153C} VSMCs, including increased volume and stellate morphology with prevalence of protrusions, while NOTCH3^{CTRL} VSMCs largely retained a spindle-like morphology

(B) Representative immunofluorescence images showing expression of NOTCH3 (green), SM22 (red), and nuclei (blue) of primary VMSCs. Magnification: 10×, scale bars, 250 μm.
(C) Quantification of NOTCH3 and SM22 intensity (a.u.) and area (μm^2) of primary VSMCs.
(D) Schematic representation of 3D Vessel-on-Chip experiments with primary VMSCs. Primary VSMCs from two control patients (NOTCH3^{CTRL}) and one CADASIL patient (NOTCH3^{R153C}) were cultured with hiPSC-ECs in microfluidic devices.
(E) Representative images of whole vascular networks formed by hiPSC-ECs (CD31, gray). Magnification: 10×, scale bars, 200 μm.
(F) Quantification of vessel density (%), mean vessel diameter (μm), average vessel length (μm), branching point (BP) density (BPs/μm^2), and extravascular spaces (%).
(G) Representative confocal images of microvascular network showing hiPSC-ECs (gray; CD31) and primary VSMCs (green; NOTCH3, red; SM22) and surface-rendered images. Magnification: 40×, scale bars, 100 μm.
(H) Quantification of the normalized intensities of NOTCH3 and SM22 in primary VSMCs.
(I and J) Quantification of primary VSMC volume (μm^3, I) and length (μm, I) and heatmap comparing the abundance of primary VSMCs falling into four different cell shapes (J).
Data are from $N = 3$ three independent experiments and shown as ± SD. One-way ANOVA test. $^{**}p < 0.005$, $^{*}p < 0.05$, ns, not significant.

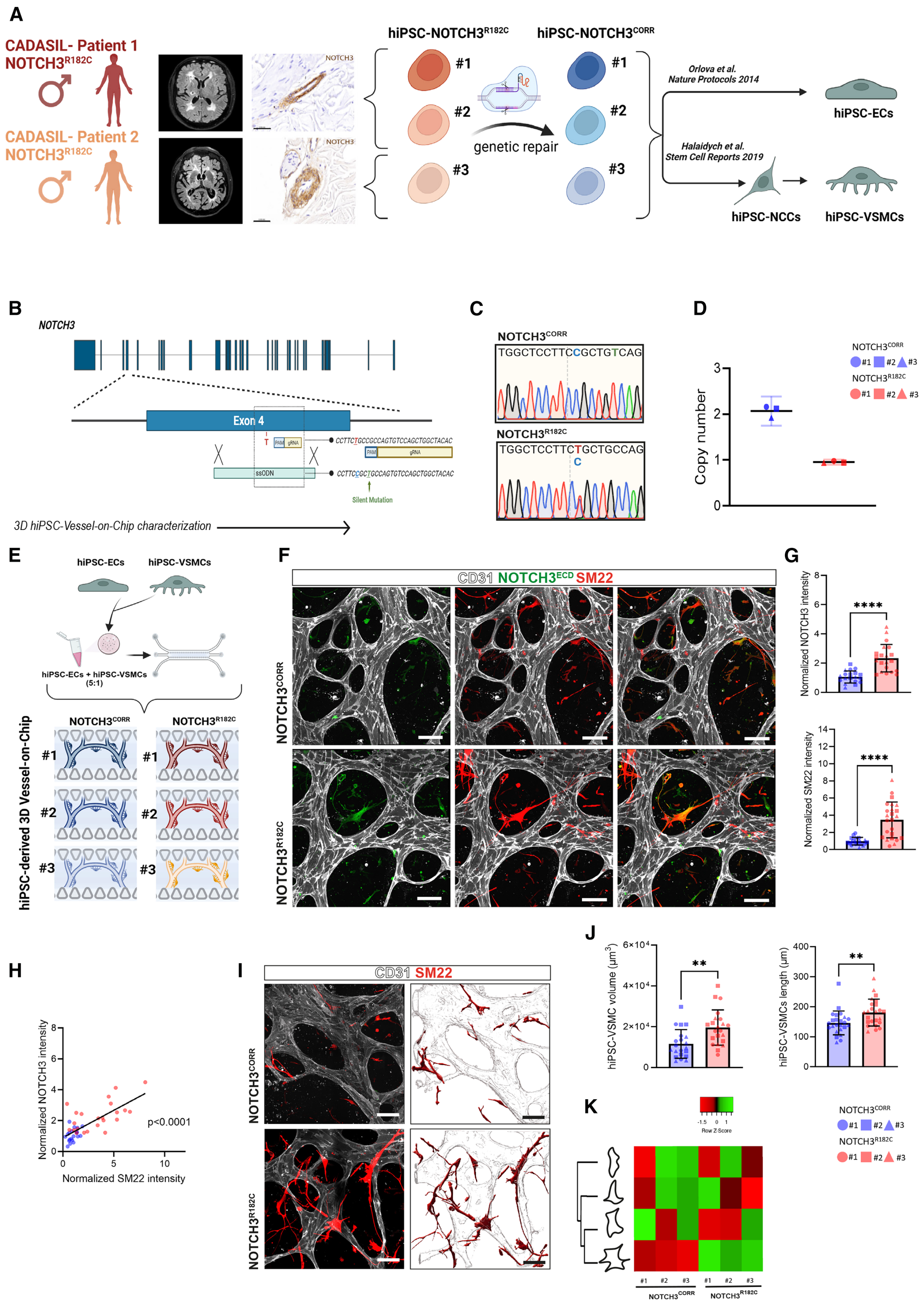

(legend on next page)

(Figures 1I and 1J). This indicated that CADASIL VSMCs exhibit increased NOTCH3^{ECD} protein levels and undergo phenotypic changes, which are linked to alterations in vessel morphology but only in the 3D Vessel-on-Chip model.

CADASIL hiPSC-VSMCs show reduced maturation in 2D with no changes in NOTCH3^{ECD} levels

Since primary VSMCs are difficult to obtain from postmortem patients and it is essential to whether findings are reproducible across patients, we investigated whether the same phenotypic changes were evident in hiPSC-derived VSMCs from CADASIL patients. We first generated three hiPSC clones from two CADASIL patients with a *NOTCH3* c.544C>T; p.Arg182Cys variant (NOTCH3^{R182C}) (Figure 2A). We used CRISPR-Cas9 to correct the *NOTCH3* c.544C>T variant to develop line-specific isogenic controls (NOTCH3^{CORR}, Figure 2B). We subcloned two hiPSC lines for Patient 1 and one subclone for Patient 2, each paired with its respective genetically repaired isogenic control. Results presented are averages of data from these three subclones. We confirmed the correction of the NOTCH3^{R182C} variant by Sanger sequencing and that both alleles were gene edited by digital droplet PCR-based allelic drop-off assay (Figures 2C and 2D). Both the CADASIL and isogenic corrected hiPSC lines showed a normal karyotype by G-banding and exhibited typical pluripotency features, such as the expression of OCT4, TRA 1–60, and NANOG, and multi-lineage differentiation potential (Figures S1A–S1C). We generated hiPSC-derived VSMCs via neural crest (NC) intermediates as previously described (Halaidych et al., 2019), as these are the primary source of VSMCs in the cerebral vasculature (Majesky 2007). We also generated hiPSC-ECs using established protocols (Orlova et al., 2014; Orlova, van den Hil et al., 2014). Unexpectedly, NOTCH3^{R182C} and NOTCH3^{CORR} hiPSCs showed different NC differentiation dynamics with NOTCH3^{R182C} hiPSC-NC cells showing reduced CD271^{+} and increased SOX2^{+} populations at initial stages (p0) of NC differentiation (Figures S2A and S2B). However, NC cell (NCC) derivation efficiencies were comparable by p3 (Figures S2A and S2B). Bulk RNA sequencing confirmed that both NOTCH3^{CORR} and NOTCH3^{R182C} hiPSC clones differentiated into hiPSC-derived ECs, NCCs, and VSMCs, with minimal variance as shown by principal-component analysis (PCA, Figure S2C). We observed that 23.1% of the total variation within the data was due to differences between both patients and continued with cells derived from Patient 1 for downstream analysis (Figure S2C). We next identified 401 differentially expressed genes between NOTCH3^{CORR} and NOTCH3^{R182C} hiPSC-VSMCs (Figure S2D; Table S1). Pathway analysis indicated enrichment of pathways related to extracellular matrix organization and degradation in hiPSC-VSMC NOTCH3^{R182C} (Figure S2E). Immunofluorescence 2D analysis showed no difference in NOTCH3^{ECD} levels between NOTCH3^{R182C} and NOTCH3^{CORR} hiPSC-VSMCs (Figures S2F and S2G). Further, 2D characterization of hiPSC-VSMC revealed a significant reduction in the contractile protein SM22 in NOTCH3^{R182C} hiPSC-VSMCs and changes in cell morphology, evidenced by a reduction in

Figure 2. CADASIL hiPSC-VSMCs recapitulate increased NOTCH3 levels and VSMC alterations in 3D Vessel-on-Chip
(A) Schematic of study design. Three clones of hiPSC-NOTCH3^{R182C} were generated from two CADASIL patient lines, and gene-corrected lines (hiPSC-NOTCH3^{CORR}) were generated using CRISPR-Cas9. Brain MRI of both patients showed extensive confluent white matter hyperintensities and multiple lacunes. NOTCH3 immunohistochemistry of skin vessels showed pathognomonic NOTCH3 protein accumulation in both patients. Scale bars, 50 μm. hiPSC lines were differentiated toward ECs and VSMCs via NC intermediates using established protocols.
(B) Schematic overview of the targeting strategy to correct the heterozygous c.544C>T;p.Arg182Cys pathogenic variant in the patient-derived hiPSC lines (hiPSC-NOTCH3^{R182C}). Double-stranded breaks were introduced in the genome guided by guide RNAs in NOTCH3 exon 4. A repair template was used for homologous recombination (ssODN) with the corrected allele at position 544 and with a silent variant into the PAM.
(C) Sanger sequencing reads of *NOTCH3* showing the heterozygous c.544C>T; p.Arg182Cys variant in the patient-derived hiPSC lines (NOTCH3^{R182C}) and the correction of the variant and presence of the silent variant in the PAM in isogenic control lines (NOTCH3^{CORR}).
(D) Graph showing copy number variation of the corrected allele.
(E) Schematic representation of 3D Vessel-on-chip experiments with hiPSC-derived vascular cells. hiPSC-VSMCs NOTCH3^{R182C} and hiPSC-VSMCs NOTCH3^{CORR} were cultured with hiPSC-ECs in microfluidic devices.
(F) Representative confocal images of the microvascular network showing hiPSC-ECs (gray; CD31) and hiPSC-VSMCs (green; NOTCH3, red; SM22). Magnification: 40×, scale bars, 100 μm.
(G and H) Quantification of the normalized intensities of NOTCH3 and SM22 in hiPSC-VSMCs (G) and respective Spearman correlation (H).
(I) Representative confocal images of microvascular network showing in hiPSC-ECs (gray; CD31) and hiPSC-VSMCs (red; SM22). Magnification: 40×, scale bars, 100 μm.
(J and K) Quantification of hiPSC-VSMC volume (μm^3, J) and length (μm, J) and heatmap comparing the abundance of hiPSC-VSMCs falling into four different cell shapes (K).
Data are from $N = 4$ four independent experiments and shown as ± SD. Unpaired t test. ****$p < 0.0001$, **$p < 0.005$, ns, not significant.

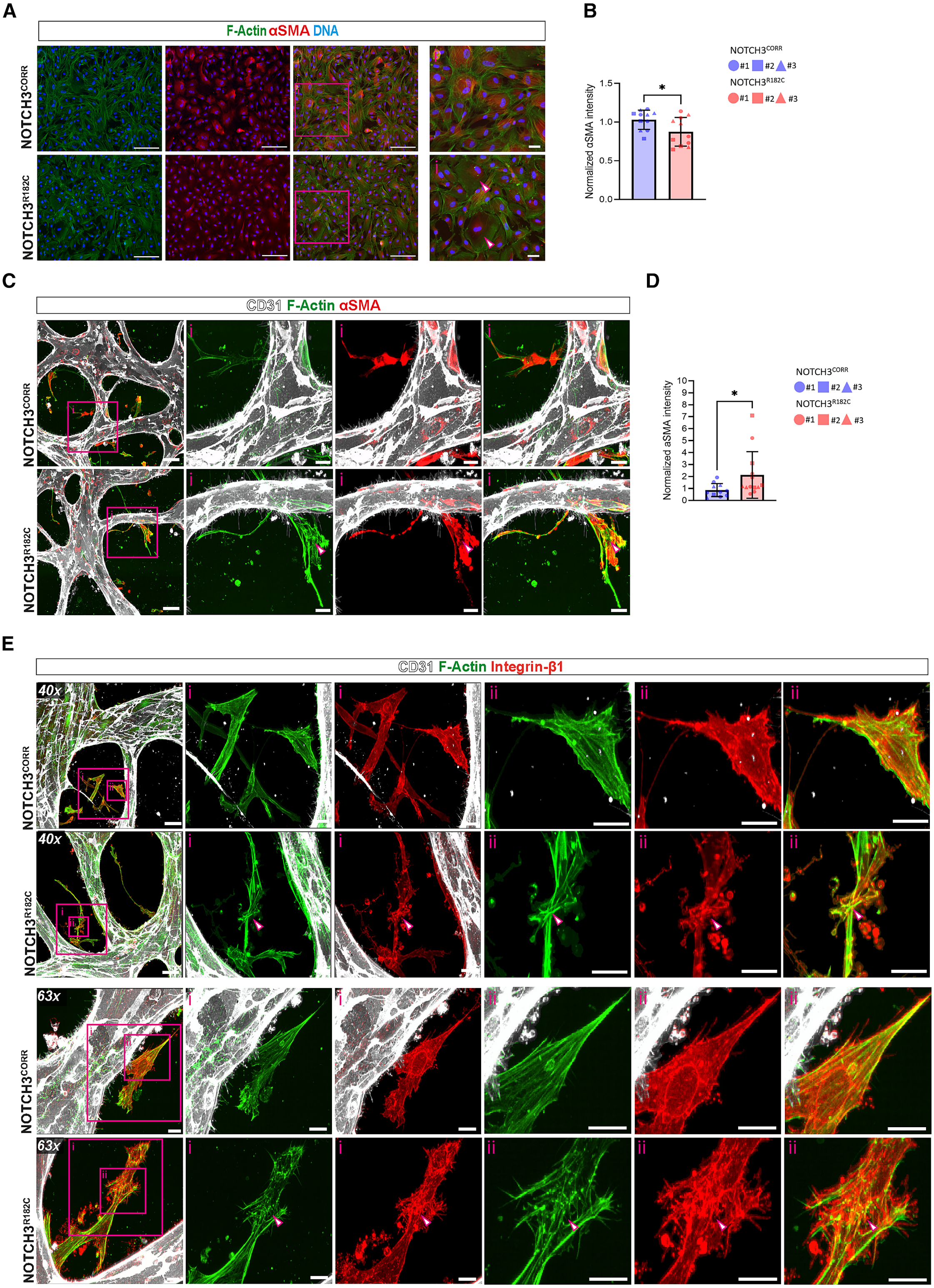

Figure 3. CADASIL hiPSC-VSMCs show disrupted actin cytoskeleton organization and integrin β1 focal adhesions in 3D Vessel-on-Chip
(A) Representative immunofluorescence images showing expression of F-actin (green), αSMA (red), and nuclei (blue) of hiPSC-VSMCs. Magnification: 10×, scale bars, 250 μm. Enlargement (i): arrows indicate less stress F-actin fiber distribution in NOTCH3^{R182C} hiPSC-VSMCs compared to NOTCH3^{CORR} hiPSC-VSMCs, scale bars, 50 μm.

(legend continued on next page)

VSMC surface area compared to isogenic controls (Figures S2F and S2G).

hiPSC-derived CADASIL VSMCs recapitulate primary CADASIL VSMC features in 3D Vessel-on-Chip

We next incorporated either NOTCH3^{R182C} or NOTCH3^{CORR} hiPSC-VSMCs along with NOTCH3^{CORR} hiPSC-ECs into the 3D Vessel-on-Chip (Figure 2E). Both types of hiPSC-VSMCs supported the formation of microvascular networks and luminized vessels in microfluidic chips after 7 days of co-culture (Figures S3A and S3B). In contrast to primary CADASIL brain VSMCs, no significant differences in microvascular network parameters were observed between the groups (Figure S3C). Immunofluorescence analysis revealed that NOTCH3^{R182C} hiPSC-VSMCs exhibited increased levels of NOTCH3 protein in the 3D Vessel-on-Chip, like CADASIL primary VSMCs (Figures 2F and 2G). Additionally, an increase in SM22 contractile protein was observed in NOTCH3^{R182C} hiPSC-VSMCs, which was correlated with the increased NOTCH3 protein levels (Figures 2F–2H). Quantification of the morphological parameters of hiPSC-VSMCs revealed striking differences in NOTCH3^{R182C} hiPSC-VSMCs compared to isogenic counterparts, like those observed with CADASIL primary VSMCs (Figure 2I; Video S1). Specifically, NOTCH3^{R182C} hiPSC-VSMCs exhibited a larger volume and a greater number of protrusions, as indicated by more stellate-shaped cells (Figures 2I–2K), while NOTCH3^{CORR} hiPSC-VSMCs displayed a more spindle-like morphology (Figure 2K). In 2D cultures, NOTCH3^{R182C} hiPSC-VSMCs exhibited fewer and less-prominent actin stress fibers, together with significantly lower intensity of other contractile proteins such as αSMA (ACTA2 gene product; Figures 3A and 3B). In contrast, when incorporated in the 3D Vessel-on-Chip, NOTCH3^{R182C} hiPSC-VSMCs displayed an abnormal cytoskeletal architecture characterized by highly dense actin bundles within the VSMC protrusions and disrupted patterns forming node-like structures (Figure 3C). Consistent with changes in SM22, αSMA protein levels were also significantly increased in the 3D Vessel-on-Chip (Figures 3C and 3D). In addition, we observed altered patterns in integrin β1-associated focal adhesion in NOTCH3^{R182C} hiPSC-VSMCs, which appeared abnormally clustered within the disrupted node-like actin structures, whereas NOTCH3^{CORR} hiPSC-VSMCs showed focal adhesions predominantly localized at cellular extensions (Figure 3E). This suggested that the increase of NOTCH3 in CADASIL is related to phenotypic changes in hiPSC-VSMCs, mirroring those observed in primary brain VSMCs from CADASIL patients cultured in the 3D Vessel-on-Chip.

PDGFRβ protein levels correlate with NOTCH3^{ECD} levels in CADASIL 3D Vessel-on-Chip and patient tissue

PDGFRβ is a direct downstream target of NOTCH3 (Jin et al., 2008), and the interaction between NOTCH3 and PDGFRβ plays a critical role in maintaining VSMC function and vascular homeostasis by regulating pathways involved in cell proliferation, migration, survival, and differentiation (Domenga et al., 2004). Immunofluorescence analysis in the 3D Vessel-on-Chip showed an increase in PDGFRβ intensity in both NOTCH3^{R153C} primary VSMCs and NOTCH3^{R182C} hiPSC-VSMCs compared to their respective controls (Figures 4A–4C). Additionally, PDGFRβ levels were positively correlated with NOTCH3^{ECD} protein levels (Figure 4D). We next investigated whether the observed changes in protein levels were attributable to increased mRNA levels of NOTCH3 and its downstream target genes. Using a Jagged1-bead activation assay in 2D hiPSC-VSMCs (Figure S4A) (Zohorsky et al. 2021), we showed that the NOTCH3^{R182C} variant did not significantly alter the expression of *NOTCH3* or its downstream target genes (Figure S4B). Similarly, in the 3D Vessel-on-Chip model, we found no significant differences in mRNA levels of *NOTCH3* or *PDGFRβ* or other *NOTCH3* downstream target genes (Figure S4C), or in the EC-specific gene *PECAM1* or the VSMC-specific gene TAGLN (Figure S4C). We next investigated the relationship between NOTCH3 and PDGFRβ protein levels in brain blood vessels from CADASIL patients (n = 12; Table S2), by immunohistochemical staining of brain blood vessels from CADASIL patients and controls, as previously described (Figure 4E) (Gravesteijn et al., 2022; Rutten et al., 2015). We found a significant positive correlation in each patient between NOTCH3 and PDGFRβ

(B) Quantification of intensity of normalized αSMA intensity hiPSC-VSMCs.

(C) Representative confocal images of microvascular network showing hiPSC-ECs (gray; CD31) and F-actin (green) and αSMA (red). Magnification: 40×, scale bars, 100 μm. Enlargement (i): arrows indicate F-actin node organization in NOTCH3^{R182C} hiPSC-VSMCs, scale bars, 20 μm.

(D) Quantification of intensity of normalized αSMA intensity in hiPSC-VSMCs.

(E) Representative confocal images of microvascular network showing hiPSC-ECs (gray; CD31), F-actin (green), and integrin β1 focal adhesions (red). Magnification: 40×, scale bars, 50 μm. Enlargement (i and ii): arrows indicate disrupted patterns of F-actin and integrin β1 in NOTCH3^{R182C} hiPSC-VSMCs, scale bars, 20 μm. Magnification: 63×, scale bars, 10 μm. Enlargement (i and ii): arrows indicate disrupted patterns of F-actin and integrin β1 in NOTCH3^{R182C} hiPSC-VSMCs, scale bars, 10 μm.

Data are from N = 3 three independent experiments and shown as ±SD. Unpaired t test. *p < 0.05, ns.

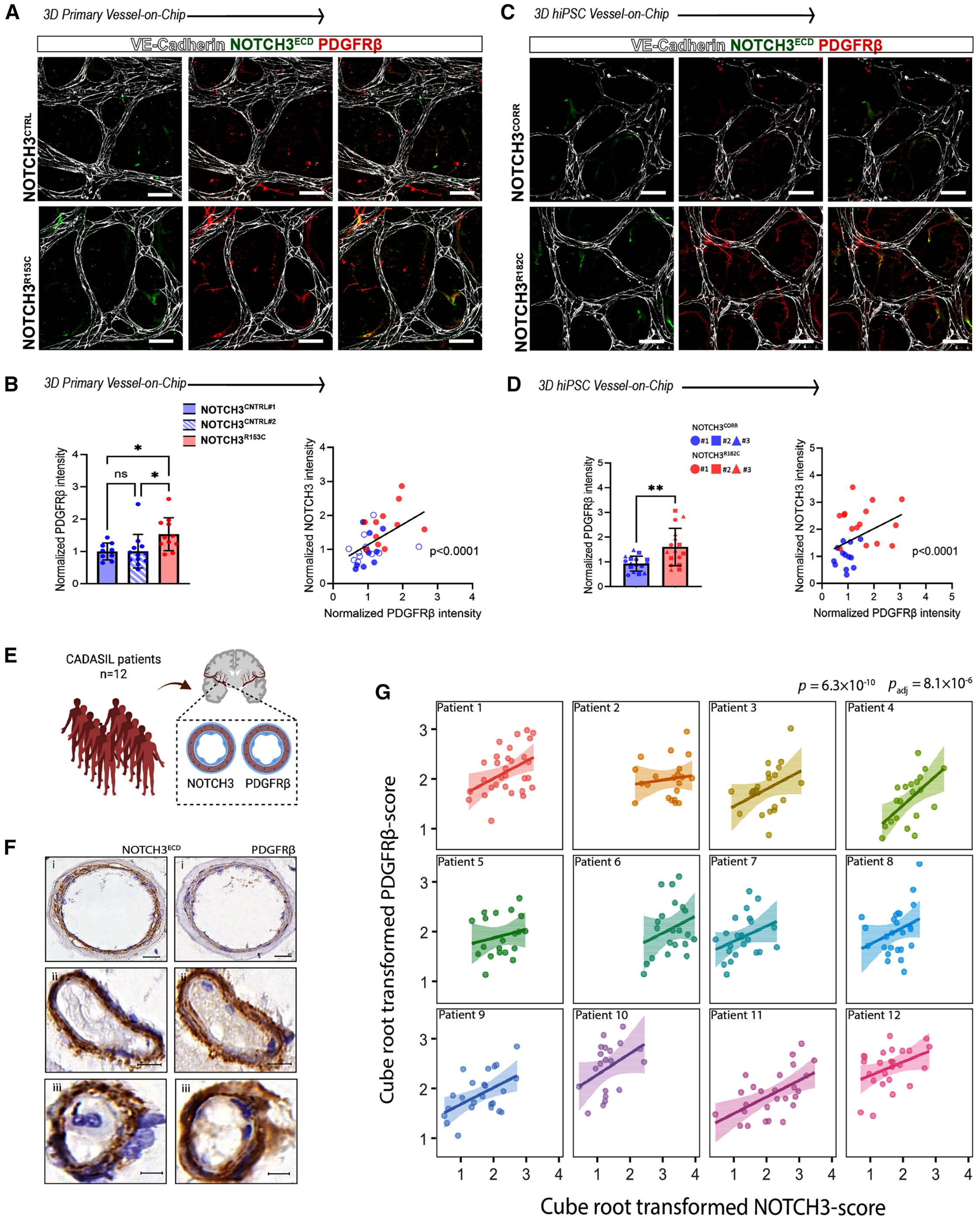

Figure 4. CADASIL VSMCs show increased PDGFRβ protein levels correlating with NOTCH3 protein levels in 3D Vessel-on-Chip and in patient brain tissue

(A) Representative confocal images of microvascular network showing hiPSC-ECs (gray; CD31) and primary VSMCs (green; NOTCH3, red; PDGFRβ). Magnification: 40×, scale bars, 100 μm.

(B) Quantification of normalized PDGFRβ intensity and Spearman correlation between normalized NOTCH3 and PDGFRβ intensities in primary VSMCs.

(C) Representative confocal images of microvascular network showing hiPSC-ECs (gray; CD31) and hiPSC-VSMCs (green; NOTCH3, red; PDGFRβ). Magnification: 40×, scale bars 100 μm.

(legend continued on next page)

protein levels in CADASIL patient vessels ($n = 12$) (Figures 4F and 4G). This indicated correlation between PDGFRβ and $NOTCH3^{ECD}$ levels in the 3D Vessel-on-Chip model, which reflect that in patient brain tissue.

CADASIL hiPSC-derived VSMCs are functionally deficient in 3D Vessel-on-Chip

We next explored whether $NOTCH3^{R182C}$ affects the functional properties of VSMCs. In 2D functional assays, $NOTCH3^{R182C}$ hiPSC-VSMCs exhibited reduced contractility and prolonged Ca^{2+} waves in response to mechanical pressure, while stimulation with endothelin-1 (ET-I) induced similar responses in $NOTCH3^{R182C}$ and $NOTCH3^{CORR}$ hiPSC-VSMCs (Figures 5A and 5B). To assess functional changes in the 3D Vessel-on-Chip model, we engineered $NOTCH3^{R182C}$ and $NOTCH3^{CORR}$ hiPSC-VSMCs from Patient 2 such that they expressed an ultra-sensitive Ca^{2+} sensor (GCaMP6f) (Chen et al., 2013) as previously described (Vila Cuenca et al., 2021). Next, we determined the cytosolic Ca^{2+} release in the 3D Vessel-on-Chip on day 7 (Figure 5C; Video S2). Before ET-I perfusion (time 0 s), $NOTCH3^{R182C}$ hiPSC-VSMCs showed significantly higher basal fluorescence intensity compared to $NOTCH3^{CORR}$ hiPSC-VSMCs (Figures 5D and 5E). At 50 s after ET-I stimulation, fluorescence intensity was comparable between both groups, indicating that $NOTCH3^{R182C}$ and $NOTCH3^{CORR}$ hiPSC-VSMCs showed a similar response to ET-I stimulation during the main peak ([MP] Figures 5F and 5G). At 300 s, the fluorescence intensity of $NOTCH3^{R182C}$ hiPSC-VSMCs remained elevated, while that of $NOTCH3^{CORR}$ hiPSC-VSMCs had returned to baseline, reflecting differences in the secondary peak (SP) response (Figures 5F and 5G). Comparison of kinetics of the Ca^{2+} responses showed no differences between the groups in the time parameters during the MP (Figure 5H), whereas $NOTCH3^{R182C}$ hiPSC-VSMCs displayed an increased duration and time to peak but no changes in the decay during the SP response (Figure 5I). Together, these data demonstrate that $NOTCH3^{R182C}$ hiPSC-VSMCs exhibit abnormal Ca^{2+} responses in the 3D Vessel-on-Chip model, validating its utility to uncover functional differences between normal and CADASIL hiPSC-VSMCs.

Inhibition of NOTCH3 cleavage rescues CADASIL hiPSC-VSMC phenotypic and functional changes in 3D Vessel-on-Chip model

The γ-secretase inhibitor DAPT has been shown to block the *in vitro* and *in vivo* cleavage and signaling of NOTCH proteins (Li et al., 2009; Hellstrom et al., 2007). We, therefore, hypothesized that inhibiting γ-secretase could mitigate the effects of pathogenic *NOTCH3* variants in VSMCs within the 3D Vessel-on-Chip model. After 48 h of DAPT treatment, $NOTCH3^{R182C}$ hiPSC-VSMCs showed reduced $NOTCH3^{ECD}$ and SM22 protein levels, as well as a reduction in cell volume, resembling their untreated $NOTCH3^{CORR}$ hiPSC-VSMCs counterparts (Figures 6A and 6B). Additionally, DAPT treatment normalized Ca^{2+} responses in $NOTCH3^{R182C}$ hiPSC-VSMCs (Figure 6C), particularly at 300 s after ET-I stimulation, as well as the response kinetics of the SP response in $NOTCH3^{R182C}$ hiPSC-VSMCs (Figures 6D and 6E). These results indicate that lowering $NOTCH3^{ECD}$ levels by γ-secretase inhibition restores the morphological and functional changes in CADASIL hiPSC-VSMCs.

DISCUSSION

In this study, we developed a multi-cell type CADASIL 3D Vessel-on-Chip model that has disease-relevant morphological and functional readouts in VSMCs. The results from the model were similar to those obtained using patient-derived primary VSMCs, hiPSC-derived VSMCs, and their isogenic corrected controls, providing a robust *in vitro* platform for studying early pathological changes in VSMCs and testing of therapeutic compounds.

CADASIL-causing *NOTCH3* variants lead to aggregation and accumulation of $NOTCH3^{ECD}$ around VSMCs with sequestration of extracellular matrix proteins, resulting in VSMC degeneration and vessel wall dysfunction (Joutel et al., 2000). We found increased $NOTCH3^{ECD}$ protein in both CADASIL primary and hiPSC-derived VSMCs after 7 days of co-culture in the 3D Vessel-on-Chip model. Notably, this was not observed in conventional 2D assays, highlighting the importance of the 3D environment for recapitulating the interaction between ECs and VSMC.

(D) Quantification of normalized PDGFRβ intensity and Spearman correlation between normalized NOTCH3 and PDGFRβ intensities in hiPSC-VSMCs.
(E) Schematic representation of CADASIL patient immunohistochemistry.
(F) Representative brain blood vessels from CADASIL patients with different sizes showing NOTCH3 aggregates and PDGFRβ protein in the blood vessel wall. Scale bars, 20 (i), 10 (ii), and 5 (iii) μm.
(G) Correlation analysis between cube root-transformed PDGFRβ and NOTCH3 score from CADASIL patients ($n = 12$). Each plot represents one individual, and each dot a blood vessel measurement.
Data are from $N = 3$ three independent experiments and shown as ± SD. One-way ANOVA test (B) and t test (D). $^{**}p < 0.005$, $^{*}p < 0.05$, ns, not significant.

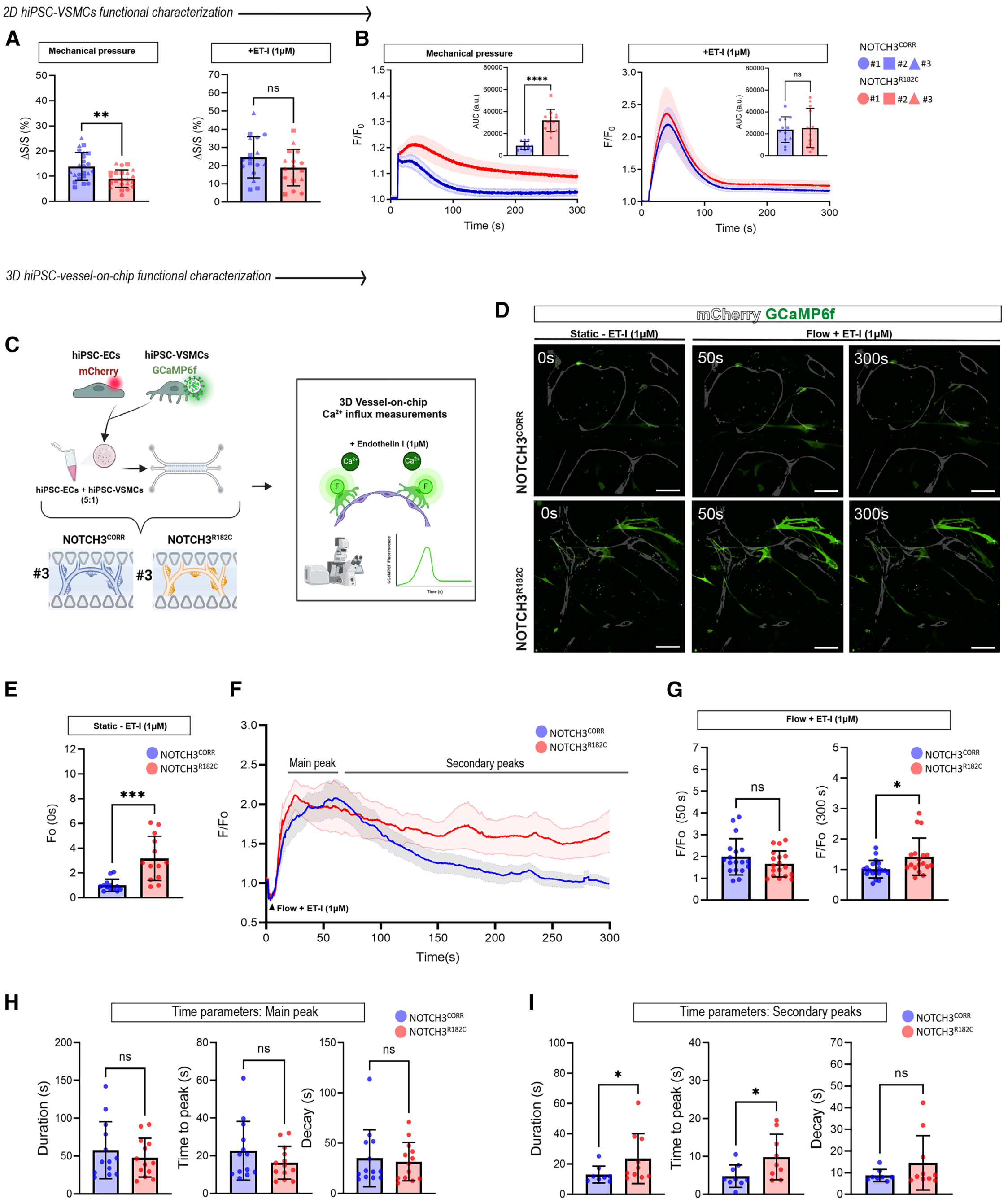

Figure 5. CADASIL hiPSC-VSMCs show altered Ca^{2+} dynamics in 3D Vessel-on-Chip

(A and B) 2D functional characterization of hiPSC-VSMCs. Relative cell surface area decrease upon mechanical stimulation (medium refreshment) and ET-I stimulation (A). Normalized average fluorescence intensity F/F_0 in hiPSC-VSMCs upon mechanical stimulation (medium refreshment) and ET-I stimulation (B). Inserts show quantifications of the AUC.

(C) Schematic of the functional 3D Vessel-on-Chip study using hiPSC-VSMCs engineered to express an ultra-sensitive Ca^{2+} sensor (GCaMP6f) cultured with fluorescently tagged (mCherry) healthy control hiPSC-ECs.

(D) Representative confocal images of intracellular Ca^{2+} fluorescence showing hiPSC-ECs (gray; mCherry) and hiPSC-VSMCs (green; GCaMP6f) at different time points (0, 50, and 300 s) after ET-I stimulation. Magnification: 20×, scale bars, 100 µm.

(E) Fluorescence intensity at time (F_0, 0 s) of hiPSC-VSMCs cultured in 3D Vessel-on-chip under static conditions.

(F) Normalized average fluorescence intensity F/F_0 in hiPSC-VSMCs expressing GCaMP6f. Stimulation time point is set at 5 s.

(legend continued on next page)

A similar conclusion was drawn in a previous study on the microvascular disease hereditary hemorrhagic telangiectasia (Orlova et al., 2022).

NOTCH3 regulates the expression of contractile VSMC markers and is critical for the differentiation and maintenance of the contractile VSMC phenotype (Henshall et al., 2015). We found that both primary and hiPSC-derived CADASIL VSMCs exhibited contractile-like morphologic changes in a 3D context, including elongated morphology, larger size, and more protrusions (Liu et al. 2009; Henshall et al., 2015). These alterations were accompanied by increased levels of the contractile proteins SM22 and αSMA, as well as disrupted actin cytoskeleton organization and altered integrin β1-associated focal adhesions. Such abnormalities in actin cytoskeleton organization and focal adhesion structure have also been reported in previous studies using primary and hiPSC-derived CADASIL VSMCs (Tikka et al., 2012; Ling et al., 2019), supporting the notion that cytoskeletal and adhesion defects are consistent and characteristic features of CADASIL VSMCs. Here, we observed a strong correlation between $NOTCH3^{ECD}$ and SM22 levels in CADASIL VSMCs, suggesting that pathogenic *NOTCH3* variants drive the acquisition of a contractile phenotype in these cells (van Splunder et al., 2024; Gatti et al., 2018). This aligns with previous findings that NOTCH3 is essential for maintaining vascular contractility, as *Notch3*-deficient mice exhibit cerebrovascular dysfunction due to the loss of VSMC contractility (Romay et al., 2024; Domenga et al., 2004). In the present study, we also observed a strong correlation between PDGFRβ and $NOTCH3^{ECD}$ protein levels in the 3D Vessel-on-Chip model, a relationship further confirmed in brain blood vessels from CADASIL patients. The role of PDGFRβ in CADASIL has been inconclusive, with contradictory results from studies using 2D models (Tikka et al., 2012; Kelleher et al., 2019; Jin et al., 2008). An increase in PDGFRβ levels in CADASIL patient brain arteries has been previously found (Craggs et al., 2015; Littau et al., 2022), although a direct relationship with $NOTCH3^{ECD}$ aggregates has not been described. Moreover, studies in mice have shown that constitutive activation of PDGFRβ increases mural cell coverage, promotes a pro-inflammatory profile, and enhances the synthesis of extracellular matrix proteins, providing an additional link between PDGFRβ and vessel wall pathology (Olson and Soriano 2011; He et al., 2015).

Recent studies with hiPSC-derived CADASIL vascular cells showed that VSMCs fail to stabilize tubular structures in a Matrigel cord-forming assay (Kelleher et al., 2019). In this study, vascular defects, including a reduction of vessel diameter were only induced by primary CADASIL VSMCs in the 3D Vessel-on-Chip model. This discrepancy between primary and hiPSC-derived VSMCs may be attributable to differences in cellular maturity and/or overall behavior when VSMCs are cultured in the 3D Vessel-on-Chip, as we previously showed (Vila Cuenca et al., 2021; Halaidych et al., 2019).

NOTCH3 autoregulates its own expression through a positive feedback loop and modulates downstream target genes upon contact with ECs (Liu et al. 2009). Although a long-standing debate has been whether increased or reduced NOTCH3 signaling contributes to CADASIL vascular pathology, recent work in Tg*Notch3*^{R169C} mice arteries showed no changes in mRNA expression of Notch3 downstream target genes (Dupre et al., 2024). Consistent with this, we recently demonstrated that although different NOTCH3 risk categories are strongly associated with distinct levels of vascular $NOTCH3^{ECD}$ aggregation load in patient brain and skin vessels, NOTCH3 signaling activity itself does not differ across those categories (Hack et al., 2023). In the present study, we similarly observed no substantial changes in mRNA levels of *NOTCH3*, contractile proteins, or NOTCH3 downstream targets such as *HEY1*, *HES1*, and *PDGFRB* in either the 2D Jagged1-bead activation assay or the 3D Vessel-on-Chip model. Together, these findings further support the now widely accepted hypothesis that CADASIL pathology is primarily driven by the accumulation of NOTCH3 protein.

NOTCH3 is a key regulator of vascular tone in small arteries and of vascular reactivity in response to mechanical factors such as pressure and flow (Belin de Chantemele et al., 2008; Hussain et al., 2004). Notably, *Notch3* deficiency in mice has been associated with the disruption of proteins involved in Ca^{2+} dynamics (Romay et al., 2024), a key factor in vascular smooth muscle contraction, which is triggered by an increase in cytosolic free Ca^{2+} concentration (Touyz et al., 2018). In the present study, CADASIL hiPSC-VSMCs exhibited abnormal contractile behavior and disrupted Ca^{2+} dynamics in 2D cultures when subjected to mechanical pressure, but not in response to the vasoactive compound ET-I. In contrast, functional assays using the 3D Vessel-on-Chip model revealed prolonged Ca^{2+} responses in CADASIL hiPSC-VSMCs upon flow perfusion with ET-I-supplemented medium. The observed phenotypic discrepancies, together with differences in contractile protein expression, may reflect distinct maturation states of hiPSC-VSMCs in 2D and 3D culture

(G–I) Ca^{2+} transient parameters: average fluorescence intensity F/F_0 50 s and 300 s (G). Duration (s), time to peak (s), and decay (s) of the MP (H) and SP (I).
Data are from $N = 3$ three independent experiments and shown as ±SD. Unpaired *t* test. $^{****}p < 0.0001$, $^{***}p < 0.001$, $^{**}p < 0.01$, $^{*}p < 0.05$, ns, not significant.

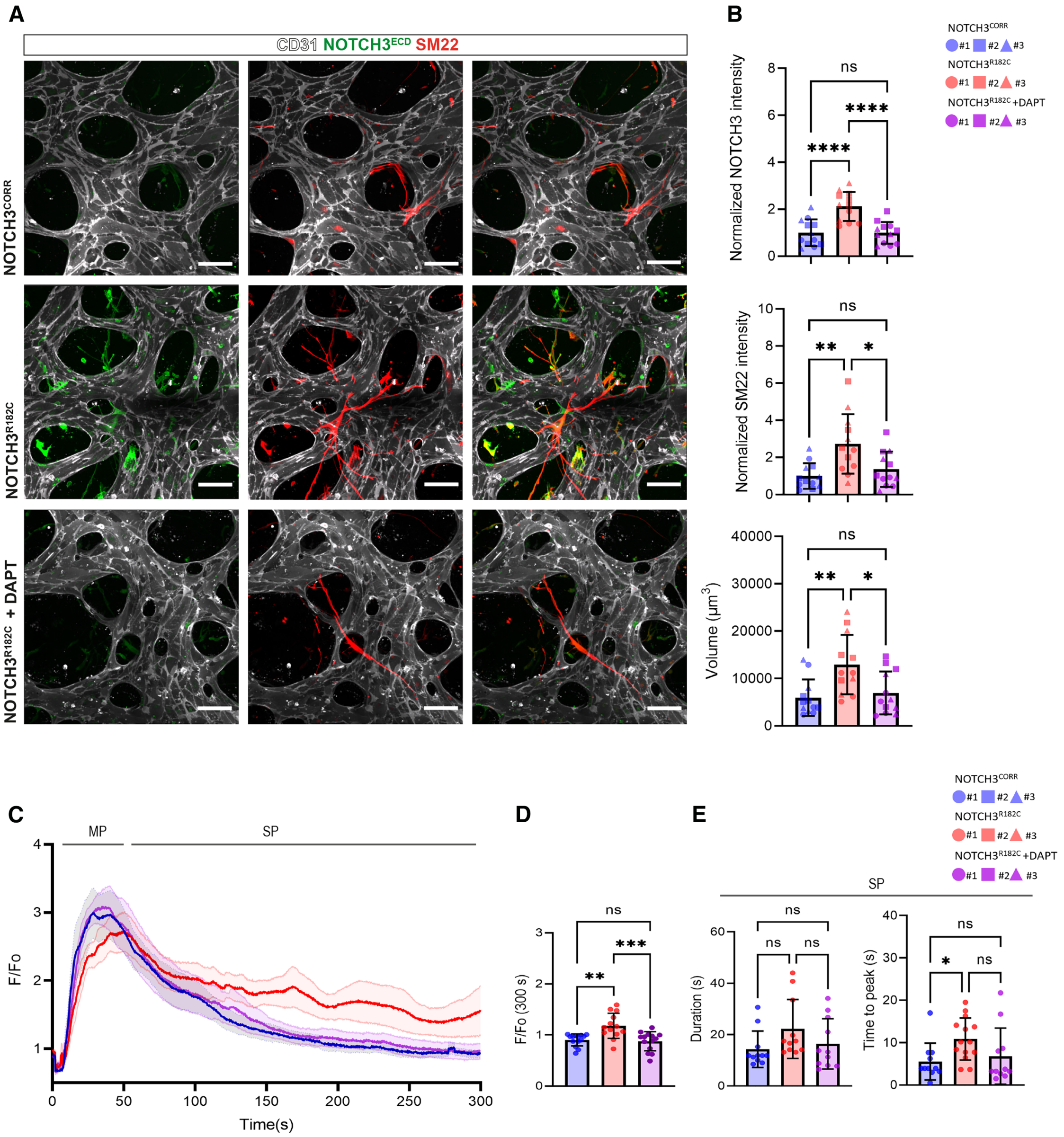

Figure 6. Inhibition of NOTCH3 cleavage rescues VSMC phenotypic and functional changes in hiPSC-derived 3D Vessel-on-Chip

(A) Representative confocal images of microvascular network showing in hiPSC-ECs (gray; CD31) with hiPSC-VSMCs (NOTCH3, green; SM22, red). Magnification: 40×, scale bars, 100 μm.

(B) Quantification of normalized intensity of NOTCH3, SM22, and volume (μm^3) in hiPSC-VSMCs.

(C) Normalized average fluorescence intensity F/F_0 in hiPSC-VSMCs expressing GCaMP6f. Stimulation time point is set as t = 5 (s).

(D and E) Ca^{2+} transient parameters: Average fluorescence intensity F/F_0 at time 300 s (D). Duration (s) and time to peak (s) and decay (s) of the SP (E).

Data are from $N = 3$ three independent experiments and shown as ± SD. One-way ANOVA test. ****$p < 0.0001$, ***$p < 0.001$, **$p < 0.005$, *$p < 0.05$, ns, not significant.

conditions. The 3D Vessel-on-Chip system allows direct contact with hiPSC-ECs, which may promote a more mature and contractile VSMC phenotype and facilitate the emergence of CADASIL-relevant phenotypes (Cerneckis et al. 2024). Together, these findings indicate that CADASIL VSMCs exhibit a hyperactivated contractile state.

Proteolytic cleavage of NOTCH receptors is mediated by γ-secretase protein complexes, and its inhibition been shown to effectively prevent NOTCH3 S3 cleavage (Li

et al., 2009; Wimmer et al., 2019). In CADASIL, ligand binding followed by NOTCH3 receptor cleavage has been proposed as a critical event driving NOTCH3^{ECD} accumulation at the VSMC surface. In our study, γ-secretase inhibition alleviated CADASIL VSMC phenotypic and functional alterations, suggesting that NOTCH3 cleavage has a restorative effect on CADASIL VSMC phenotype. We acknowledge, however, that we cannot fully exclude potential effects on NOTCH3 intracellular domain release or broader signaling alterations, even though our data show that NOTCH3^{R182C} variant does not significantly affect downstream target gene expression. Additionally, γ-secretase mediates proteolysis of other transmembrane proteins beyond NOTCH receptors (Ni et al., 2001). Therefore, although we observe a reduction of NOTCH3^{ECD} protein levels in CADASIL VSMCs upon γ-secretase inhibition, we cannot fully attribute the observed phenotypic changes solely to NOTCH3 cleavage inhibition.

Several questions remain unanswered and limit the extent of our study's conclusions. Although we observed an increase of NOTCH3^{ECD} protein abundance in VSMCs in 3D Vessel-on-Chip, we did not observe NOTCH3^{ECD} aggregation, a hallmark of CADASIL vessel pathology. The 3D Vessel-on-Chip model employed here does not fully recapitulate the complex cellular composition of the cerebral vasculature, which might be critical for the formation of NOTCH3^{ECD} aggregates. A more complex model incorporating neuronal cell types, extending cell culture conditions, and/or introducing a constant flow could potentially address this issue, but lies beyond the scope of the current study. Finally, while blocking NOTCH3 proteolytic cleavage using the γ-secretase inhibitor DAPT rescued the phenotype in CADASIL VSMC, the broad substrate specificity of γ-secretase limits its potential clinical application.

In summary, we generated a robust CADASIL 3D Vessel-on-Chip model that shows reproducible results for both primary and hiPSC-derived CADASIL patient VSMCs including (1) increased abundance of NOTCH3^{ECD} protein; (2) morphological alterations accompanied by disrupted actin organization, abnormal focal adhesions, and increased expression of contractile and PDGFRβ proteins; as well as (3) functional alterations in calcium dynamics. Finally, we demonstrate the utility of the model for drug testing with a proof-of-concept rescue using the γ-secretase inhibitor DAPT.

METHODS

hiPSC lines

Research on hiPSC was approved by the Medical Ethical Committee (P13.080) at Leiden University Medical Center, the Netherlands, and written informed consent was obtained from all patients. Erythroblasts isolated from peripheral blood were used for reprogramming as described previously (Bouma et al. 2017, 2020). The following hiPSC lines were generated from Patient 1: LUMC0169iNOTCH and Patient 2: LUMC0194iNOTCH. The heterozygous *NOTCH3* c.544C>T variant located in exon 4 was corrected by insertion of a single base (C) and simultaneous introduction of two silent variants using CRISPR-Cas9-induced homology-directed repair. The targeting strategy is depicted in Figure 2A. Detailed description of CRISPR gene correction strategy and hiPSC line maintenance and characterization can be found in the supplemental information.

Setting up 3D Vessel-on-Chip culture

hiPSC differentiation toward ECs and VSMCs and 2D characterization is described in the supplemental information. Cell preparation and chip setting up was performed as described previously (Vila Cuenca et al., 2021). Commercially available microfluidic chips with one gel channel and two media channels (AIM Biotech, IdenTx 9) were used. Cells were resuspended and combined to obtain 10×10^6 hiPSC-ECs/mL and 2×10^6 VSMCs/mL (5:1 ratio). Cells were resuspended in EGM-2 supplemented with Thrombin (4 U/mL) and then gently mixed with fibrinogen (final concentration 3 mg/mL, Sigma) at 1:1 vol ratio. Cell/hydrogel mixture was quickly loaded into the middle gel-loading channel of the microfluidic chip. Chips were incubated at room temperature for 15 min before the addition of EGM-2 supplemented with VEGF (50 ng/mL) to both flanking media channels. The γ-secretase inhibitor DAPT (10 μM) was also added to the medium on day 1 for 24 h. Gravity-driven flow was induced by the addition of 100 μL medium to the right media ports and 50 μL media to left media ports. Medium was refreshed daily.

CADASIL patient brain tissue staining and quantifications

In all CADASIL patients (n = 12, Table S2), tissue samples from frontal cortex including subcortical white matter were collected and paraffin embedded. All donors gave written informed consent, and the procedures were carried out in accordance with the Declaration of Helsinki (P18.164). Five-μm sections were pretreated with 0.1% trypsin for 30 min at 37°C for the NOTCH3 staining, and with 0.1M citrate buffer for 10 min for the PDGFRβ staining, and washed with PBS three times. The slides were incubated at room temperature for 2 h with a primary mouse anti-NOTCH3 antibody (clone 1E4, Millipore) or for 1 h with a primary goat anti-PDGFRβ (AF385, R&D Systems). A two-step detection system (BrightVision, ImmunoLogic, VWRKC-DPVB55HRP) was used per the manufacturer's protocol. In short, slides were post-blocked

for 15 min, washed with PBS, incubated for 30 min with an anti-mouse HRP antibody or anti-goat probe/HRP (GHP516), and washed in PBS and subsequently stained with 3,3′-diaminobenzidine (DAB) + Substrate Chromogen System (Dako, K3468, diluted 1:50). Counterstaining was then performed with 1:10 diluted Harris hematoxylin for 10 s. To assess the correlation of NOTCH3 and PDGFRβ stainings, for each brain, 20 blood vessels were identified that were present on both NOTCH3- and PDGFRβ-stained slides. The vessel walls were then manually segmented to create a region of interest (ROI) using QuPath. Within these ROIs, NOTCH3 aggregation load and PDGFRβ staining was quantified by using Color Threshold in ImageJ (settings: Hue, 40–210 [stop]; saturation, 0–255; brightness, 0–150). The NOTCH3 and PDGFRb scores per blood vessel were equal to the fraction of surface wall area positive for these respective stainings.

Statistical analysis

Statistical analyses were performed using GraphPad Prism 9 software. Normality of the data was evaluated by the D'Agostino-Pearson test. One- and two-way ANOVA with Tukey's multiple comparison test was used for the analysis of three groups. For paired or unpaired analysis of two groups, either Student's *t* test or Wilcoxon-Mann-Whitney test was used. Analyses are indicated in the figure legends. The data are reported as mean ± SD. Statistical significance was defined as $p < 0.05$. For analyzing the correlation between NOTCH3 and PDGFRβ staining, a linear mixed model with a random intercept per patient ID was created with the PDGFRb score as a dependent variable and the NOTCH3 score as an independent variable. To obtain normal distribution and homoscedasticity of residuals, both the NOTCH3 score and the PDGFRβ score were cube root transformed. Two-sided p values <0.05 were considered significant. Statistical analyses were performed in R v.4.4.0.

RESOURCE AVAILABILITY

Lead contact

Further information and requests for resources and reagents should be directed to and will be fulfilled by the lead contact, Dr. Valeria V. Orlova (v.orlova@lumc.nl).

Materials availability

hiPSC lines are available upon MTA.

Data and code availability

The bulk RNA sequencing datasets have been deposited in the European Genome-Phenome Archive with accession number EGAD50000002181.

This paper does not report original code.

Software used to analyze the data is either freely or commercially available. Any additional information associated with the data presented in this paper is available from the lead contact upon request.

ACKNOWLEDGMENTS

We thank the LUMC human iPSC Hotel for the generation and characterization of hiPSC lines and the LUMC confocal imaging facility (Lennard Voortman and Annelies Boonzaier-van der Laan) for help with imaging. Illustrations were created using BioRender.com. We thank Tessa de Korte and Ncardia for the use of the FDSS/μcell for the Ca^{2+} experiments. We would like to thank Veronica Ramovs for her helpful discussions on focal adhesions. This work was supported by the Netherlands Organization for Health Research and Development (ZonMw; VIDI 91717325); the cureCADASIL association and Orphan Disease Center (MDBR-22-126-CADASIL); Alzheimer Nederland (WE.03-2024-10); the Netherlands Organ-on-Chip Initiative, which is an NWO Gravitation project (024.003.001) funded by the Ministry of Education, Culture and Science of the government of the Netherlands; and the Novo Nordisk Foundation Center for Stem Cell Medicine, which is supported by a Novo Nordisk Foundation grant (NNF21CC0073729).

AUTHOR CONTRIBUTIONS

Conceptualization, M.V.C., J.W.R., S.A.J.L.O., and V.V.O.; methodology, M.V.C., T.T., M.N.C., J.L.G., J.W.R., S.A.J.L.O., and V.V.O.; formal analysis, M.V.C., T.T., M.N.C., and J.L.G.; software, M.V.C., M.N.C., and J.L.G.; investigation, M.V.C., T.T., M.N.C., J.L.G., F.E.v.d.H., K.L.D., G.G., and C.F. ; resources, M.V.C., A.A.F.d.V., C.L.M., J.W.R., S.A.J.L.O., and V.V.O.; writing – original draft, M.V.C., J.W.R., S.A.J.L.O., and V.V.O.; writing – review and editing, M.V.C., C.L.M., J.W.R., S.A.J.L.O., and V.V.O.; supervision, M.V.C, J.W.R., S.A.J.L.O., and V.V.O.; project administration, M.V.C, J.W.R., S.A.J.L.O., and V.V.O.; funding acquisition, M.V.C, C.L.M., J.W.R., S.A.J.L.O., and V.V.O.

DECLARATION OF INTERESTS

The authors declare no competing interests.

SUPPLEMENTAL INFORMATION

Supplemental information can be found online at https://doi.org/10.1016/j.stemcr.2026.102863.

Received: August 19, 2025
Revised: February 24, 2026
Accepted: February 26, 2026
Published: March 26, 2026

REFERENCES

Ahn, Y., An, J.H., Yang, H.J., Lee, W.J., Lee, S.H., Park, Y.H., Lee, J.H., Lee, H.J., Lee, S.H., and Kim, S.U. (2024). Blood vessel organoids generated by base editing and harboring single nucleotide variation in Notch3 effectively recapitulate CADASIL-related pathogenesis. Mol. Neurobiol. *61*, 9171–9183.

Belin de Chantemèle, E.J., Retailleau, K., Pinaud, F., Vessières, E., Bocquet, A., Guihot, A.L., Lemaire, B., Domenga, V., Baufreton, C., Loufrani, L., et al. (2008). Notch3 is a major regulator of

vascular tone in cerebral and tail resistance arteries. Arterioscler. Thromb. Vasc. Biol. *28*, 2216–2224.

Bouma, M.J., Orlova, V., van den Hil, F.E., Mager, H.J., Baas, F., de Knijff, P., Mummery, C.L., Mikkers, H., and Freund, C. (2020). Generation and genetic repair of 2 iPSC clones from a patient bearing a heterozygous c.1120del18 mutation in the ACVRL1 gene leading to Hereditary Hemorrhagic Telangiectasia (HHT) type 2. Stem Cell Res. *46*, 101786.

Bouma, M.J., van Iterson, M., Janssen, B., Mummery, C.L., Salvatori, D.C.F., and Freund, C. (2017). Differentiation-Defective Human Induced Pluripotent Stem Cells Reveal Strengths and Limitations of the Teratoma Assay and In Vitro Pluripotency Assays. Stem Cell Rep. *8*, 1340–1353.

Capone, C., Cognat, E., Ghezali, L., Baron-Menguy, C., Aubin, D., Mesnard, L., Stöhr, H., Domenga-Denier, V., Nelson, M.T., and Joutel, A. (2016). Reducing Timp3 or vitronectin ameliorates disease manifestations in CADASIL mice. Ann. Neurol. *79*, 387–403.

Cerneckis, J., Cai, H., and Shi, Y. (2024). Induced pluripotent stem cells (iPSCs): molecular mechanisms of induction and applications. Signal Transduct. Target. Ther. *9*, 112.

Chabriat, H., Joutel, A., Dichgans, M., Tournier-Lasserve, E., and Bousser, M.G. (2009). Cadasil. Lancet Neurol. *8*, 643–653.

Chen, T.W., Wardill, T.J., Sun, Y., Pulver, S.R., Renninger, S.L., Baohan, A., Schreiter, E.R., Kerr, R.A., Orger, M.B., Jayaraman, V., et al. (2013). Ultrasensitive fluorescent proteins for imaging neuronal activity. Nature *499*, 295–300.

Craggs, L.J.L., Fenwick, R., Oakley, A.E., Ihara, M., and Kalaria, R.N. (2015). Immunolocalization of platelet-derived growth factor receptor-beta (PDGFR-beta) and pericytes in cerebral autosomal dominant arteriopathy with subcortical infarcts and leukoencephalopathy (CADASIL). Neuropathol. Appl. Neurobiol. *41*, 557–570.

Domenga, V., Fardoux, P., Lacombe, P., Monet, M., Maciazek, J., Krebs, L.T., Klonjkowski, B., Berrou, E., Mericskay, M., Li, Z., et al. (2004). Notch3 is required for arterial identity and maturation of vascular smooth muscle cells. Genes Dev. *18*, 2730–2735.

Dupré, N., Drieu, A., and Joutel, A. (2024). Pathophysiology of cerebral small vessel disease: a journey through recent discoveries. J. Clin. Investig. *134*, e172841.

Dupré, N., Gueniot, F., Domenga-Denier, V., Dubosclard, V., Nilles, C., Hill-Eubanks, D., Morgenthaler-Roth, C., Nelson, M.T., Keime, C., Danglot, L., and Joutel, A. (2024). Protein aggregates containing wild-type and mutant NOTCH3 are major drivers of arterial pathology in CADASIL. J. Clin. Investig. *134*, e175789.

Gatti, J.R., Zhang, X., Korcari, E., Lee, S.J., Greenstone, N., Dean, J.G., Maripudi, S., and Wang, M.M. (2018). Redistribution of Mature Smooth Muscle Markers in Brain Arteries in Cerebral Autosomal Dominant Arteriopathy with Subcortical Infarcts and Leukoencephalopathy. Transl. Stroke Res. *10*, 160–169.

Ghezali, L., Capone, C., Baron-Menguy, C., Ratelade, J., Christensen, S., Østergaard Pedersen, L., Domenga-Denier, V., Pedersen, J.T., and Joutel, A. (2018). Notch3(ECD) immunotherapy improves cerebrovascular responses in CADASIL mice. Ann. Neurol. *84*, 246–259.

Gravesteijn, G., Hack, R.J., Mulder, A.A., Cerfontaine, M.N., van Doorn, R., Hegeman, I.M., Jost, C.R., Rutten, J.W., and Lesnik Oberstein, S.A.J. (2022). NOTCH3 variant position is associated with NOTCH3 aggregation load in CADASIL vasculature. Neuropathol. Appl. Neurobiol. *48*, e12751.

Hack, R.J., Gravesteijn, G., Cerfontaine, M.N., Hegeman, I.M., Mulder, A.A., Lesnik Oberstein, S.A.J., and Rutten, J.W. (2022). Cerebral Autosomal Dominant Arteriopathy With Subcortical Infarcts and Leukoencephalopathy Family Members With a Pathogenic NOTCH3 Variant Can Have a Normal Brain Magnetic Resonance Imaging and Skin Biopsy Beyond Age 50 Years. Stroke *53*, 1964–1974.

Hack, R.J., Gravesteijn, G., Cerfontaine, M.N., Santcroos, M.A., Gatti, L., Kopczak, A., Bersano, A., Duering, M., Rutten, J.W., and Lesnik Oberstein, S.A.J. (2023). Three-tiered EGFr domain risk stratification for individualized NOTCH3-small vessel disease prediction. Brain *146*, 2913–2927.

Halaidych, O.V., Cochrane, A., van den Hil, F.E., Mummery, C.L., and Orlova, V.V. (2019). Quantitative Analysis of Intracellular Ca(2+) Release and Contraction in hiPSC-Derived Vascular Smooth Muscle Cells. Stem Cell Rep. *12*, 647–656.

He, C., Medley, S.C., Hu, T., Hinsdale, M.E., Lupu, F., Virmani, R., and Olson, L.E. (2015). PDGFRbeta signalling regulates local inflammation and synergizes with hypercholesterolaemia to promote atherosclerosis. Nat. Commun. *6*, 7770.

Hellström, M., Phng, L.K., Hofmann, J.J., Wallgard, E., Coultas, L., Lindblom, P., Alva, J., Nilsson, A.K., Karlsson, L., Gaiano, N., et al. (2007). Dll4 signalling through Notch1 regulates formation of tip cells during angiogenesis. Nature *445*, 776–780.

Henshall, T.L., Keller, A., He, L., Johansson, B.R., Wallgard, E., Raschperger, E., Mäe, M.A., Jin, S., Betsholtz, C., and Lendahl, U. (2015). Notch3 is necessary for blood vessel integrity in the central nervous system. Arterioscler. Thromb. Vasc. Biol. *35*, 409–420.

Hussain, M.B., Singhal, S., Markus, H.S., and Singer, D.R.J. (2004). Abnormal vasoconstrictor responses to angiotensin II and noradrenaline in isolated small arteries from patients with cerebral autosomal dominant arteriopathy with subcortical infarcts and leukoencephalopathy (CADASIL). Stroke *35*, 853–858.

Ihalainen, S., Soliymani, R., Iivanainen, E., Mykkänen, K., Sainio, A., Pöyhönen, M., Elenius, K., Järveläinen, H., Viitanen, M., Kalimo, H., and Baumann, M. (2007). Proteome analysis of cultivated vascular smooth muscle cells from a CADASIL patient. Mol. Med. *13*, 305–314.

Jin, S., Hansson, E.M., Tikka, S., Lanner, F., Sahlgren, C., Farnebo, F., Baumann, M., Kalimo, H., and Lendahl, U. (2008). Notch signaling regulates platelet-derived growth factor receptor-beta expression in vascular smooth muscle cells. Circ. Res. *102*, 1483–1491.

Joutel, A., Andreux, F., Gaulis, S., Domenga, V., Cecillon, M., Battail, N., Piga, N., Chapon, F., Godfrain, C., and Tournier-Lasserve, E. (2000). The ectodomain of the Notch3 receptor accumulates within the cerebrovasculature of CADASIL patients. J. Clin. Investig. *105*, 597–605.

Joutel, A., Corpechot, C., Ducros, A., Vahedi, K., Chabriat, H., Mouton, P., Alamowitch, S., Domenga, V., Cécillion, M.,

Marechal, E., et al. (1996). Notch3 mutations in CADASIL, a hereditary adult-onset condition causing stroke and dementia. Nature *383*, 707–710.

Kelleher, J., Dickinson, A., Cain, S., Hu, Y., Bates, N., Harvey, A., Ren, J., Zhang, W., Moreton, F.C., Muir, K.W., et al. (2019). Patient-Specific iPSC Model of a Genetic Vascular Dementia Syndrome Reveals Failure of Mural Cells to Stabilize Capillary Structures. Stem Cell Rep. *13*, 817–831.

Li, X., Zhang, X., Leathers, R., Makino, A., Huang, C., Parsa, P., Macias, J., Yuan, J.X.J., Jamieson, S.W., and Thistlethwaite, P.A. (2009). Notch3 signaling promotes the development of pulmonary arterial hypertension. Nat. Med. *15*, 1289–1297.

Ling, C., Liu, Z., Song, M., Zhang, W., Wang, S., Liu, X., Ma, S., Sun, S., Fu, L., Chu, Q., et al. (2019). Modeling CADASIL vascular pathologies with patient-derived induced pluripotent stem cells. Protein Cell *10*, 249–271.

Littau, J.L., Velilla, L., Hase, Y., Villalba-Moreno, N.D., Hagel, C., Drexler, D., Osorio Restrepo, S., Villegas, A., Lopera, F., Vargas, S., et al. (2022). Evidence of beta amyloid independent small vessel disease in familial Alzheimer's disease. Brain Pathol. *32*, e13097.

Liu, H., Kennard, S., and Lilly, B. (2009). NOTCH3 expression is induced in mural cells through an autoregulatory loop that requires endothelial-expressed JAGGED1. Circ. Res. *104*, 466–475.

Machuca-Parra, A.I., Bigger-Allen, A.A., Sanchez, A.V., Boutabla, A., Cardona-Vélez, J., Amarnani, D., Saint-Geniez, M., Siebel, C.W., Kim, L.A., D'Amore, P.A., and Arboleda-Velasquez, J.F. (2017). Therapeutic antibody targeting of Notch3 signaling prevents mural cell loss in CADASIL. J. Exp. Med. *214*, 2271–2282.

Majesky, M.W. (2007). Developmental basis of vascular smooth muscle diversity. Arterioscler. Thromb. Vasc. Biol. *27*, 1248–1258.

Neves, K.B., Harvey, A.P., Moreton, F., Montezano, A.C., Rios, F.J., Alves-Lopes, R., Nguyen Dinh Cat, A., Rocchicciolli, P., Delles, C., Joutel, A., et al. (2019). ER stress and Rho kinase activation underlie the vasculopathy of CADASIL. JCI Insight *4*, e131344.

Ni, C.Y., Murphy, M.P., Golde, T.E., and Carpenter, G. (2001). gamma -Secretase cleavage and nuclear localization of ErbB-4 receptor tyrosine kinase. Science *294*, 2179–2181.

Oliveira, D.V., Coupland, K.G., Shao, W., Jin, S., Del Gaudio, F., Wang, S., Fox, R., Rutten, J.W., Sandin, J., Zetterberg, H., et al. (2023). Active immunotherapy reduces NOTCH3 deposition in brain capillaries in a CADASIL mouse model. EMBO Mol. Med. *15*, e16556.

Olson, L.E., and Soriano, P. (2011). PDGFRbeta signaling regulates mural cell plasticity and inhibits fat development. Dev. Cell *20*, 815–826.

Orlova, V.V., Drabsch, Y., Freund, C., Petrus-Reurer, S., van den Hil, F.E., Muenthaisong, S., Dijke, P.T., and Mummery, C.L. (2014). Functionality of endothelial cells and pericytes from human pluripotent stem cells demonstrated in cultured vascular plexus and zebrafish xenografts. Arterioscler. Thromb. Vasc. Biol. *34*, 177–186.

Orlova, V.V., Nahon, D.M., Cochrane, A., Cao, X., Freund, C., van den Hil, F., Westermann, C.J.J., Snijder, R.J., Ploos van Amstel, J.K., Ten Dijke, P., et al. (2022). Vascular defects associated with hereditary hemorrhagic telangiectasia revealed in patient-derived isogenic iPSCs in 3D vessels on chip. Stem Cell Rep. *17*, 1536–1545.

Orlova, V.V., van den Hil, F.E., Petrus-Reurer, S., Drabsch, Y., Ten Dijke, P., and Mummery, C.L. (2014). Generation, expansion and functional analysis of endothelial cells and pericytes derived from human pluripotent stem cells. Nat. Protoc. *9*, 1514–1531.

Romay, M.C., Knutsen, R.H., Ma, F., Mompeón, A., Hernandez, G.E., Salvador, J., Mirkov, S., Batra, A., Sullivan, D.P., Procissi, D., et al. (2024). Age-related loss of Notch3 underlies brain vascular contractility deficiencies, glymphatic dysfunction, and neurodegeneration in mice. J. Clin. Investig. *134*, e166134.

Rutten, J.W., Dauwerse, H.G., Gravesteijn, G., van Belzen, M.J., van der Grond, J., Polke, J.M., Bernal-Quiros, M., and Lesnik Oberstein, S.A.J. (2016). Archetypal NOTCH3 mutations frequent in public exome: implications for CADASIL. Ann. Clin. Transl. Neurol. *3*, 844–853.

Rutten, J.W., Haan, J., Terwindt, G.M., van Duinen, S.G., Boon, E.M.J., and Lesnik Oberstein, S.A.J. (2014). Interpretation of NOTCH3 mutations in the diagnosis of CADASIL. Expert Rev. Mol. Diagn. *14*, 593–603.

Rutten, J.W., Hack, R.J., Duering, M., Gravesteijn, G., Dauwerse, J.G., Overzier, M., van den Akker, E.B., Slagboom, E., Holstege, H., Nho, K., et al. (2020). Broad phenotype of cysteine-altering NOTCH3 variants in UK Biobank: CADASIL to nonpenetrance. Neurology *95*, e1835–e1843.

Rutten, J.W., Klever, R.R., Hegeman, I.M., Poole, D.S., Dauwerse, H.G., Broos, L.A.M., Breukel, C., Aartsma-Rus, A.M., Verbeek, J.S., van der Weerd, L., et al. (2015). The NOTCH3 score: a pre-clinical CADASIL biomarker in a novel human genomic NOTCH3 transgenic mouse model with early progressive vascular NOTCH3 accumulation. Acta Neuropathol. Commun. *3*, 89.

Tikka, S., Ng, Y.P., Di Maio, G., Mykkänen, K., Siitonen, M., Lepikhova, T., Pöyhönen, M., Viitanen, M., Virtanen, I., Kalimo, H., and Baumann, M. (2012). CADASIL mutations and shRNA silencing of NOTCH3 affect actin organization in cultured vascular smooth muscle cells. J. Cereb. Blood Flow Metab. *32*, 2171–2180.

Touyz, R.M., Alves-Lopes, R., Rios, F.J., Camargo, L.L., Anagnostopoulou, A., Arner, A., and Montezano, A.C. (2018). Vascular smooth muscle contraction in hypertension. Cardiovasc. Res. *114*, 529–539.

van Splunder, H., Villacampa, P., Martínez-Romero, A., and Graupera, M. (2024). Pericytes in the disease spotlight. Trends Cell Biol. *34*, 58–71.

Vila Cuenca, M., Cochrane, A., van den Hil, F.E., de Vries, A.A.F., Lesnik Oberstein, S.A.J., Mummery, C.L., and Orlova, V.V (2021). Engineered 3D vessel-on-chip using hiPSC-derived endothelial- and vascular smooth muscle cells. Stem Cell Rep. *16*, 2159–2168.

Villa, N., Walker, L., Lindsell, C.E., Gasson, J., Iruela-Arispe, M.L., and Weinmaster, G. (2001). Vascular expression of Notch pathway receptors and ligands is restricted to arterial vessels. Mech. Dev. *108*, 161–164.

Wardlaw, J.M., Smith, C., and Dichgans, M. (2019). Small vessel disease: mechanisms and clinical implications. Lancet Neurol. *18*, 684–696.

Wimmer, R.A., Leopoldi, A., Aichinger, M., Wick, N., Hantusch, B., Novatchkova, M., Taubenschmid, J., Hämmerle, M., Esk, C., Bagley, J.A., et al. (2019). Human blood vessel organoids as a model of diabetic vasculopathy. Nature *565*, 505–510.

Zellner, A., Scharrer, E., Arzberger, T., Oka, C., Domenga-Denier, V., Joutel, A., Lichtenthaler, S.F., Müller, S.A., Dichgans, M., and Haffner, C. (2018). CADASIL brain vessels show a HTRA1 loss-of-function profile. Acta Neuropathol. *136*, 111–125.

Zohorsky, K., Lin, S., and Mequanint, K. (2021). Immobilization of Jagged1 Enhances Vascular Smooth Muscle Cells Maturation by Activating the Notch Pathway. Cells *10*, 2089.

Stem Cell Reports, Volume *21*

Supplemental Information

3D Vessels-on-Chip using isogenic hiPSC-derived VSMCs reveal NOTCH3-driven alterations in brain small vessel disease

Marc Vila Cuenca, Theano Tsikari, Minne N. Cerfontaine, James L. Gallant, Francijna E. van den Hil, Marga J. Bouma, Kyra L. Dijkstra, Gido Gravesteijn, Antoine A.F. de Vries, Christine L. Mummery, Julie W. Rutten, Saskia A.J. Lesnik Oberstein, and Valeria V. Orlova

INVENTORY OF SUPPLEMENTARY MATERIAL

Supplemental Figures and Legends:

Figure S1. Characterization of hiPSC lines
Figure S2. Characterization of hiPSC-derived cells
Figure S3. Vessel characterization of hiPSC-derived 3D Vessel-on-Chip
Figure S4. Expression profile of hiPSC-derived 3D Vessel-on-Chip

Supplemental Tables

Table S1. DEG genes of hiPSC-VSMCs and gene ontology enrichment
Table S2. CADASIL patient information

Supplemental Videos

Video S1. 3D surface rendering of 3D Vessel-on-Chip
Video S2. Ca+2 dynamics of hiPSC-VSMCs in 3D Vessel-on-Chip

SUPPLEMENTARY FIGURES AND LEGENDS

FIGURE S1

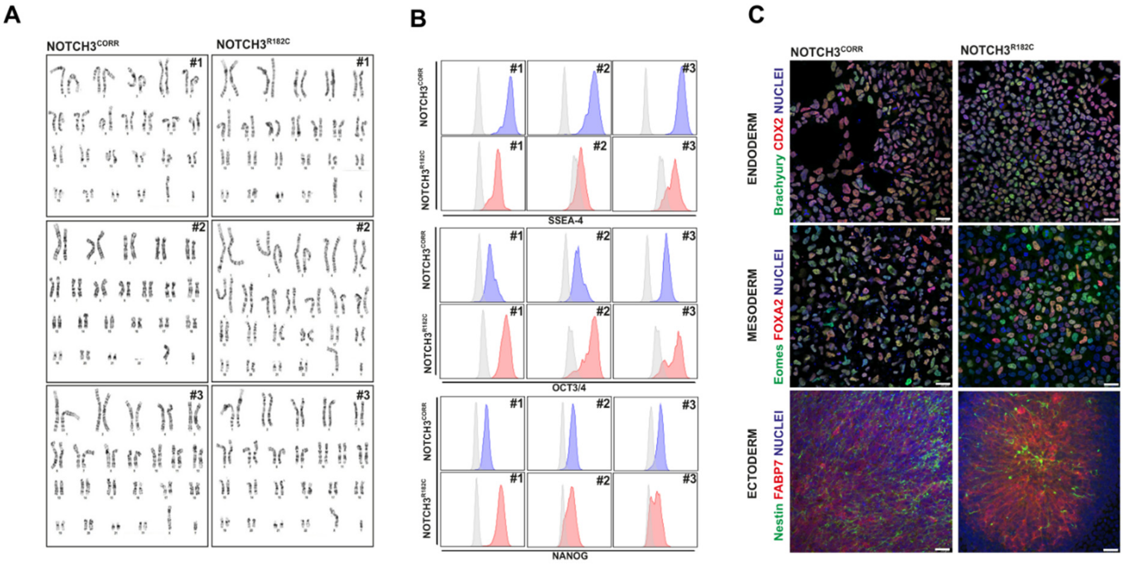

Figure S1. Characterization of hiPSC lines

(A) Karyotype of hiPSC confirmed by G-banding. (B) Expression of undifferentiated state markers SSEA4, OCT3/4 and NANOG using flow cytometry in hiPSC lines (C) Representative immunofluorescence images for markers of the three germ layers; Brachyury and CDX2 (mesoderm), FOXA2 and Eomes (endoderm) and NESTIN and FABP7 (ectoderm). Scale bar 25 μm.

FIGURE S2

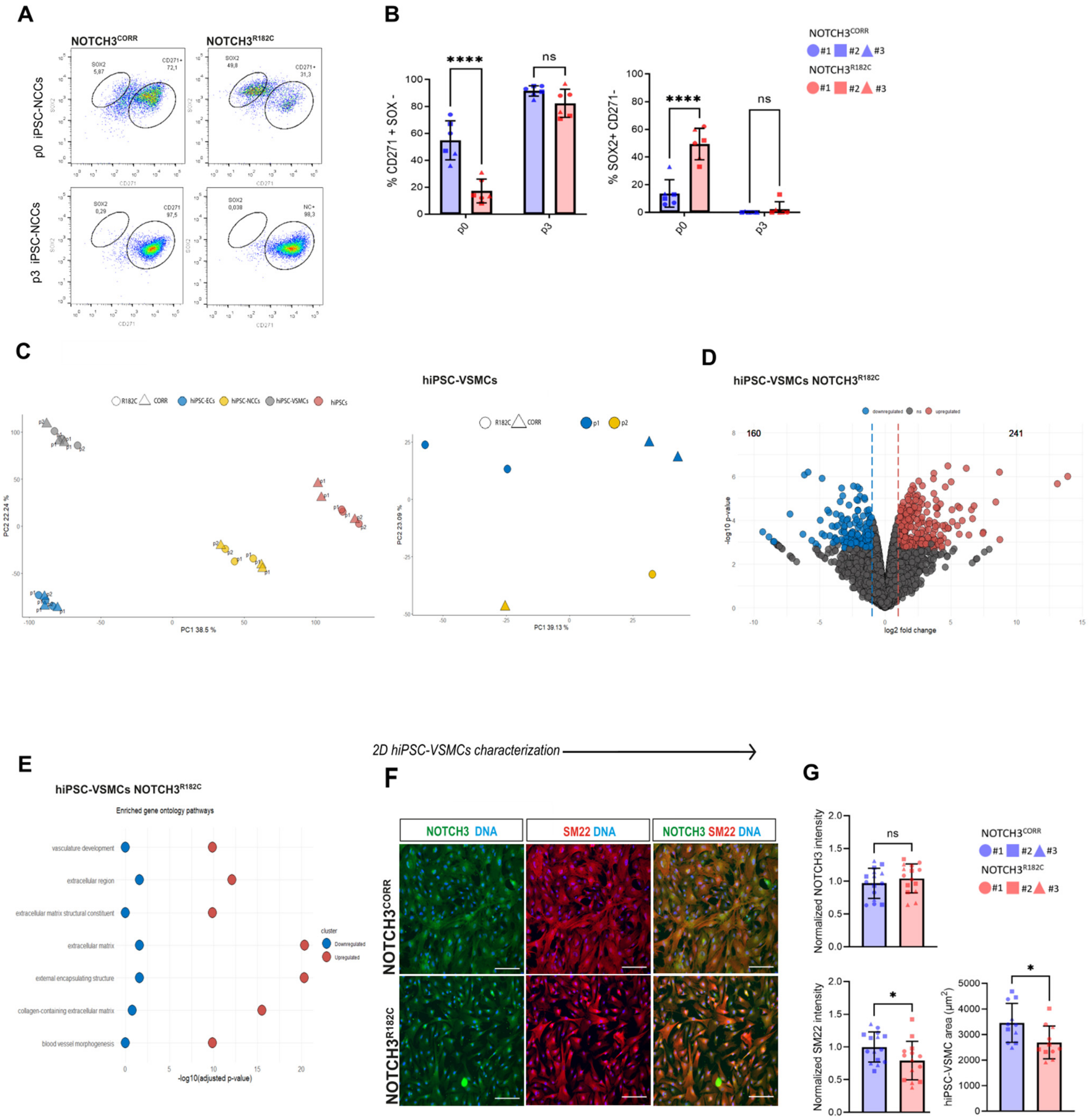

A
NOTCH3CORR
NOTCH3R182C
p0 iPSC-NCCs
p3 iPSC-NCCs
B
% CD271 + SOX -
% SOX2+ CD271-
****
ns
p0
p3
NOTCH3CORR #1 #2 #3
NOTCH3R182C #1 #2 #3
C
hiPSC-VSMCs
PC1 38.5 %
PC2 22.24 %
PC1 39.13 %
PC2 23.09 %
D
hiPSC-VSMCs NOTCH3R182C
160
241
log2 fold change
-log10 p-value
E
hiPSC-VSMCs NOTCH3R182C
Enriched gene ontology pathways
vasculature development
extracellular region
extracellular matrix structural constituent
extracellular matrix
external encapsulating structure
collagen-containing extracellular matrix
blood vessel morphogenesis
cluster
Downregulated
Upregulated
-log10(adjusted p-value)
2D hiPSC-VSMCs characterization
F
NOTCH3 DNA
SM22 DNA
NOTCH3 SM22 DNA
NOTCH3CORR
NOTCH3R182C
G
Normalized NOTCH3 intensity
Normalized SM22 intensity
hiPSC-VSMC area (µm2)
ns
*
NOTCH3CORR #1 #2 #3
NOTCH3R182C #1 #2 #3

Figure S2. Characterization of hiPSC-derived cells

(A) Representative FACS plots showing CD271 and SOX2 expression in hiPSC-NCCs at passage 0 and 3. (B) Quantification of percentage of positive population of hiPSCs (SOX2+ CD271-) and hiPSC-NCCs (SOX2- and CD271+) at passage 0 and 3 determined by FACS. Data are from N=3 three independent experiments and shown as ± SD. Two-way ANOVA test. ****$p < 0.0001$; ns, not significant. (C) Principal component (PC) analysis depicting the variation in the total gene expression by bulk RNA-seq of hiPSCs, hiPSC-ECs, hiPSC-NCCs and hiPSC-VSMCs. Color represents different cell types. The PCA is derived from log counts per million with batch effect correction applied to the replicates using limma. PC analysis of hiPSC-VSMCs comparing the two patients variance revealed a separation on the second component where 23.1 % of the total variation within the data is due to the difference in the two patients. For further processing of the data, we opted to continue with the cells obtained from patient 1 for downstream analysis. (D) Volcano plot displaying sorted log2 fold-change (FC) showing expression of genes in $NOTCH3^{R182C}$ hiPSC-VSMCs (patient 1) based on the RNA-seq profiles. Log2FC < -1 indicates downregulated genes whereas log2FC > 1 indicates upregulated genes, genes were further considered significant when the q-value < 0.05 and log counts per million > 0. (E) Select gene ontology (GO) terms enriched from significantly regulated genes using gprofiler. Enrichments were performed with DEGs from $NOTCH3^{R182C}$ hiPSC-VSMCs (Patient 1). (F) Representative immunofluorescence images showing expression of NOTCH3 (green), SM22 (red) and nuclei (DNA) of hiPSC-VSMCs. 10x, scale bars 250 µm. (G) Quantification of hiPSC-VSMCs normalized intensity of NOTCH3, SM22 and area (um^2). Data are from N=3 three independent experiments and shown as ± SD. Unpaired t test. *$p < 0.05$, ns, not significant.

FIGURE S3

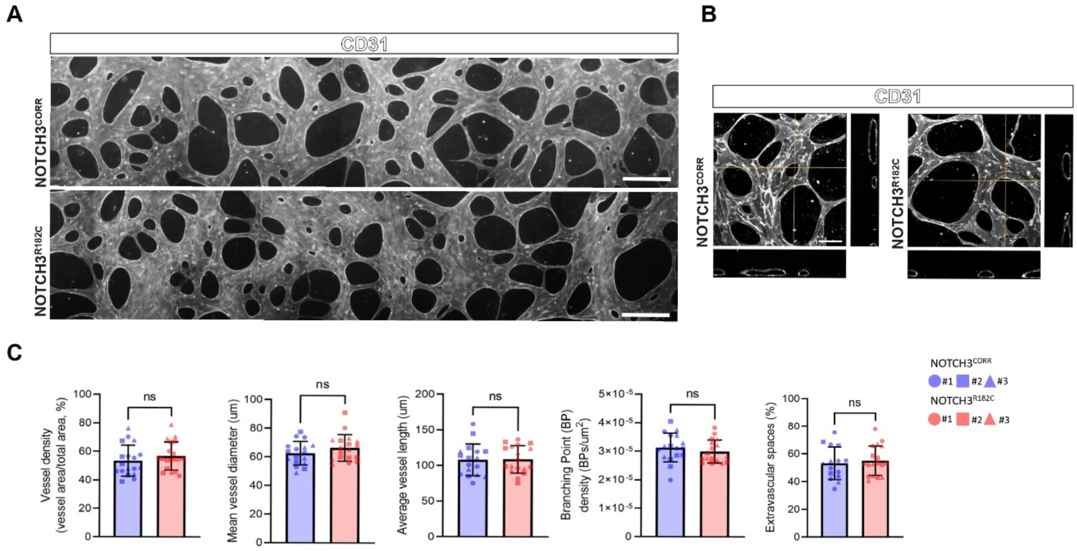

Figure S3. Vessel characterization of hiPSC-derived 3D Vessel-on-Chip.
(A) Representative images of vascular networks formed by hiPSC-ECs (CD31, grey). 10x, scale bars 200 µm. (B) Representative confocal images showing hiPSC-ECs (grey; CD31). Images displaying xyz, xy and yz cross-sectional perspectives. 40x, scale bars 100 µm. (C) Quantification of vessel density (%), mean diameter (µm), average vessel length (µm), branching point (BP) density (BPs/µm^2) and extravascular spaces (%).

FIGURE S4

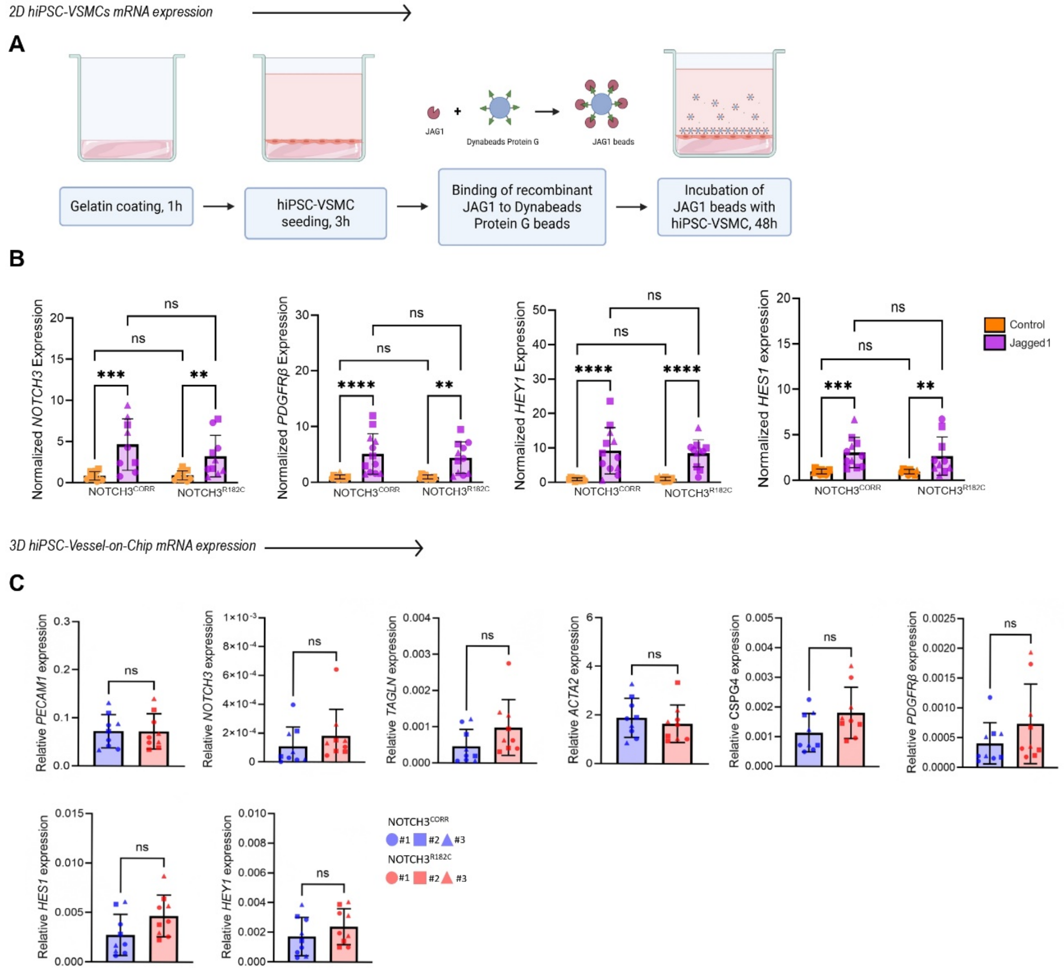

Figure S4. Expression profile of 2D hiPSC-VSMCs and hiPSC-derived 3D Vessel-on-Chip.

(A) Schematic representation of 2D Jagged1-bead assay in hiPSC-VSMCs. (B) Normalized mRNA expression of *NOTCH3, PDGFR, HES1 and HEY1 genes in 2D hiPSC-VSMCs in Control or Jagged1-activated conditions. (C)* Relative mRNA expression of *PECAM1, NOTCH3, TAGLN, CSPG4, PDGFRb, ACTA2, HES1* and *HEY* genes in 3D Vessel-on-Chip. Data are from N=3 three independent experiments and shown as ± SD. Unpaired t test. ****$p < 0.0001$, ***$p < 0.001$, **$p < 0.01$ *$p < 0.05$, ns, not significant.

METHODS

hiPSC lines

Research on hiPSC was approved by the medical ethical committee (P13.080) at Leiden University Medical Center, the Netherlands and written informed consent was obtained from all patients. PBMCs isolated from peripheral blood were used for reprogramming as described previously.(Bouma et al. 2020; Bouma et al. 2017) The following hiPSC lines were generated from Patient 1: LUMC0169iNOTCH and Patient 2: LUMC0194iNOTCH. hiPSCs were routinely cultured on Vitronectin according to the manufacturer's protocol. Pluripotency of the hiPSC clones was confirmed by expression of undifferentiated state markers OCT3/4, SSEA-4, NANOG using flow cytometry. The differentiation potential of the lines was confirmed by short-term differentiation assay in vitro, with subsequent immunofluorescence staining for markers of the three germ layers. G-banding analysis was conducted at the Laboratory of Clinical Genetics Leiden (LDGA). NIH Center for Regenerative Medicine hiPSC line (NCRM-1, generated from CD34+ cord blood cells, https://hpscreg.eu/cellline/CRMi003-A), obtained from RUDCR Infinite Biologics at Rutgers University, was modified in-house with a mCherry expression cassette under the human cytomegalovirus (hCMV) early enhancer/chicken β actin (CAG) promoter using a previously established protocol.(Rostovskaya et al. 2012)

CRISPR gene correction strategy

The heterozygous *NOTCH3* c.544C>T variant located in exon 4 was corrected by insertion of a single base (C) and simultaneous introduction of two silent variants using CRISPR/Cas9-induced homology direct repair (HDR). The targeting strategy is depicted in Figure 2A. The Streptococcus pyogenes Cas9 nuclease protein high fidelity (IDT) was used in combination with a single stranded oligo DNA (ssODN) with the corrected *NOTCH3* sequence. The silent mutations were introduced into the ssODN (5′-GTGGATGAGTGCCGGGTGGGTGAGCCCTGCCGCCATGGTGGCACCTGCCTCAACACA CCTGGCTCCTTCCGCTGTCAGTGTCCAGCTGGCTACACAGGGCCACTATGTGAGAACC CCGCGGTGCCCTGTGCGCCCTCACC -3′, IDT Ultramer) to block re-cutting of the Cas9 nuclease upon HDR and to avoid indel formation and to block restriction enzyme XcmI activity for screening. A CRISPR/Cas9 target site specific close to the variant (target site: CCA CTGTGTAGCCAGCTGGACAC) was identified using the design web tool crispor.tefor.net. CRISPR/Cas9 reagents were delivered as ribonucleoprotein (RNP) complex, composed of CRISPR/Cas9 guide RNA (crRNA:tracrRNA duplex) and the Cas9 nuclease protein (IDT). For genetic repair $1*10^5$ hiPSCs were electroporated using 2 electroporations (1300v 30ms 1 pulse) of the Neon Transfection System (Invitrogen) and subsequently cultured in 2 Synthemax II-SC (Corning)-coated wells of a 12-well plate in TESR-E8 with CloneR2 (Stem Cell Technologies). For single cell cloning, 1000 cells were plated onto a Synthemax II-SC-coated 10 cm dish in TESR-E8 with CloneR2. After 8–12 days hiPSC colonies were picked into VN-coated 2x wells of a 96-well plate in TESR-E8. The region of interest was amplified by PCR using the Terra PCR Direct Polymerase Mix (TaKaRa) from DNA isolated from one well (QuickExtract solution, Lucigen). Successfully edited clones were identified by enzyme XcmI activity and confirmed by Sanger sequencing performed by the Leiden Genome Technology Centre (LGTC).

Differentiation of hiPSCs towards ECs

hiPSCs were maintained in mTeSR-E8 and differentiated towards ECs using previously published protocols.(Orlova, van den Hil, et al. 2014; Orlova, Drabsch, et al. 2014) For mesoderm induction (day 0-3), mTeSR-E8 medium was replaced with B(P)EL medium supplemented with 8 μM CHIR99021 (Tocris Bioscience, 4423). Cells were refreshed with vascular specification medium comprised of VEGF (50 ng/ml) and 10 μM SB431542 (Tocris Bioscience, 1614) in B(P)EL at day 3, day 6, and day 9. hiPSC-ECs were isolated on day 10 using CD31-Dynabeads™ (Thermo Fisher Scientific), as previously described (Orlova et al., 2014b; 2014a). hiPSC-ECs were expanded in complete EC growth medium comprised of

Human Endothelial-serum free medium (EC-SFM) with 1% Human platelet poor serum (P2918, Sigma), VEGF (30 ng/ml) and bFGF (20 ng/ml), as described previously with minor modifications.(Orlova, van den Hil, et al. 2014; Orlova, Drabsch, et al. 2014) hiPSC-ECs were expanded for additional 3-4 days post-isolation and cryopreserved using serum-free cryopreservation medium at passage number 1 (P1) (CryoStor™CS10) (StemCell Technologies, 07930).

Differentiation of hiPSCs towards NCCs

hiPSC colonies were passaged and kept in hiPSC mTeSR-E8 and differentiated towards NCCs using previously published protocols.(Halaidych et al. 2019) After 2 days, the medium was changed to NC differentiation medium consisting of B(P)EL medium supplemented with 10 µM SB431542 (Tocris Bioscience, 1614), 1 µM CHIR99021 (Tocris Bioscience, 4423) and 10 ng/mL bFGF (Miltenyi Biotec, 130-093-842). Cells were refreshed every 2 days and kept in NC differentiation medium for 10-12 days. After 10-12 days NC cells (NCCs) were passaged with 1xTrypLE Select (Gibco, 12563029) and plated in 1:4 ratio on Matrigel-coated plates. hiPSC-NCCs were cryopreserved at P3 using serum-free cryopreservation medium (CryoStor™CS10) (StemCell Technologies, 07930).

Differentiation of hiPSC-NCCs towards VSMCs

hiPSC-NCCs were differentiated into VSMCs following a previously described protocol with minor modifications.(Halaidych et al. 2019) NCCs were plated at $3x10^4$ cells/cm^2 seeding density on 0.1% Gelatin (Sigma-Aldrich, G1890) coated plates in VSMC differentiation medium consisting of B(P)EL medium supplemented with 2 ng/mL TGF-β3 (PeproTech, 100-36E) and 10 ng/mL PDGF-BB (PeproTech, 100-14B). Cells were refreshed every 2 days and kept in VSMC differentiation medium for 8 days. Cells were passaged in a 1:4 splitting ratio at day 4. hiPSC-VSMCs were cryopreserved at P1 using serum-free cryopreservation medium (CryoStor™CS10) (StemCell Technologies, 07930).

Primary VSMCs culture

Primary brain VSMCs were isolated from post-mortem brain tissue. Briefly, connective tissue and arachnoid mater surrounding the arteries was removed. Arteries were washed in PBS and cut into 0.5 cm pieces and dissected longitudinally. The endothelial layer was removed mechanically by scraping across the bottom of a Petri dish and the arterial sections were fixated to the bottom of a sterile T25 flask containing 4 ml complete culture medium (DMEM-F12, glutaMAX, supplemented with 10% FCS, 2 µM MEM sodium pyruvate, 0.5 U/ml penicillin and 0.5 µg/ml streptomycin (all from Life Technologies, Bleiswijk, The Netherlands). Flasks were first incubated in an upright position at 5% CO_2, 37°C, to ensure tissue binding. After 4 hours the flasks were placed in a horizontal position to cover the arterial sections with culture medium. First cellular outgrowth was seen 2 weeks after isolation. Cells were cryopreserved at P3-4 using culture medium supplemented with 20% fetal calf serum and 10% DMSO.

RNA sequencing analysis

Total RNA was extracted using the NucleoSpin RNA XS kit (Macherey-Nagel, cat no. 740902.50) according to the manufacturer's instructions. Whole transcriptome data were generated at Novogene (Cambridge, UK) using the Illumina Sequencing PE150 (PE150, Q30≥85 %). RNAseq reads were processed using the opensource BIOWDL RNAseq pipeline v5.0.0 (biowdl/RNA-seq: Release 5.0.0 (zenodo.org) developed at the LUMC.[7] This pipeline performs FASTQ pre-processing (including quality control, quality trimming, and adapter clipping), alignment, read quantification, and optionally transcript assembly. FastQC (v0.11.9) was used for checking raw read QC. Adapter clipping was performed using Cutadapt (v2.10) with the default settings. RNAseq reads' alignment was performed using STAR (v2.7.5a) on human reference genome GRCh38. The gene read quantification was performed using HTSeq-count (v0.12.4) with the Ensembl gene annotation version 110.(Anders, Pyl, and Huber 2015) The resulting count matrix was transformed into log counts per million (cpm) and

the sources of variation was inspected with principal component analysis. It was observed that 23.1 % of the total variation within the data is due to the difference in the two patients. It is thus clear that the difference in the genetic make up between the two patients is responsible for a large source of variation thereby introducing confounding factors. For further processing of the data, we opted to continue with the cells obtained from patient 1 for downstream analysis. The RNAseq data was further processed in R (version 4.3.3) using the Bioconductor package edgeR.[8] Briefly, the genes with low counts were filtered by their expression, requiring expression in at least one of the conditions followed by normalization of the library using trimmed mean of the M-values (TMM) and the dispersion was estimated using the quantile-adjusted conditional maximum likelihood (qCML) method. Finally, differential gene expression was determined using likelihood ratio tests and multiple hypothesis testing was corrected for using Benjamini-Hochberg False discovery rate. The differentially expressed genes were defined based on a log fold change > 1 or < -1, a q-value < 0.05 and a logCPM > 0. Plots depicting differential gene expression was also created in the same R version using ggplot2. Gene ontology enrichment analysis was used to assess biological functions based on gene expression by using the differentially expressed genes as defined above. Enrichments were calculated using gprofiler using both the up and down regulated genes using the default parameters.(Kolberg et al. 2023) The full list of differentially expressed genes and gprofiler enrichment is available in table S1.

FACS NCCs

Cells were dissociated with 1xTrypLE Select and washed once with FACs buffer containing 10% FBS, and once with FACs buffer. The following surface antibody NGFRBV421 (BD Biosciences, 562562, 1:100) was used. For intracellular labelling with SOX2-A488 (eBiosciences, 53-9811-80, 1:50) the cells were fixed and permeabilized using BD Cytofix/Cytoperm kit (BD Biosciences, 554714). Analysis of samples was performed on the MACSQuant VYB (Miltenyi Biotec, 130-096-116) equipped with the following lasers/filters: Violet/405 nm BV421: 450/50, Blue/488 nm FITC, A488: 525/50, Yellow/561 nm PE: 586/15, Yellow/561 nm PEVio-770: 750 nm LP.

Immunofluorescence 2D staining and microscopy

VSMCs were grown in Fibronectin-coated 96 well plates (Corning, 353219) to a confluent monolayer. Cells were fixed with 4% PFA, permeabilized with 0.05% TX-100 (Sigma). the following antibodies were used: Primary antibodies NOTCH3 (Sigma, 1E4, 1:200); SM22 (Abcam, ab14106, 1:200). VSMCs were imaged using EVOS M7000 using 10x magnification objective. Images were quantified using pipelines developed on the free open source CellProfiler software (https://cellprofiler.org/).(Carpenter et al. 2006)

Assessment of 2D contractile hiPSC-VSMCs properties

hiPSC-VSMCs were passaged as single cells and plated in a Gelatine-coated 96 well plate at density ~$2*10^4$ cells/cm2 in B(P)EL medium and kept in a CO2 incubator overnight before functional analysis as previously described.(Halaidych et al. 2019) Cells were loaded with 2 µM Calcein AM (Ex/Em=494/517 nm, Invitrogen L3224) for 30 min in a live imaging chamber (37°C, 5% CO2, humidified). After the staining cells were gently washed with B(P)EL medium before assessment of the contraction. Series of images of Calcein fluorescence were captured using a Leica AF6000 microscope with a 10x objective and 4x4 automated stitching. First, the basal state of cells was acquired. Then a negative control was obtained by adding B(P)EL medium and fluorescence was acquired after 30 min. Finally, cells were stimulated with ET-I at a final concentration of 0.1 µM and fluorescence was acquired after 30 minutes. Images were processed using a customized pipeline that included automated cell identification and tracking using CellProfiler as previously described.(Halaidych et al. 2019) Output data were analyzed using a customized R-based script.

Assessment of 2D hiPSC-VSMCs Intracellular Ca2+ release

hiPSC-VSMCs were passaged as single cells and plated in a bottomed Fibronectin-coated 96-well plate at density ~5*10^4 cells/cm2 in B(P)EL medium and kept in a CO2 incubator overnight before functional analysis Intracellular Ca2+ release was assessed in hiPSC-VSMCs at day one post-seeding in a black, flat-. The calcium-6 dye (Molecular Devices) was dissolved in 10 mL HBSS buffer B and subsequently diluted 1:4 in Buffer B (Molecular Devices). The diluted dye solution was added 1:1 to the wells containing B(P)EL medium. hiPSC-VSMCs were incubated for 2 hours at 37 °C with 5% CO2 before being measured on the FDSS/µcell (Hamamatsu Photonics) at 37 °C with an exposure time of 0.1s. Response to ET-I stimulus was performed by first preparing a "compound plate" including a medium control of B(P)EL medium and 1 µM ET-I (Sigma, A9187) in B(P)EL medium. Control and ET-I stimulus were automatically mixed and injected (20ul)into the assay plate (180 ul per well), reaching final ET-I concentrations of 0.1 µM. Analysis was performed in R (4.0.3) and the induced change in Ca2+ release was calculated by quantification of the area under the curve of the average fluorescence intensity normalized to time 0 (s).

Activation of NOTCH3 signalling in 2D hiPSC-VSMCs

Activation of the NOTCH3 signalling pathway in 2D using Jagged1 beads was performed according as previously described with minor modifications.(Zohorsky, Lin, and Mequanint 2021) hiPSC-VSMC were seeded on gelatin-coated plates in EGM-2 medium at a density of 20.000 cells/cm^2 and were cultured for 3 hours to ensure cell attachment. For the preparation of the Jagged1 beads, 600 ug of Dynabeads Protein G (30 mg/ml, Invitrogen) were washed with PBS-T 0.02% and then incubated with 2.5 ug of recombinant Jagged1-FC chimera (diluted in PBS-T 0.02%, R&D systems) for 10 minutes under rotation in room temperature. Afterwards, Jagged1 beads were washed with PBS and resuspended in EGM-2 medium. Finally, the cells were incubated with Jagged1 beads for 48 hours at a concentration of 18 nM; plain Dynabeads Protein G beads were used as a negative control. After 48 h, the cells were harvested for RNA isolation and downstream qPCR analysis.

Cell preparation prior 3D Vessel-on-Chip culture

hiPSC-ECs (P1) were thawed and cultured on gelatin-coated plates in complete EC growth medium composed of Human Endothelial-SFM (EC-SFM) with 1% platelet poor serum (PPS), VEGF (30 ng/ml) and bFGF (20 ng/ml),4 days prior to 3D Vessel-on-Chip seeding. hiPSC-VSMCs (P1) were thawed and cultured on gelatin-coated plates in B(P)EL medium supplemented with 2 ng/mL TGF-β3 (PeproTech, 100-36E) and 10 ng/mL PDGF-bb (PeproTech, 100-14B) 4 days prior to 3D Vessel-on-Chip seeding using previously described protocol with minor modifications.(Halaidych et al. 2019) Primary VSMCs (P4-6) were thawed and cultured on gelatin-coated plates in complete culture medium composed of DMEM-F12, glutaMAX, supplemented with 10% FCS, 2 µM MEM sodium pyruvate, 0.5 U/ml penicillin and 0.5 µg/ml streptomycin (all from Life Technologies, Bleiswijk, The Netherlands).

Immunofluorescence 3D staining, Microscopy and analysis

After 7 days of culture in, cells in 3D Vessel-on-Chip were fixed *in situ* in 4% paraformaldehyde (PFA) for 30 min at RT. Cell plasma membranes were permeabilized with 0.5% Triton X-100 for 15 min at RT and washed 3 times for 10 mins between each step with PBS, then blocking buffer (2% BSA) was added for 3 hours at RT. Primary antibodies (1:200 volume ratio in 1% BSA), against CD31 (PECAM1, Mouse, M0823, DAKO or Sheep, AF806, R&D systems), VE-Cadherin (Rabbit, 2158S, Cell Signaling), NOTCH3 (Mouse, 1E4, Sigma-Aldrich) SM22 (TAGLIN; Rabbit, ab14106, Abcam), PDGFRb (Goat, AF385, R&D systems), aSMA (ACTA2; Mouse, 1A4, Sigma-Aldrich) and Integrin β1 (CD29; TS2/16, Thermo-Fisher) were incubated overnight at 4 ºC. Secondary antibodies (1:300 volume ratio in 1% BSA) and F-Actin (Phalloidin labelling probe GFP; 1:100, Thermo-Fisher), were incubated for 2 hours at RT after 3 times 15 min PBS washes. Vessel-on-Chip were imaged using EVOS M7000 using 10x magnification objective. A customised plate layout that allowed for automated imaging and stitching to produce images of complete microfluidic channel for all fluorescent channels was used. Images from the whole microfluidic channel (acquired using EVOS) were quantified

using pipelines developed on the free open source CellProfiler software (https://cellprofiler.org/) as previously described.(Vila Cuenca et al. 2021; Orlova et al. 2022) Two filter steps were applied to images of vascular network to reduce non-specific segmentation from cell junctions and a minimum cross-entropy thresholding method was used to produce a binarized image. The binarized images from the CellProfiler output were then analyzed using the freely available ImageJ software with the plugin (https://imagej.nih.gov/ij/ ,https://imagej.net/DiameterJ).(Hotaling et al. 2015) For 3D stacks, images were taken using a DragonFly spinning disk (Andor) microscope with 40x and 63x magnification objective and post-processing performed and processed using Imaris 9.5 software (Bitplane, Oxford Instruments). For 3D quantitative analysis, surface-rendering was performed and processed using Imaris 9.5 software (Bitplane, Oxford Instruments) as previously described.(Vila Cuenca et al. 2021; Orlova et al. 2022) VSMCs morphological analysis were performed with the visually aided morphophenotyping image recognition VAMPIRE software.(Phillip et al. 2021)

RNA isolation and quantitative RT-PCR

Total RNA was isolated from the microfluidic devices at end-point day 7 as previously described. Cells were extracted by dissolving the extracellular matrix / fibrin mix with Collagenase B (1 mg/ml, Roche, 11088815001) for half an hour at 37 degrees °C, while rocking. RNA was extracted using the NucleoSpin RNA XS kit (Macherey-Nagel) and cDNA was synthesized using an iScript-cDNA Synthesis kit (Bio-Rad). iTaq Universal SYBR Green Supermixes (Bio-Rad) and Bio-Rad CFX384 real-time system were used for the PCR reaction and detection. Relative gene expression was calculated using the delta Ct calculation and normalized to the housekeeping gene hARP.

LV production

LV particles were produced essentially as described previously(Liu et al. 2018) except that PEI MAX 40K (Polysciences Europe, Hirschberg an der Bergstraße, Germany) instead of PEI 25K was used as transfection agent and the polyethyleneimine-DNA complexes were left on the cells for only 4 hours.

Plasmid constructs

The lentiviral vector (LV) shuttle plasmid pLV.hCMV-IE.GCaMP6f.IRES.PurR.hHBVPRE was generated in a multistep procedure using pGP-CMV-GCaMP6f (Addgene, Watertown, MA; plasmid number 40755) and pLV.hCMV-IE.IRES.PurR.hHBVPRE as starting constructs.(Neshati et al. 2014) pLV.hCMV-IE.GCaMP6f.IRES.PurR.hHBVPRE contains a human cytomegalovirus immediate-early gene (hCMV-IE) promoter driving expression of a bicistronic mRNA encoding the ultra-sensitive $[Ca^{2+}]_{cyt}$ sensor GCaMP6f ref and *Streptomyces alboniger* puromycin-N-acetyltransferase. The LV shuttle plasmid pLV.hCMV-IE.eGFP.PurR.hHBVPRE was generated by insertion of the *Aequorea victoria* enhanced green fluorescent protein (eGFP)-encoding 754-bp SmaI×EcoRI fragment of pEGFP (Clontech - Takara Bio Europe, Saint-Germain-en-Laye, France) behind the hCMV-IE promoter of pLV.hCMV-IE.IRES.PurR.hHBVPRE. To this end, the insert was combined with the 8122-bp SmaI×EcoRI fragment of pLV.hCMV-IE.IRES.PurR.hHBVPRE.(Neshati et al. 2014) Recombinant plasmid construction was done with enzymes from New England Biolabs (Bioké, Leiden, the Netherlands) or Fermentas (ThermoFisher Scientific) using standard procedures or following the instructions provided with specific reagents. The plasmids were amplified in *Escherichia coli* GeneHogs (ThermoFisher Scientific) cells and purified using LabNed Plasmid Maxiprep Kits (ITK diagnostics, Uithoorn, the Netherlands).

LV transduction of hiPSC-VSMCs

The LV shuttle plasmid pLV.hCMV.-IE.GCaMP6f(+).IRES.PurR.hHBVPRE was used to express GCaMP6f in LUMC0054iCTRL hiPSC-NCCs P3 at previously described.(Vila Cuenca et al. 2021) The LV shuttle plasmid pLV.hCMV.-IE.eGFP.IRES.PurR.hHBVPRE) was used to express pEGFP in LUMC0054iCTRL hiPSC-NCCs P3. Briefly, one day after seeding 40.000 cells/12-well on Matrigel-coated plates, hiPSC-NCCs were transduced with 2.5 µl viral

particles in B(P)EL medium overnight. 96h post-transduction with complete NC differentiation medium infected cells were selected with 1 µg/mL puromycin (Sigma, P7255). After 4 days, remaining cells were expanded (1:3 ratio) Matrigel-coated plates then dissociated with 1xTrypLE Select and cryopreserved at passage number 3 (P3) using serum-free cryopreservation medium (CryoStor™CS10) (StemCell Technologies, 07930). Next, hiPSC-NC cells (hiPSC-NCCs) were differentiated into hiPSC-VSMCs as described previously with minor modifications.(Vila Cuenca et al. 2021)

Assessment of intracellular Ca^{2+} release in the 3D Vessel-on-Chip

Intracellular Ca^{2+} release upon medium refreshment and upon stimulation with the vasoconstrictor (ET-I) was analyzed on day 7 of Vessel-on-Chip culture as preformed previously.(Vila Cuenca et al. 2021) The generation and transduction in hiPSC-VSMCs of the lentiviral vector shuttle plasmid pLV.hCMV-IE.GCaMP6f.IRES.PurR.hHBVPRE is described in Supplemental Methods. Sequences of images prior to- (basal state) and after medium refreshment were captured using EVOS M7000 with a 10x objective. For the medium refreshment, medium from all ports of the microfluidic channel was first removed and gravity-driven flow was induced by the addition of 100 µl medium to the right media ports and 50 µl medium to left connecting media ports. After 30s, fluorescence activity of the whole microfluidic channel was captured. For real-time intracellular Ca^{2+} release upon stimulation with the vasoconstrictor, the microfluidic chip was placed into a humidified live cell imaging chamber (+37°C, 5% CO_2) and mounted on a DragonFly spinning disk microscope (Andor) with a 20x magnification objective on day 7 of culture. First, medium from all ports was removed and refreshed with 30 µl of EGM-2. After 30 min, basal fluorescence activity was captured for 5 seconds. Next, gravity-driven flow was induced by the addition of 60 µl EGM-2 or EGM-2 supplemented with 1.5 µM ET-I (Sigma) with a final concentration of 1 µM after the addition to the right medium ports containing 30 µl of EGM-2. Then, simultaneous image capturing was continued for 160 seconds. Image sequences of fluorescence were captured at 4 frames per second. After simultaneous image capturing, confocal images were acquired to create a 3D stack and processed using Imaris 9.5 software (Bitplane, Oxford Instruments). Images sequences were processed using a freely available plugin ‘‘LC Pro’’ for ImageJ (https://imagej.nih.gov/ij/plugins/lc-pro/index.html).(Yip and Sham 2012) Free open-source CellProfiler software (https://cellprofiler.org/) was used to determine the total number of cells in a field of view. Output data were analysed as previously described.(Vila Cuenca et al. 2021; Halaidych et al. 2019)

REFERENCES

Anders, S., P. T. Pyl, and W. Huber. 2015. 'HTSeq--a Python framework to work with high-throughput sequencing data', *Bioinformatics*, 31: 166–9.

Bouma, M. J., V. Orlova, F. E. van den Hil, H. J. Mager, F. Baas, P. de Knijff, C. L. Mummery, H. Mikkers, and C. Freund. 2020. 'Generation and genetic repair of 2 iPSC clones from a patient bearing a heterozygous c.1120del18 mutation in the ACVRL1 gene leading to Hereditary Hemorrhagic Telangiectasia (HHT) type 2', *Stem Cell Res*, 46: 101786.

Bouma, M. J., M. van Iterson, B. Janssen, C. L. Mummery, D. C. F. Salvatori, and C. Freund. 2017. 'Differentiation-Defective Human Induced Pluripotent Stem Cells Reveal Strengths and Limitations of the Teratoma Assay and In Vitro Pluripotency Assays', *Stem Cell Reports*, 8: 1340–53.

Carpenter, A. E., T. R. Jones, M. R. Lamprecht, C. Clarke, I. H. Kang, O. Friman, D. A. Guertin, J. H. Chang, R. A. Lindquist, J. Moffat, P. Golland, and D. M. Sabatini. 2006. 'CellProfiler: image analysis software for identifying and quantifying cell phenotypes', *Genome Biol*, 7: R100.

Halaidych, O. V., A. Cochrane, F. E. van den Hil, C. L. Mummery, and V. V. Orlova. 2019. 'Quantitative Analysis of Intracellular Ca(2+) Release and Contraction in hiPSC-Derived Vascular Smooth Muscle Cells', *Stem Cell Reports*, 12: 647–56.

Hotaling, N. A., K. Bharti, H. Kriel, and C. G. Simon, Jr. 2015. 'DiameterJ: A validated open source nanofiber diameter measurement tool', *Biomaterials*, 61: 327–38.

Kolberg, L., U. Raudvere, I. Kuzmin, P. Adler, J. Vilo, and H. Peterson. 2023. 'g:Profiler-interoperable web service for functional enrichment analysis and gene identifier mapping (2023 update)', *Nucleic Acids Res*, 51: W207–W12.

Liu, J., L. Volkers, W. Jangsangthong, C. I. Bart, M. C. Engels, G. Zhou, M. J. Schalij, D. L. Ypey, D. A. Pijnappels, and A. A. F. de Vries. 2018. 'Generation and primary characterization of iAM-1, a versatile new line of conditionally immortalized atrial myocytes with preserved cardiomyogenic differentiation capacity', *Cardiovasc Res*, 114: 1848–59.

Neshati, Z., J. Liu, G. Zhou, M. J. Schalij, and A. A. de Vries. 2014. 'Development of a lentivirus vector-based assay for non-destructive monitoring of cell fusion activity', *PLoS One*, 9: e102433.

Orlova, V. V., Y. Drabsch, C. Freund, S. Petrus-Reurer, F. E. van den Hil, S. Muenthaisong, P. T. Dijke, and C. L. Mummery. 2014. 'Functionality of endothelial cells and pericytes from human pluripotent stem cells demonstrated in cultured vascular plexus and zebrafish xenografts', *Arterioscler Thromb Vasc Biol*, 34: 177–86.

Orlova, V. V., D. M. Nahon, A. Cochrane, X. Cao, C. Freund, F. van den Hil, C. J. J. Westermann, R. J. Snijder, J. K. Ploos van Amstel, P. Ten Dijke, F. Lebrin, H. J. Mager, and C. L. Mummery. 2022. 'Vascular defects associated with hereditary hemorrhagic telangiectasia revealed in patient-derived isogenic iPSCs in 3D vessels on chip', *Stem Cell Reports*, 17: 1536–45.

Orlova, V. V., F. E. van den Hil, S. Petrus-Reurer, Y. Drabsch, P. Ten Dijke, and C. L. Mummery. 2014. 'Generation, expansion and functional analysis of endothelial cells and pericytes derived from human pluripotent stem cells', *Nat Protoc*, 9: 1514–31.

Phillip, J. M., K. S. Han, W. C. Chen, D. Wirtz, and P. H. Wu. 2021. 'A robust unsupervised machine-learning method to quantify the morphological heterogeneity of cells and nuclei', *Nat Protoc*, 16: 754–74.

Rostovskaya, M., J. Fu, M. Obst, I. Baer, S. Weidlich, H. Wang, A. J. Smith, K. Anastassiadis, and A. F. Stewart. 2012. 'Transposon-mediated BAC transgenesis in human ES cells', *Nucleic Acids Res*, 40: e150.

Vila Cuenca, M., A. Cochrane, F. E. van den Hil, A. A. F. de Vries, S. A. J. Lesnik Oberstein, C. L. Mummery, and V. V. Orlova. 2021. 'Engineered 3D vessel-on-chip using hiPSC-derived endothelial- and vascular smooth muscle cells', *Stem Cell Reports*, 16: 2159–68.

Yip, K. P., and J. S. Sham. 2012. 'Tracking stars: automated two-dimensional analysis of Ca(2)(+) events. Focus on "Automated region of interest analysis of dynamic Ca(2)(+) signals in image sequences"', *Am J Physiol Cell Physiol*, 303: C233–5.

Zohorsky, K., S. Lin, and K. Mequanint. 2021. 'Immobilization of Jagged1 Enhances Vascular Smooth Muscle Cells Maturation by Activating the Notch Pathway', *Cells*, 10.
